# Synthesis of Chiral Spiro-2-pyrrolidinones with Anti-Inflammatory Properties

**DOI:** 10.1021/acsomega.6c04526

**Published:** 2026-06-26

**Authors:** Karla D. Torres-Muñoz, Diego A. Cruz-Aguilar, Antonio Nieto-Camacho, Marcos Hernández-Rodríguez

**Affiliations:** 7180 Universidad Nacional Autónoma de México, Instituto de Química, Circuito Exterior, Ciudad Universitaria, Alc. Coyoacán, Ciudad de México 04510, México

## Abstract

Spiro motifs are privileged three-dimensional scaffolds that offer a balance of rigidity and flexibility. Herein, we report the synthesis of spiro-2-pyrrolidinones via a Michael addition reaction promoted by bifunctional organocatalysts, involving cyclic 1,3-ketoesters and nitroalkenes to generate products bearing two stereocenters. Through a sequence involving ketone reduction, nitro-group reduction followed by lactamization in a one-pot procedure, and finally oxidation, requiring a single chromatographic purification, we obtained 18 chiral spiro-2-pyrrolidinones with different substituents in the lactam ring and different rings spiro-fused to the 2-pyrrolidinone moiety. The resulting compounds were evaluated for *in vivo* anti-inflammatory activity using a mouse ear edema model induced by 12-*O*-tetradecanoylphorbol-13-acetate (TPA), showing similar or slightly improved activity compared with celecoxib. Additionally, myeloperoxidase activity was measured to evaluate leukocyte infiltration, and *in vitro* inhibition assays of COX-1 and COX-2 were performed for three selected compounds, which showed selective inhibition of COX-2.

## Graphical abstract

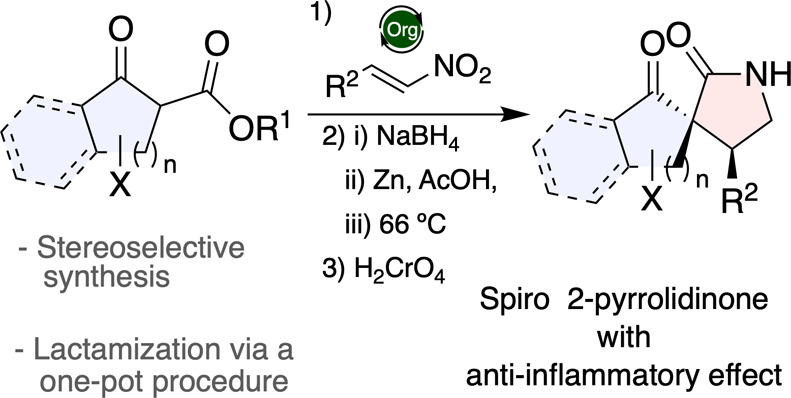

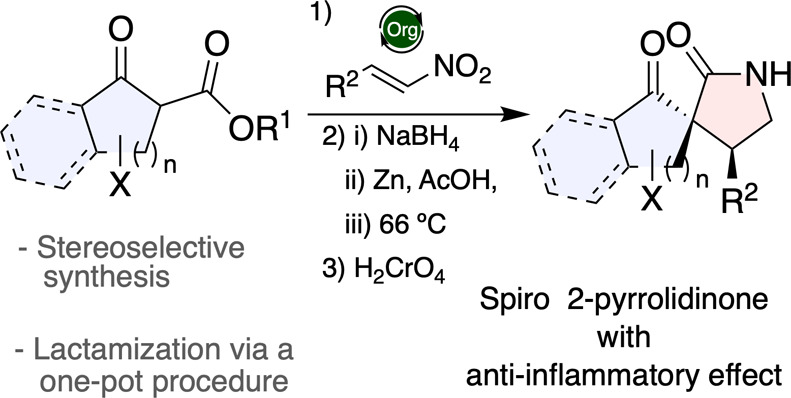

## Introduction

Spirocycles are saturated bicyclic structures in which two rings share a single atom, usually carbon. These scaffolds have a balance between rigidity and flexibility that attracts considerable attention in medicinal chemistry because biological targets recognize drugs in a three-dimensional manner. Consequently, a molecule with a preorganized 3D shape often exhibits increased binding affinity—by reducing the entropic penalty associated with conformational flexibility—as well as improved specificity, avoiding the promiscuity commonly associated with flat, aromatic systems. Another important feature of spirocycles is the wide range of exit vectors they provide, spanning all three spatial dimensions. These have proven advantageous in tuning physicochemical properties such as lipophilicity and metabolic stability, increasing at the same time molecular complexity and enabling an effective optimization and securing intellectual property around bioactive scaffolds.^1–4^

2-Pyrrolidinones exhibit a wide range of biological activities,^5^ this is also true for spiro-2-pyrrolidinones. Notable examples include rolapitant, which is used for the prevention of chemotherapy-induced nausea and vomiting; fluspirilene, employed in the treatment of schizophrenia; and fenspiride, which has been used as an antitussive. In addition, spirotetramat is widely used as an insecticide in agriculture (Chart [Fig chart1]).

**1 chart1:**
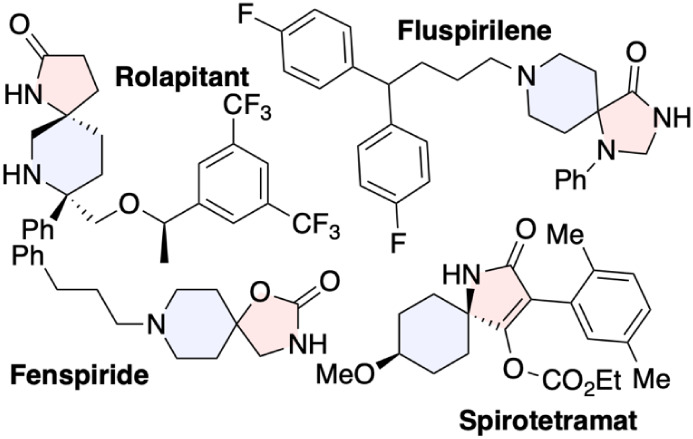
Spiro-2-pyrrolidinones with Biological Activity

There are different approaches to the synthesis of chiral spiro-2-pyrrolidinones.^6–8^ Among them, the organocatalyzed Michael addition of 1,3-dicarbonyl compounds to β-nitrostyrenes stands out as a particularly reliable strategy.^9^ Thus, we projected that a stereoselective Michael addition of cyclic 1,3-ketoesters to nitroalkenes would furnish an intermediate bearing two contiguous stereocenters, one of which is quaternary. Subsequently, the ketone must be reduced prior to reduction of the nitro group to avoid the formation of the pyrrolidine by the reaction with the more electrophilic ketone. Further lactamization and oxidation^10^ of the alcohol then delivers the desired spiro-2-pyrrolidinone (Scheme 1). Since these molecules represent novel scaffolds with no precedent in the literature regarding biological activity, we investigated their biological properties using a trial-and-error approach, as is often done during the initial evaluation of newly identified natural products.

**Scheme 1:**
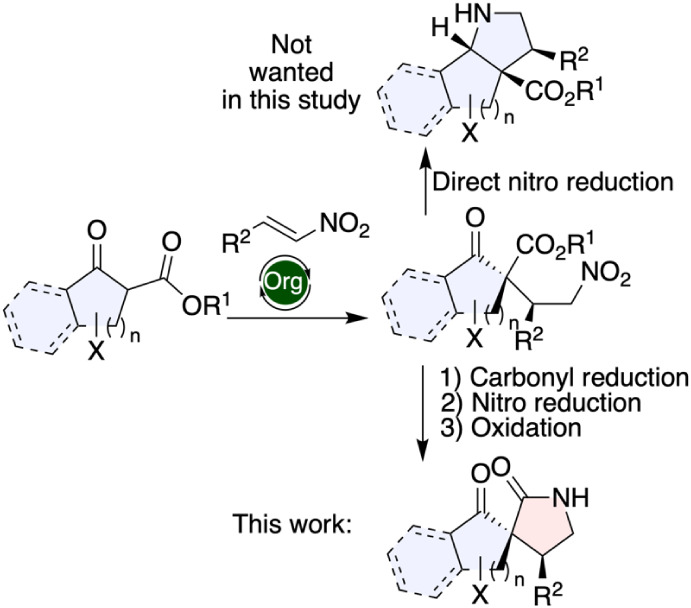
Strategy for the Synthesis of Spiro-2-pyrrolidinones

## Results and Discussion

Our group has developed different catalysts for the Michael addition of 1,3-dicarbonyl compounds to nitrostyrenes.^11^ Therefore, we carried out the Michael addition of ketoester **2** to nitroalkenes **1a**–**l** using the reported catalyst **C1** under optimized conditions. Products **3a–l** were obtained in good yields and selectivities, with the exception of substrates **3e** bearing a phenyl ring with an ortho-trifluoromethyl group and **3i** that contains a phenyl ring with two methoxy groups, which afforded lower yields—likely due to steric hindrance in the former case and decreased electrophilicity of the nitrostyrene in the latter. Additionally, the alkyl-substituted nitroalkene **3j** showed reduced yield and selectivity (Scheme 2).

**Scheme 2:**
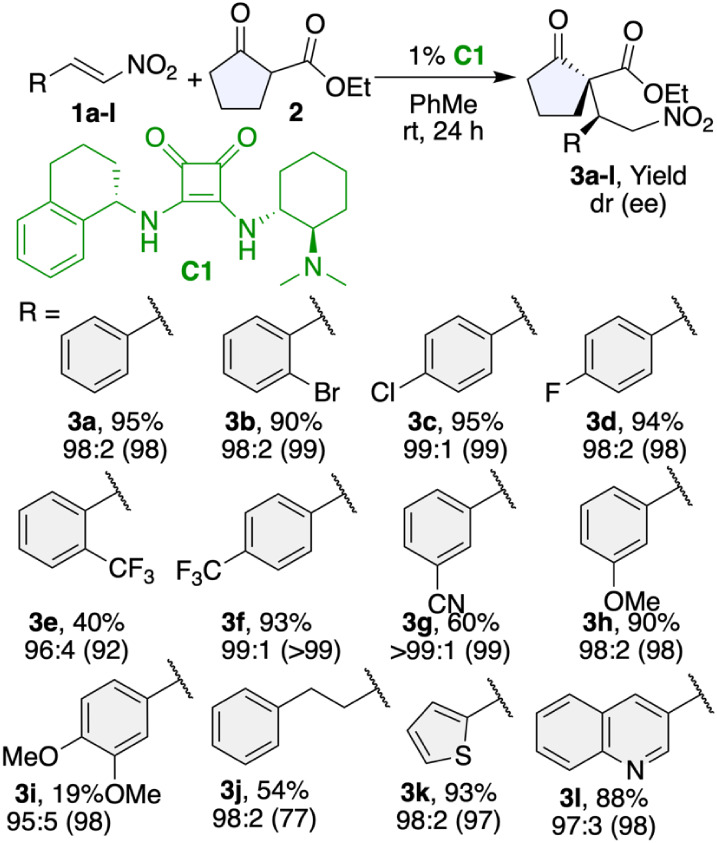
Michael Addition of Ketoester of Cyclopentanone **2** to Nitroalkenes **1**

We also studied different cyclic ketoesters of various ring sizes, benzo-fused derivatives such as indanone, as well as those containing heteroatoms^12^ within the ring, obtaining the Michael addition products with good yield and selectivity. We selected catalysts **C2** and **C3** for ketoesters **7**, **8**, and **9** because they provided higher enantioselectivities than catalyst **C1**, particularly in the case of the six-membered-ring ketoesters (Scheme 3).

**Scheme 3:**
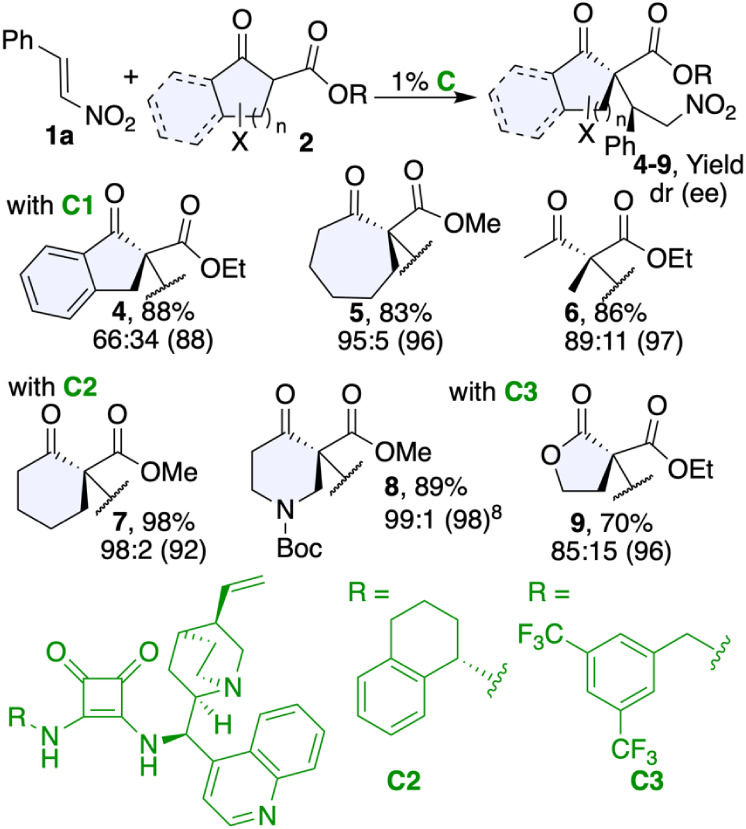
Michael Addition of Different Ketoesters to β-Nitrostyrene **1a**

According to the proposed mechanism,^13a^ the racemic ketoester is first deprotonated by the tertiary amine and subsequently stabilized through hydrogen bonds by the squaramide moiety. The protonated amine (ammonium) then activates the nitrostyrene through hydrogen bonding, bringing the nucleophile and electrophile into proximity and facilitating carbon–carbon bond formation. In our catalytic system, the orientation of the ketoester determines the diastereoselectivity of the reaction. The ketone oxygen is preferentially positioned on the side closest to the positively charged ammonium group, favoring electrostatic stabilization. Subsequently, the nitrostyrene is incorporated into the reactive complex through hydrogen bonding with the ammonium center. Additionally, complementary CH···π interactions between the aromatic rings help to fix the position of the nitrostyrene within the transition state, promoting stereoselective formation of the new carbon–carbon bond (Chart [Fig chart2]). The configuration was assigned by analogy to a previously reported product using a catalyst related to **C1**, bearing an *N*-benzyl group instead of an *N*-1-(1,2,3,4-tetrahydronaphthyl) substituent.^13a^ The assignment was further corroborated by comparison of its optical rotation with that of the corresponding enantiomer reported in the literature.^13b^

**2 chart2:**
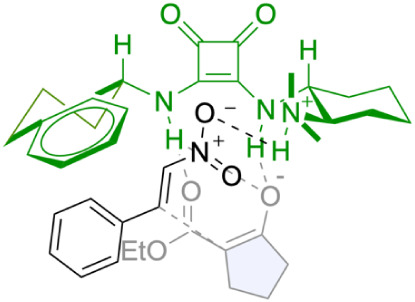
Proposed Transition State of the Stereoselective Michael Addition with **C1**

We next turned our attention to the synthesis of the spiro-2-pyrrolidinones. As noted above, it is necessary to suppress the electrophilic character of the ketone prior to reduction of the nitro group. Initially, we sought to carry out a diastereoselective reduction of the ketone in Michael adduct **3a**, thereby eliminating the need for the final oxidation step. However, the best diastereomeric ratio obtained was 85:15 (see Table S1 in the SI). We also explored protection of the ketone as an acetal, but found that direct reduction of the ketone followed by oxidation of the resulting alcohol after lactamization provided superior overall results. Accordingly, we adopted this latter strategy in which ketone reduction, nitro group reduction, and subsequent lactamization are performed sequentially in a one-pot procedure. Final oxidation of the alcohol afforded compounds **10a–l** and **11–15** with an average yield of 42% over the four-step sequence (Scheme 4). Notably, purification by column chromatography was required only for the final products, and among the individual transformations, only the lactamization step required reaction times of several hours (this step is responsible for the moderate yields), whereas the remaining steps were completed within an hour.

**Scheme 4:**
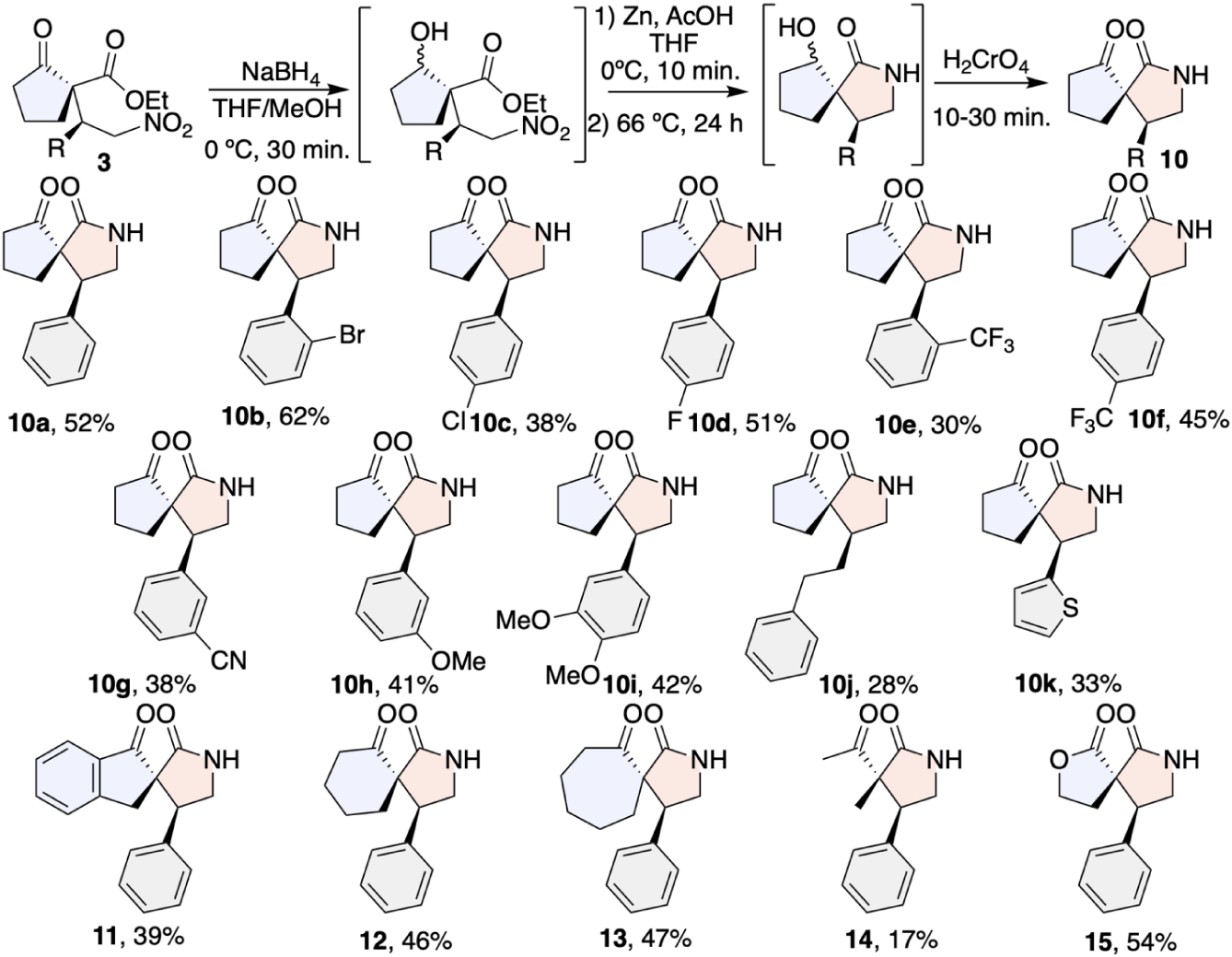
Synthesis of Spiro-2-pyrrolidinones

This procedure is incompatible with acid-sensitive protecting groups, such as the Boc group present in compound **8**. For this substrate and **3l**, we changed the reducing and oxidizing agents, obtaining spirolactams **15** and **10l** in good yield. Again, under this set of conditions, the ketone reduction, nitro group reduction, and subsequent lactamization can be carried out in a one-pot sequence with only one chromatographic purification (Scheme 5).

**Scheme 5:**
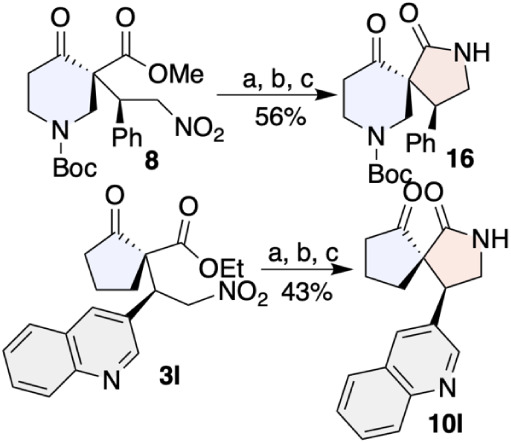
Synthesis of Spiro-2-pyrrolidinone **16** under Non-Acidic Conditions; (a) NaBH_4_, THF/MeOH 1:1, −5 °C, 10 min; (b) (i) NiCl_2_·6H_2_O, NaBH_4_, −5 °C, 10 min; (ii) 66 °C, 25 h; (c) IBX, DMSO, rt, 21 h

We scaled-up the reaction to 1 mmol for **10a** with the set of conditions shown in Schemes 2 and 4 obtaining similar results. We also carried out further transformations of spirolactam **10a** to demonstrate its potential for conversion into more complex products. Accordingly, bisamide **17** was obtained via a Beckmann rearrangement, compound **18** through a Fischer indole synthesis, and alcohol **19** through ketone reduction^14^ with good diastereoselectivity (Scheme 6).

**Scheme 6:**
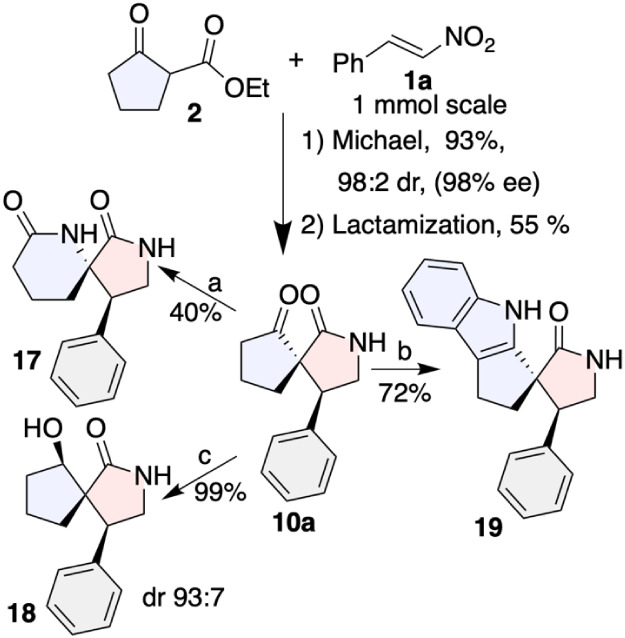
Scale-up Reaction for **10a** and Reactions for Derivatizing **10a**; (a) (i) HONH_2_·HCl, MeOH, 66 °C, 2 h; (ii) *p*-TsOH, DMAP, Py, 85 °C, 17 h. (b) H_2_NNHPh, PhMe, 1 h rt; (ii) *p*-TsOH, 80 °C, 4 h. (c) NaBH_4_, THF/MeOH 1:1, −20 °C, 30 min

A chemoinformatic study correlating the chemical diversity and bioactivity of lactams indicates that the spiro- and bridged-lactams have the lowest number of compounds studied and showed activity against specific targets.^15^ Therefore, having these compounds at hand we tested different biological activities.

Inflammation is a fundamental immune response that helps maintain homeostasis by protecting the body against tissue damage, pathogens, or even autoimmune reactions. Mediated by immune cells and inflammatory mediators, it promotes tissue repair and host defense. However, when inflammation becomes chronic or dysregulated, it contributes to the development of numerous diseases, including cardiovascular disorders, diabetes, cancer, autoimmune diseases, neurodegenerative conditions, and several mental health disorders. The development of new anti-inflammatory drugs remains relevant as current drugs are often associated with limited efficacy in some patients, and significant adverse effects with long-term use.^16a^ The development of selective cyclooxygenase-2 (COX-2) inhibitors remains a major focus in medicinal chemistry. Because COX-2 is upregulated during inflammation and mediates the production of pro-inflammatory prostaglandins, whereas COX-1 plays essential physiological roles, including gastric mucosal protection, platelet aggregation, and renal homeostasis. Therefore, selective inhibition of COX-2 by new scaffolds represents a promising strategy for developing safer anti-inflammatory agents with reduced gastrointestinal toxicity compared with conventional NSAIDs.^16b^

Before the anti-inflammatory study, we explored the cytotoxic activity against cancer cell lines, but no significant effect was found; moreover, a good result was that the blank assay in monkey kidney cells COS-7 also showed little to no toxicity for most of them (Tables S3 and S4 in the SI).

Then, we evaluated the anti-inflammatory properties of the spiro-2-pyrrolidinones^17^ using the mouse ear edema model induced by 12-*O*-tetradecanoylphorbol-13-acetate (TPA). Figure 1 shows the percentage of inhibition of the compounds studied. First, we found that *rac*-**10a** is less active than the pure enantiomer **10a**. Since one enantiomer is typically more biologically active (the eutomer) than its counterpart (the distomer), the reduced anti-inflammatory activity of the racemic compound may be attributed to the presence of only 50% of the active eutomer. This finding further underscores the importance of accessing homochiral compounds, which may exhibit superior biological activity compared with their racemic counterparts. Most of the changes in the aryl ring did not show better inhibition, only the methoxy substitution in **10h** and **10i** showed a similar result, and the trifluoromethyl-containing **10e** and **10f** also showed significant activity like celecoxib (Figure 1).

**Figure 1:**
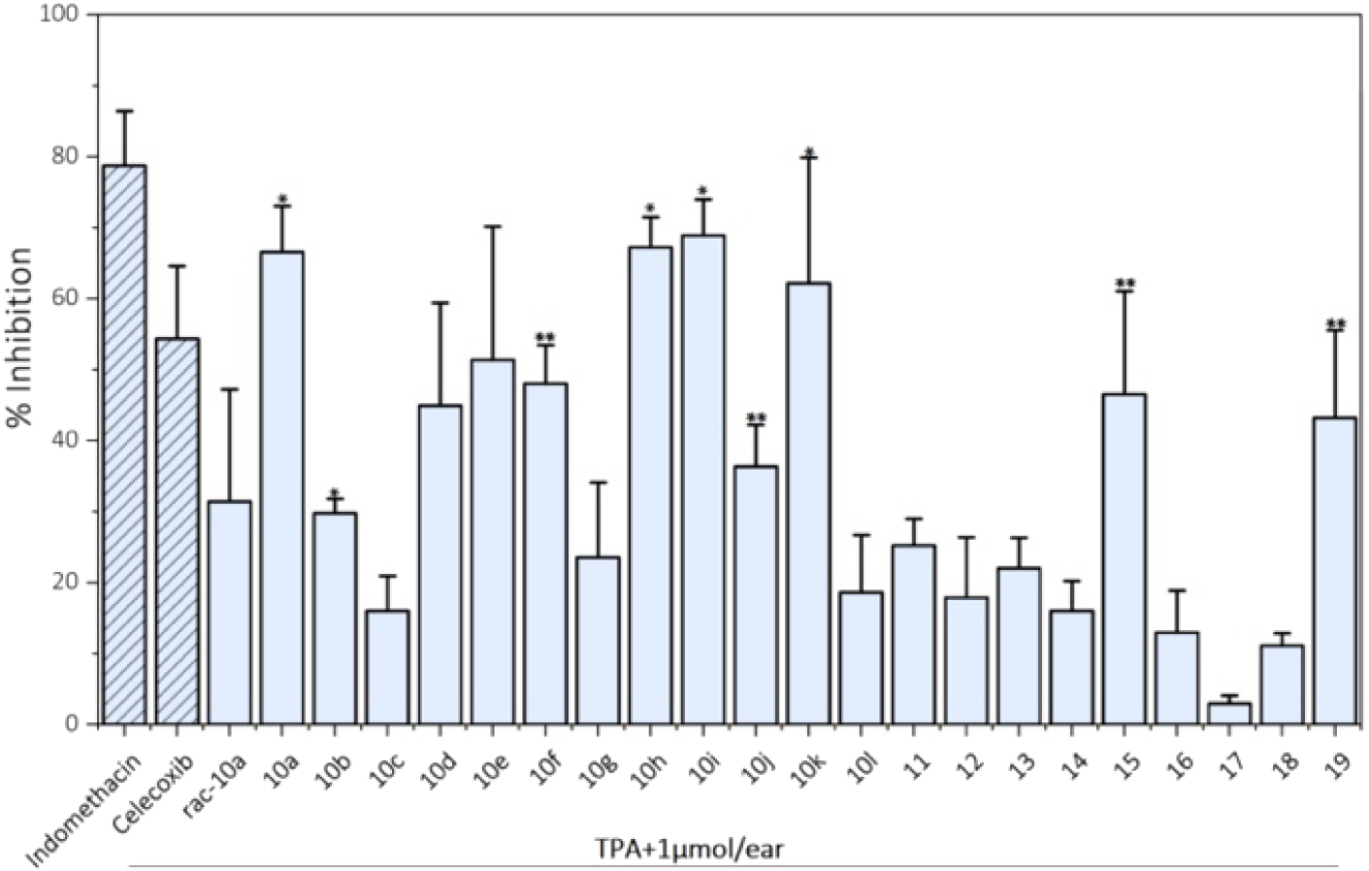
Effect of spirolactams (compounds **10–19**) on TPA-induced ear edema in mice. Results are presented as the mean ± SEM from three repetitions. The percentage of inhibition was calculated based on the difference in weight between untreated ear biopsy and biopsy with TPA, either alone or in combination with the indicated control (indomethacin and celecoxib) or spirocycle. Statistical significance was evaluated relative to the TPA-only group using one-way ANOVA followed by Dunnett’s post hoc test (*P ≤ 0.05, **P ≤ 0.01 inhibition of edema in the ear).

The tested compounds significantly reduced TPA-induced ear edema, indicating a pronounced anti-inflammatory effect. To study if the anti-inflammatory effect was associated with decreased neutrophil infiltration, we quantified myeloperoxidase (MPO) levels in the same ear tissues. As shown in Figure 2, compound treatment at a dose of 1 mmol/ear produced a reduction in MPO content, suggesting that the observed antiedematous effect is, at least partially, mediated by inhibition of neutrophil recruitment possibly due to the interference of mediating factors such as IL-1β or COX-2.

**Figure 2:**
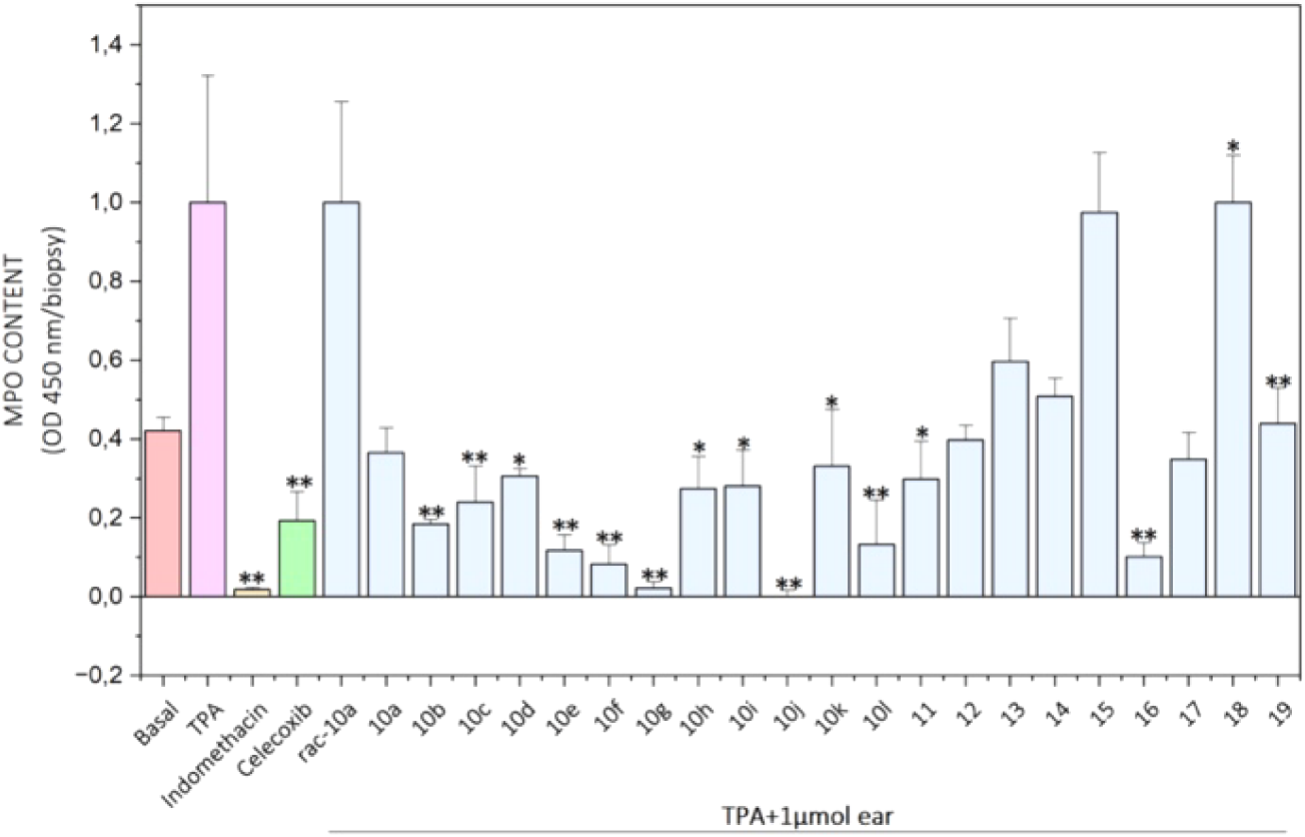
Effect of topical dose (1 μmol/ear) of compounds **10**–**19** on MPO content. It was measured by colorimetric assay (OD450 nm) in ear edema homogenates. Results were normalized with respect to their corresponding control group and are presented as mean ± SEM from three repetitions per group. Statistical significance was determined relative to the TPA-only group (positive control) using one-way ANOVA followed by Dunnett’s post-hoc test (*P ≤ 0.05, **P ≤ 0.01).

Finally, inflammatory effects can arise by COX-2 that plays a central role in the generation of pro-inflammatory prostaglandins that contribute to vasodilation, increased vascular permeability, and leukocyte recruitment. Therefore, its inhibition may lead to a reduction in inflammation. However, the inhibition of COX-1 causes adverse effects such as gastric irritation, increased bleeding risk, and impaired kidney function. Therefore, we studied the inhibition of COX isoforms to see if there could be a selective inhibition. The *in vitro* control experiments of inhibited enzymes we used indomethacin for COX-1 and celecoxib for COX-2. We tested **10a**, **10i** and **10f** to compare the unsubstituted aryl and with two different groups that presented good anti-inflammatory activity. We found that **10a** and **10f** inhibit considerably more COX-2 than COX-1 (Figure 3).

**Figure 3:**
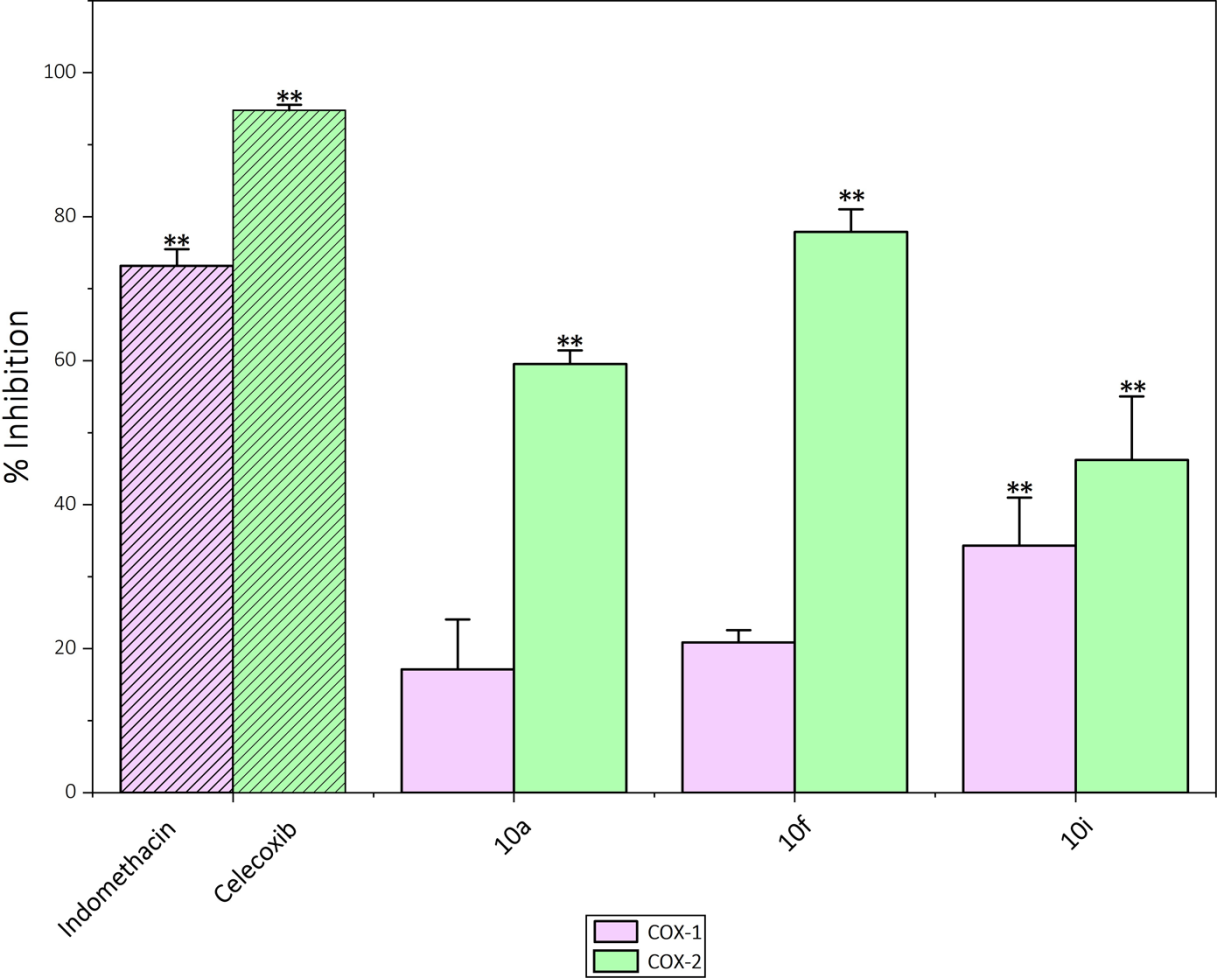
Effect of the samples on prostaglandin production by cyclooxygenase-1 and -2 (COX-1 and COX-2) at 50 μM (See Table S5 for experiments at different concentrations). Data are presented as mean ± SEM of three wells.

## Conclusions

We obtained a series of chiral spiro-2-pyrrolidinones bearing different substituents at the C-4 position of the lactam ring by using different β-nitrostyrenes. Also, depending on the starting ketoester, different ring systems can be incorporated to the spiro-fused pyrrolidinone. We also described nitro group reduction and lactamization under both acidic and basic conditions, which can be selected depending on the substrate. Moreover, the studied compounds exhibited anti-inflammatory activity and reduced MPO levels in inflamed tissue. Three compounds were further evaluated and showed greater inhibition of COX-2 than COX-1, suggesting that chirality and the specific spatial arrangement of substituents are critical for this selectivity.

## Supplementary Material



## Notes

Experimental procedure, physical data, ^1^H NMR spectra, and ^13^C NMR spectra of compounds **3**–**19**; cytotoxicity studies, diastereoselective reduction of **3a** and **18**, and NOESY of **18** (PDF)
