# Supplementary material for: Synthesis of Chiral Spiro-2-pyrrolidinones with Anti-Inflammatory Properties
Source: ACS Omega. 2026 Jun 26;11(27):40785–91. doi: 10.1021/acsomega.6c04526 (PMC13382671; doi:10.1021/acsomega.6c04526)
Supplement: Supplementary file 1 [file ao6c04526_si_001.pdf]

Supporting information

Synthesis of chiral spiro 2-pyrrolidinones with anti-inflammatory properties

Karla D. Torres-Muñoz,[a] Diego A. Cruz-Aguilar,[a] Antonio Nieto-Camacho[a] and Marcos Hernández-Rodríguez [a]*

[a]Universidad Nacional Autónoma de México, Instituto de Química

Circuito Exterior, Ciudad Universitaria, Alc. Coyoacán, Ciudad de México, 04510, México

Email: marcoshr@unam.mx

Table of contents

Table S1. Diastereoselective reduction of Michael adduct **3a**. 2

Table S2. Diastereoselective reduction of lactam **10a**. 3

Figure 1. NOESY experiment of alcohol **18**. 3

Table S3. Percentage of cytotoxicity in monkey kidney cell line non-cancerous (COS-7) at 25 μM. 4

Table S4. Percentage of cytotoxicity at 25 μM in different cell lines. 5

Table S5. Effect of the 2-pyrrolidones on prostaglandin production by the Cyclooxygenase-1 and 2 enzymes. 6

1. General procedure for the Michael Addition (**GP1**) 7
2. General procedure 2 for the synthesis of spiro-2-pyrrolidinones (**GP2**) 15
3. General procedure 3 for the synthesis of spiro-2-pyrrolidinones (**GP3**). 21
4. Copies of NMR spectra for the products 25
5. Chiral stationary phase HPLC chromatograms 64
6. **References** 82

Table S1. Diastereoselective reduction of Michael adduct **3a**.

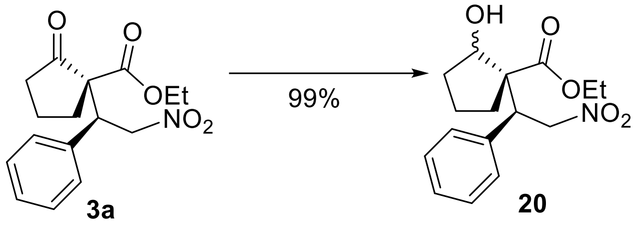

Exp.	Reductant	Solvent (Conc. M)	T (ºC)	Time	d.r.
**1**	$NaBH_4$	THF/MeOH 1:1 (0.5)	0	10 min.	38:62
**2**	$NaBH_4$	$EtOH/CH_2Cl_2$ (0.156)	0	15 min.	42:58
**3**	$NaBH_4$	$EtOH/CH_2Cl_2$ (0.156)	-78	4 h	25:75
**4**	$NaBH_4$	$EtOH/CH_2Cl_2$ (0.156)	-20	4 h	37:63
**5**	$NaBH_4$	*t*-$BuOH/CH_2Cl_2$ (0.156)	0	15 min.	32:68
**6**	$NaBH_4$, $CeCl_3{\cdot}7H_2O$	$EtOH/CH_2Cl_2$ (0.156)	-78	4 h	39:61
**7**	$LiBH_4$	$EtOH/CH_2Cl_2$ (0.156)	0	10 min.	43:57
**8**	**$LiBH_4$, $CeCl_3{\cdot}7H_2O$**	**$EtOH/CH_2Cl_2$ (0.156)**	**-78**	**10min.**	**14:86**
**9**	NMe_4BH_4	$EtOH/CH_2Cl_2$	-78	2 h	66.34
**10**	NMe_4BH_4	$EtOH/CH_2Cl_2$	0	10 min.	52:41
**11**	$LiBH_4$, $CeCl_3{\cdot}7H_2O$	*t*-$BuOH/CH_2Cl_2$	-78	1 h	70:30
**12**	$LiBH_4$, $CeCl_3{\cdot}7H_2O$	Isopropanol/CH_2Cl_2	-78	4 h	59:41
**13**	NMe_4BH_4	CH_2Cl_2	-78	1 h	62:38
**14**	$LiBHEt_3$	THF (0.2 M)	-78	4 h	29:71
**15**	L-Selectride	THF (0.2 M)	-78	3 h	35:65

Table S2. Diastereoselective reduction of lactam **10a.**

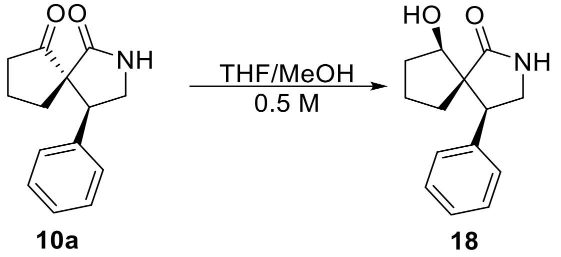

Exp.	Reducing agent	T (ºC)	Time	d.r.
**1**	$NaBH_4$	-40	2 h	58:42
**2**	**$NaBH_4$**	**-20**	**30 min.**	**94:6**
**3**	$NaBH_4$	0	10 min.	89:11
**4**	$NaBH_4$	T.A.	10 min.	90:10
**5**	$LiBH_4$	0	10 min.	87:13
**6**	$LiBH_4$, $CeCl_3\cdot7H_2O$	0	10 min.	74:26
**7***	$LiBH_4$, $CeCl_3\cdot7H_2O$	-78	6 h	54:46
**8**	NMe_4BH_4	0	10 min.	86:14

*Reaction in DCM/EtOH 2:1 (0.15 M).

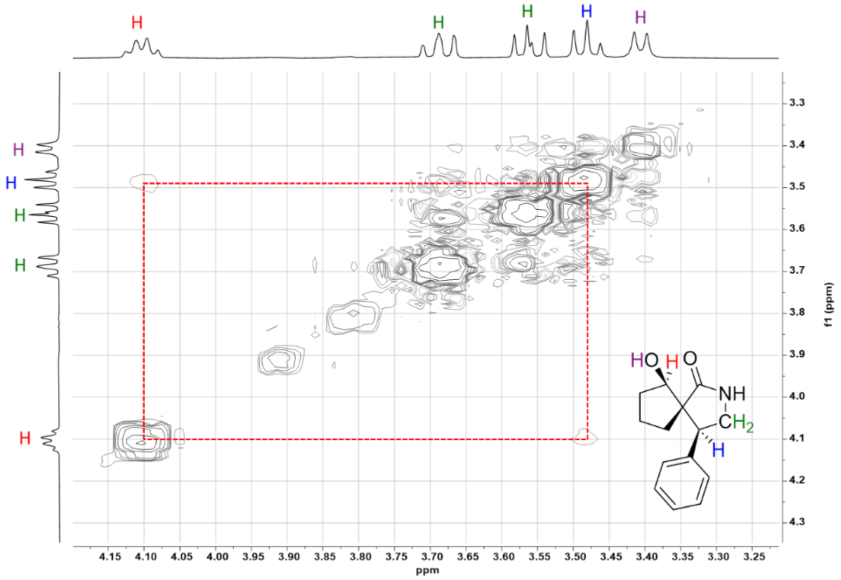

Figure 1. NOESY experiment of alcohol **18**.

Table S3. Percentage of cytotoxicity in monkey kidney cell line non-cancerous (COS-7) at 25 μM.

Compound	COS-7
rac-**10a**	15.8
**10a**	9.4
**10b**	NC
**10c**	10.7
**10d**	10.6
**10e**	10.3
**10f**	100
**10g**	6.7
**10h**	10.6
**10i**	0.31
**10j**	17.9
**10k**	14.8
**10l**	31.8
**11**	13.2
**12**	NC
**13**	13.2
**14**	0.8
**15**	10.4
**16**	0.5
**17**	21.2
**18**	14.3
**19**	28.5

NC: Non-cytotoxic.

Table S4. Percentage of cytotoxicity at 25 μM in different cell lines.

U261: Central nervous system glia, **PC-3:** prostate, **K562:** leukemia, **HTC-15:** colon, **MCF-7:** breast, **SKLU:** lung.

Compound	U251	PC-3	K562	HCT-15	MCF-7	SKLU-1
rac-**10a**	NC	0.8	NC	6.4	21.4	15.1
**10a**	NC	NC	5.3	7.5	13.7	18.7
**10b**	5.7	NC	9.8	5.7	NC	NC
**10c**	3.7	NC	9.0	NC	3.8	17.8
**10d**	NC	NC	NC	5.8	17.4	8.5
**10e**	NC	NC	NC	NC	16.8	9.9
**10g**	NC	NC	9.6	NC	7.4	14.1
**10h**	NC	NC	NC	4.6	21.1	14.9
**10i**	NC	NC	0.1	NC	3.5	4.8
**10k**	NC	NC	NC	7.5	9.2	10.0
**12**	NC	NC	2.8	NC	NC	NC
**18**	16.4	NC	NC	14.0	7.1	16.0
**19**	20.3	20.2	NC	12.9	26.3	21.5

Table S5. Effect of the 2-pyrrolidones on prostaglandin production by the Cyclooxygenase-1 and 2 enzymes.

COX-1: Cyclooxygenase-1, **COX-2:** Cyclooxygenase-2, **PG:** Prostaglandin

**Treatment**	**Concentration (µM)**	**COX-1**		**COX-2**	
		**PG (ng/mL)**	**Inhibition (%)**	**PG (ng/mL)**	**Inhibition (%)**
Control (DMSO)	-	32.22±1.31	0.00±4.08	38.97±4.89	0.00±12.55
**10a**	1	29.88±2.71	7.26±8.42	29.71±3.12	23.76±8.01
	10	26.96±1.37	16.30±4.26	22.74±1.46**	41.64±3.76**
	50	26.70±2.23	17.13±6.92	15.77±0.74**	59.54±1.90**
**10f**	1	27.05±1.59	16.03±4.93	24.31±2.16**	37.61±5.54**
	10	27.97±0.43	13.17±1.32	13.38±0.88**	65.67±2.26**
	50	25.49±0.54	20.88±1.68	8.61±1.22**	77.89±3.14**
**10i**	1	31.07±3.05	3.55±9.47	31.94±4.45	18.03±11.42
	10	28.73±0.145	10.81±0.43	29.30±2.14	24.80±5.49
	50	21.16±2.14**	34.33±6.64**	20.97±3.44**	46.20±8.84**
Indomethacin	50	8.64±0.75**	73.18±2.31**	-	-
Celecoxib	50	-	-	2.03±0.29**	94.78±0.75**

The data represent the mean of three wells ± standard error of the mean (SEM). The results were analyzed using Dunnett's test. Values of $p \leq 0.05$ (*) and $p \leq 0.01$ (**) were considered statistically significant compared with the control group.

1. General procedure for the Michael Addition (**GP1**)

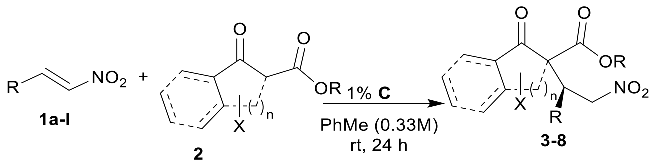

In a 7 mL screw-cap vial with stirring bar was dissolved the corresponding nitrostyrene (0.5 mmol, 1 equiv.) and the bifunctional organocatalyst (0.005 mmol, 0.01 equiv.) in toluene (0.33 M, 1.52 mL). Subsequently, the corresponding ketoester (1 mmol, 2 equiv.) was added, and the reaction mixture was stirred at room temperature for 24 h. After completion, the reaction was concentrated under reduced pressure and purified by flash column chromatography.

Ethyl (*R*)-1-((*S*)-2-nitro-1-phenylethyl)-2-oxocyclopentane-1-carboxylate (**3a**)[1]

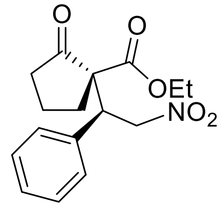

Prepared according to **GP1** using β-nitrostyrene and 2-ethoxycarbonylcyclopentanone as starting materials. The product was purified by flash column chromatography (silica gel, hexane/EtOAc 95:5-8:2). Viscous colorless liquid (145.0 mg, 95% yield), R_f = 0.3 (hexane/EtOAc 85:15). $[\alpha]_D^{25}$ = -29.1 (*c* 0.82, $CHCl_3$), for the enantiomer (98% ee, d.r. = 97:3): $[\alpha]_D^{25}$ = +27.0 (*c* 0.44, CH_2Cl_2).[2] d.r. = 98:2. **^{1}H NMR ($CDCl_3$, 400 MHz)** δ = 7.23-7.24 (m, 5H), 5.17 (dd, *J* = 13.6, 3.9 Hz, 1H), 5.01 (dd, *J* = 13.6, 10.9 Hz, 1H), 4.25-4.17 (m, 2H), 4.1 (dd, *J* = 10.9, 3.9 Hz, 1H), 2. 41-2.31 (m, 2H), 2.06-1.88 (m, 3H), 1.88-1.76 (m, 1H), 1.3 (t, *J* = 1.3 Hz, 3H). **^{13}C{^{1}H} NMR ($CDCl_3$, 100 MHz)** δ = 212.4, 169.4, 135.5, 129.4, 128.9, 128.4, 76.6, 62.5, 62.3, 46.3, 38.0, 31.3, 19.4, 14.1. **HRMS (EI/TOF):** *m/z* = Calculated for $C_{16}H_{19}NO_5$: 305.1263, found: 305.1273. **HPLC:** (Chiralpak IC-3, hexane/ethanol 85:15, 0.6 mL/min., 220 nm): $t_{major\ enant.}$ = 10.11 min., $t_{minor\ enant.}$ = 8.19 min., 98% ee.

Ethyl (*R*)-1-((*R*)-1-(2-bromophenyl)-2-nitroethyl)-2-oxocyclopentane-1-carboxylate (**3b**)

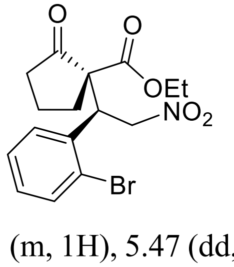

Prepared according to **GP1** using *trans*-2-bromo-β-nitrostyrene and 2-ethoxycarbonylcyclopentanone as starting materials. The product was purified by flash column chromatography (silica gel, hexane/EtOAc 95:5-7:3). Viscous colorless liquid (171.6 mg, 90% yield), R_f = 0.37 (hexane/EtOAc 8:2). $[\alpha]_D^{25}$ = +27.6 (*c* 0.79, CH_2Cl_2), d.r. = 98:2. **^{1}H NMR ($CDCl_3$, 400 MHz)** δ = 7.61-7.53 (m, 2H), 7.34-7.27 (m, 1H), 7.18-7.09 (m, 1H), 5.47 (dd, *J* = 13.8, 3.6 Hz, 1H), 5.06 (dd, *J* = 13.8, 10.6 Hz, 3H), 4.51 (dd, *J* = 10.6, 3.6 Hz, 1H), 4.20 (q, 2H, *J* = 7.1 Hz, 2H), 2.57 (m, 2H), 2.28-2.17 (m, 1H), 2.15-2.05 (m, 1H), 2.01-1.88 (m, 2H), 1.26 (t, *J* = 7.1 Hz, 3H). **^{13}C{^{1}H} NMR ($CDCl_3$, 100 MHz)** δ = 212.7, 169.5, 136.7, 133.6, 129.7, 129.2, 128.3, 126.88, 77.1, 62.3, 62.2, 44.0, 37.9, 33.2,

19.4, 14.1. **HRMS (DART/TOF):** m/z $[M+H]^+$: Calculated for $C_{16}H_{18}BrNO_5$: 384.0481, found: 384.0430. **HPLC:** (Chiralpak IC-3, hexane/ethanol 85:15, 0.6 mL/min, 220 nm): $t_{major\ enant.}$ = 9.17 min., $t_{minor\ enant.}$ = 8.12 min, 99% ee.

Ethyl (*R*)-1-((*S*)-1-(4-chlorophenyl)-2-nitroethyl)-2-oxocyclopentane-1-carboxylate (**3c**)

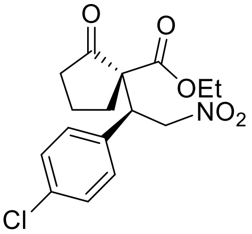

It was prepared according to the general procedure (**GP1**), *trans*-4-chloro-β-nitrostyrene as nitrostyrene and 2-ethoxycarbonylcyclopentanone as starting materials. The product was purified by flash column chromatography (silica gel, hexane/EtOAc 95:5-7:3). Viscous yellow liquid (161.4 mg, 95% yield), R_f = 0.37 (hexane/EtOAc 8:2). $[\alpha]_D^{25}$ = -34.5 (*c* 0.53, CH_2Cl_2), d.r. = 99:1. **^{1}H NMR ($CDCl_3$, 400 MHz)** δ = 7.32-7.26 (m, 2H), 7.26-7.20 (m, 2H), 5.16 (dd, *J* = 13.7, 3.8 Hz, 1H), 4.97 (dd, *J* = 13.7, 11.1 Hz, 1H), 4.26-4.16 (m, 2H), 4.03 (dd, *J* = 11.1, 3.8 Hz, 1H), 2.44-2.31 (m, 2H), 2.14-2.01 (m, 1H), 2.00-1.80 (m, 3H), 1.27 (t, *J* = 7.1 Hz, 3H). **^{13}C{^{1}H} NMR ($CDCl_3$, 100 MHz)** δ = 212.3, 169.3, 134.4, 134.1, 130.9, 129.1, 76.4, 62.4, 62.4, 45.7, 37.9, 31.5, 19.4, 14.0. **HRMS (EI/TOF):** *m/z* = Calculated for $C_{16}H_{18}ClNO_5$ = 339.0874, found: 339.0869. **HPLC:** (Chiralpak IC-3, hexane/ethanol 85:15, 0.6 mL/min, 220 nm): $t_{major\ enant.}$ = 8.73 min., $t_{minor\ enant.}$ = 7.28 min., 99% ee.

Ethyl (*R*)-1-((*S*)-1-(4-fluorophenyl)-2-nitroethyl)-2-oxocyclopentane-1-carboxylate (**3d**)

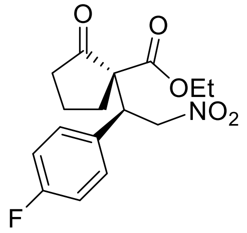

Prepared according to **GP1** using *trans*-4-fluoro-β-nitrostyrene and 2-ethoxycarbonylcyclopentanone as starting materials. The product was purified by flash column chromatography (silica gel, hexane/EtOAc 9:1-7:3). Viscous yellow liquid (151.9 mg, 94% yield), R_f = 0.33 (hexane/EtOAc 8:2). $[\alpha]_D^{25}$ = -21.4 (*c* 0.48, $CHCl_3$), d.r. = 98:2. **^{1}H NMR ($CDCl_3$, 400 MHz)** δ = 7.31-7.21 (m, 2H), 7.09-6.90 (m, 2H), 5.15 (dd, *J* = 13.6, 3.7Hz, 1H), 4.97 (dd, *J* = 11.4, 13.3 Hz, 1H), 4.26-4.14 (m, 2H), 4.05 (dd, *J* = 11.1, 3.8Hz, 1H), 2.46-2.28 (m, 2H), 2.13-2.00 (m, 1H), 1.98-1.79 (m, 3H), 1.32-1.20 (m, 3H). **^{13}C{^{1}H} NMR ($CDCl_3$, 100 MHz)** δ = 212.3, 169.4, 169.3, 162.6 (d, *J* = 247.9 Hz), 131.2 (d, *J* = 8.2 Hz), 115.8 (d, *J* = 21.4 Hz), 76.6, 62.5, 62.4, 45.6, 37.9, 31.5, 19.4, 14.1. **HRMS (EI/TOF):** *m/z* = Calculated for $C_{16}H_{18}FNO_5$ = 323.1169, found: 323.1161. **HPLC:** (Chiralpak IC-3, hexane/ethanol 85:15, 0.6 mL/min, 220 nm): $t_{major\ enant.}$ = 8.69 min., $t_{minor\ enant.}$ = 7.26 min., 98% ee.

Ethyl (*R*)-1-((*S*)-2-nitro-1-(2-(trifluoromethyl)phenyl)ethyl)-2-oxocyclopentane-1-carboxylate (**3e**)

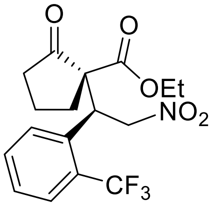

It was prepared according to the general procedure (**GP1**), *trans*-2-trifluoromethyl-β-nitrostyrene and 2-ethoxycarbonylcyclopentanone as starting materials. The product was purified by flash column chromatography (silica gel, hexane/EtOAc 95:5). Viscous colorless liquid (74.67 mg, 40% yield), R_f = 0.33 (hexane/EtOAc 8:2). $[\alpha]_D^{25}$ = -22.1 (*c* 0.48, $CHCl_3$), d.r. = 98:2. **^{1}H NMR ($CDCl_3$, 400 MHz)** δ = 7.87 (d, *J* = 7.9 Hz, 1H), 7.65 (dd, *J* = 7.9, 1.5 Hz, 1H), 7.56 (t, *J* = 7.7 Hz, 1H), 7.40 (t, *J* = 7.7 Hz, 1H), 5.35 (dd, *J* = 12.1, 4.9 Hz, 1H), 4.85 (dd, *J* = 12.1, 9.0 Hz, 1H), 4.21 (dd, *J* = 9.0, 4.9 Hz, 1H), 4.13 (q, *J* = 7.1 Hz, 2H), 2.57-2.47 (m, 1H), 2.44-2.33 (m, 1H), 2.22-2.12 (m, 1H), 2.00-1.91 (m, 1H), 1.87-1.73 (m, 1H), 1.18 (t, *J* = 7.1 Hz, 1H). **^{13}C{^{1}H} NMR ($CDCl_3$, 100 MHz)** δ = 212.7, 170.1, 136.4, 132.7, 129.9, 129.7 (q, *J* = 29.3 Hz), 128.3, 126.7 (q, *J* = 6.0 Hz), 124.1 (q, *J* = 274.2 Hz), 78.1, 62.3, 42.0, 41.0, 37.6, 34.4, 19.1, 13.9. **HRMS (DART/TOF):** m/z $[M+H]^+$: Calculated for $C_{17}H_{18}F_3NO_5$ = 374.1210, found: 374.1206. **HPLC:** (Chiralpak AD-H 250, hexane/isopropanol 90:10, 1.0 mL/min, 220 nm): major diastereomer: $t_{major\ enant.}$ = 8.38 min., $t_{minor\ enant.}$ = 10.29 min., 96% ee; minor diastereomer: only one enantiomer, t = 8.88 min.

Ethyl (*R*)-1-((*S*)-2-nitro-1-(4-(trifluoromethyl)phenyl)ethyl)-2-oxocyclopentane-1-carboxylate (**3f**)

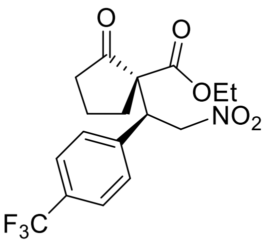

Prepared according to **GP1** using *trans*-4-trifluoromethyl-β-nitrostyrene and 2-ethoxycarbonylcyclopentanone as starting materials. The product was purified by flash column chromatography (silica gel, hexane/EtOAc 95:5-8:2). Viscous yellow liquid (173.6 mg, 93% yield), R_f = 0.3 (hexane/EtOAc 9:1). $[\alpha]_D^{25}$ = -26.1 (*c* 0.51, CH_2Cl_2), d.r. = 99:1. **^{1}H NMR ($CDCl_3$, 400 MHz)** δ = 7.58 (d, *J* = 8.2 Hz, 2H), 7.45 (d, *J* = 8.2 Hz, 2H), 5.22 (dd, *J* = 13.8, 3.7 Hz, 1H), 5.04 (dd, *J* = 13.8, 11.1 Hz, 1H), 4.26-4.17 (m, 2H), 4.09 (dd, *J* = 11.1, 3.7 Hz, 1H). 2.47-2.33 (m, 2H), 2.15-2.05 (m, 1H), 2.01-1.82 (m, 3H), 1.29-1.21 (m, 3H). **^{13}C{^{1}H} NMR ($CDCl_3$, 100 MHz)** δ = 212.1, 169.3, 139.8, 130.6 (q, *J* = 32.8 Hz), 130.0, 125.8 (q, *J* = 3.8 Hz) 123.9 (q, *J* = 272.2 Hz), 76.2, 62.5, 62.2, 46.0, 37.8, 31.8, 19.4, 14.0. **HRMS (EI/TOF):** *m/z*: Calculated for $C_{17}H_{18}F_3NO_5$: 373.1137, found: 373.1212. **HPLC:** (Chiralpak AD-H 250, hexane/isopropanol 90:10, 0.5 mL/min, 220 nm): $t_{major\ enant.}$ = 15.8 min., $t_{minor\ enant.}$ = 14.6 min., >99% ee.

Ethyl (*R*)-1-((*S*)-1-(3-cyanophenyl)-2-nitroethyl)-2-oxocyclopentane-1-carboxylate (**3g**)

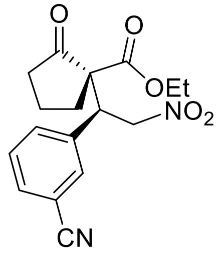

Prepared according to **GP1** using *trans*-3-cyano-β-nitrostyrene and 2-ethoxycarbonylcyclopentanone as starting materials. This product was purified by flash column chromatography (silica gel, hexane/EtOAc 8:2). Viscous yellow liquid (99.1 mg, 60% yield), R_f = 0.23 (hexane/EtOAc). $[\alpha]_D^{25}$ = -14.9 (*c* 1.0, CH_2Cl_2), d.r. = >99:1. **^{1}H NMR ($CDCl_3$, 400 MHz)** δ = 7.67-7.64 (m, 1H), 7.62-7.56 (m, 2H), 7.48-7.41 (m, 1H), 5.22 (dd, *J* = 14.0, 3.6 Hz, 1H), 5.03 (dd, *J* = 14.0, 11.1 Hz, 1H), 4.29-4.15 (m, 2H), 4.01 (dd, *J* = 11.1, 3.6 Hz, 1H), 2.50-2.40 (m, 1H), 2.38-2.27 (m, 1H), 2.22-2.07 (m, 1H), 2.03-1.80 (m, 3H), 1.27 (t, *J* = 7.1 Hz, 3H). **$^{13}C\{^1H\}$ NMR ($CDCl_3$, 100 MHz)** δ = 212.0, 169.3, 137.6, 134.3, 133.0, 132.1, 129.8, 118.3, 113.2, 76.1, 62.6, 62.1, 45.9, 37.8, 32.3, 19.4, 14.0. **HRMS (EI/TOF):** *m/z* = Calculated for $C_{17}H_{18}N_2O_5$ = 330.1216. **HPLC:** (Chiralpak IC-3, hexane/ethanol 85:15, 0.6 mL/min, 220 nm): $t_{major\ enant.}$ = 23.14 min., $t_{minor\ enant.}$ = 16.78., 99% ee.

Ethyl (*R*)-1-((*S*)-1-(3-methoxyphenyl)-2-nitroethyl)-2-oxocyclopentane-1-carboxylate (**3h**)

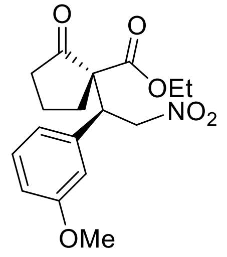

Prepared according to **GP1** using *trans*-3-methoxy-β-nitrostyrene and 2-ethoxycarbonylcyclopentanone as starting materials. This product was purified by flash column chromatography (silica gel, hexane/EtOAc 8:2-6:4). Viscous yellow liquid (150.91 mg, 90% yield), R_f = 0.3 (hexane/EtOAc 7:3). $[\alpha]_D^{25}$ = -28.2 (*c* 0.50, CH_2Cl_2), d.r. = 98:2. **^{1}H NMR ($CDCl_3$, 400 MHz)** δ = 7.26-7.17 (m, 1H), 6.87-6.75 (m, 3H), 5.15 (dd, *J* = 13.6, 3.8 Hz, 1H), 5.00 (dd, *J* = 13.6, 10.9 Hz, 1H), 4.21 (qd, *J* = 7.1, 1.7 Hz, 2H), 4.04 (dd, *J* = 10.9, 3.8 Hz, 1H), 3.78 (s, 3H), 2.42-2.30 (m, 2H), 2.18-1.90 (m, 3H), 1.89-1.79 (m, 1H), 1.28 (t, *J* = 7.1 Hz, 3H). **$^{13}C\{^1H\}$ NMR ($CDCl_3$, 100 MHz)** δ = 212.4, 169.4, 159.8, 137.1, 129.8, 121.5, 115.5, 113.5, 76.6, 62.5, 62.3, 55.3, 46.3, 38.0, 31.4, 19.4, 14.1. **HRMS (EI/TOF):** *m/z*: Calculated for $C_{17}H_{21}NO_6$: 335.1369, found: 335.1360. **HPLC:** (Chiralpak IC-3, hexane/ethanol 85:15, 0.6 mL/min, 220 nm): $t_{major\ enant.}$ = 10.98 min., $t_{minor\ enant.}$ = 9.19 min., 98% ee.

Ethyl (*R*)-1-((*S*)-1-(3,4-dimethoxyphenyl)-2-nitroethyl)-2-oxocyclopentane-1-carboxylate (**3i**)

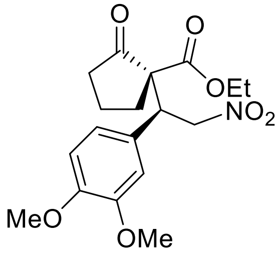

It was prepared according to the general procedure (**GP1**), *trans*-3,4-dimethoxy-β-nitrostyrene and 2-ethoxycarbonylcyclopentanone as starting materials. This product was purified by flash column chromatography (silica gel, hexane/EtOAc 8:2-6:4). Pale yellow solid (34.71 mg, 19% yield), R_f = 0.3 (hexane/EtOAc 7:3), m.p. = 83-85ºC, $[\alpha]_D^{25}$ = -28.2 (*c* 0.50, CH_2Cl_2), d.r. = 95:5. **^{1}H NMR ($CDCl_3$, 400 MHz)** δ = 6.84-6.75 (m, 3H), 5.09 (dd, *J* = 13.4, 3.9 Hz, 1H), 4.97 (dd, *J* = 13.4, 11.0 Hz, 1H), 4.25-4.16 (m, 2H), 4.05 (dd, *J* = 11.0, 3.9 H, 1H), 3.86 (s, 3H), 3.85

(s, 3H), 2.43-2.30 (m, 2H), 2.09-1.90 (m, 3H), 1.87-1.77 (m, 1H), 1.27 (t, *J* = 7.1 Hz, 3H). **$^{13}C\{^1H\}$ NMR ($CDCl_3$, 100 MHz)** δ = 212.6, 169.6, 149.0, 149.0, 127.7, 121.4, 112.9, 111.1, 76.7, 62.7, 62.3, 56.0, 55.9, 46.0, 38.0, 31.2, 19.5, 14.1. **HRMS (EI/TOF):** *m/z*: Calculated for $C_{18}H_{23}NO_7$: 365.1475, found: 365.1486. **HPLC:** (Chiralpak IC-3, hexane/ethanol 85:15, 0.6 mL/min, 220 nm): $t_{major\ enant.}$ = 25.20 min., $t_{minor\ enant.}$ = 24.12., 98% ee.

Ethyl (*R*)-1-((*S*)-1-nitro-4-phenylbutan-2-yl)-2-oxocyclopentane-1-carboxylate (**3j**)

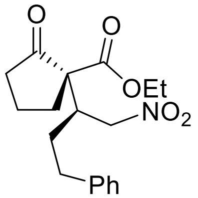

It was prepared according to the general procedure (**GP1**), (*E*)-(4-nitrobut-3-en-1-yl)benzene as nitrostyrene and 2-ethoxycarbonylcyclopentanone as starting materials. This product was purified by flash column chromatography (silica gel, hexane/EtOAc 9:1). Viscous yellow liquid (90.0 mg, 54% yield), R_f = 0.3 (hexane/EtOAc 9:1). $[\alpha]_D^{25}$ = -43.2 (*c* 0.22, CH_2Cl_2), d.r. = 98:2. **^1H NMR ($CDCl_3$, 400 MHz)** δ = 7.32-7.26 (m, 2H), 7.23-7.18 (m, 1H), 7.16-7.10 (m, 2H), 4.98 (dd, *J* = 14.1, 4.9 Hz, 1H), 4.46 (dd, *J* = 14.1, 5.5 Hz, 1H), 4.16 (q, *J* = 7.1 Hz, 2H), 2.9-2.81 (m, 1H), 2.79-2.68 (m, 1H), 2.61-2.51 (m, 2H), 2.46-2.37 (m, 1H), 2.33-2.22 (m, 1H), 2.04-1.75 (m, 4H), 1.67-1.57 (m, 1H), 1.25 (t, *J* = 7.1 Hz, 3H). **$^{13}C\{^1H\}$ NMR ($CDCl_3$, 100 MHz)** δ = 213.4, 169.5, 140.8, 128.7, 128.5, 126.4, 76.5, 62.9, 62.1, 40.1, 38.2, 34.1, 32.7, 31.4, 19.4, 14.1. **HRMS (FAB+):** m/z [M+H]: Calculated for $C_{18}H_{23}NO_5$ = 334.1654, found: 334.1664. **HPLC:** (Chiralpak IC-3, hexane/ethanol 85:15, 0.6 mL/min, 220 nm): $t_{major\ enant.}$ = 7.55 min., $t_{minor\ enant.}$ = 7.98., 77% ee.

Ethyl (*R*)-1-((*S*)-2-nitro-1-(thiophen-2-yl)ethyl)-2-oxocyclopentane-1-carboxylate (**3k**)

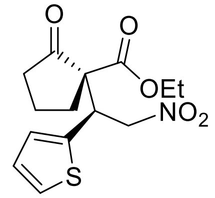

It was prepared according to the general procedure (**GP1**), *trans*-2-thiophenyl-β-nitrostyrene and 2-ethoxycarbonylcyclopentanone as starting materials. This product was purified by flash column chromatography (silica gel, hexane/EtOAc 9:1-7:3). Viscous yellow pale liquid (144.78 mg, 93% yield), R_f = 0.5 (hexane/EtOAc 8:2). $[\alpha]_D^{25}$ = -32.4 (*c* 0.18, CH_2Cl_2), d.r. = 98:2. **^1H NMR ($CDCl_3$, 400 MHz)** δ = 7.26-7.19 (dd, *J* = 5.1, 1.1 Hz, 1H), 6.96 (dd, *J* = 3.6, 1.1 Hz, 1H), 6.93 (dd, *J* = 5.1, 3.6 Hz, 1H), 5.13 (dd, 1H, *J* = 13.6, 3.5 Hz), 4.93 (dd, *J* = 13.4, 10.6 Hz, 1H), 4.41 (dd, *J* = 10.6, 3.5 Hz, 1H), 4.23 (q, *J* = 7.1 Hz, 1H), 2.51-2.34 (m, 2H), 2.21-2.06 (m, 2H), 2.06-1.95 (m, 1H), 1.95-1.84 (m, 1H), 1.29 (t, *J* = 7.1 Hz, 3H). **$^{13}C\{^1H\}$ NMR ($CDCl_3$, 100 MHz)** δ = 212.5, 169.4, 137.7, 128.8, 126.9, 126.2, 77.7, 62.5, 62.4, 42.2, 38.1, 31.7, 19.5, 14.0. **HRMS (EI/TOF):** *m/z*: Calculated for $C_{14}H_{17}NO_5S$: 311.0827, found: 311.0837. **HPLC:** (Chiralpak IC-3, hexane/ethanol 85:15, 0.6 mL/min, 220 nm): $t_{major\ enant.}$ = 10.26 min., $t_{minor\ enant.}$ = 8.57 min., 97% ee.

Ethyl (*R*)-1-((*S*)-2-nitro-1-(quinolin-3-yl)ethyl)-2-oxocyclopentane-1-carboxylate (**3l**)

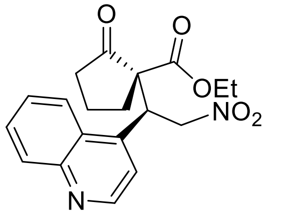

It was prepared according to the general procedure (**GP1**), *trans*-4-quinolin-β-nitrostyrene and 2-ethoxycarbonylcyclopentanone as starting materials. This product was purified by flash column chromatography (silica gel, hexane/EtOAc 7:3-4:6). Pale yellow solid (156.81 mg, 88% yield), R_f = 0.33(hexane/EtOAc 5:5), m.p. = 121-123ºC, $[\alpha]_D^{25}$ = -3.2 (*c* 0.94, CH_2Cl_2), d.r. = 97:3. **^{1}H NMR ($CDCl_3$, 400 MHz)** δ = 8.90 (d, *J* = 4.7 Hz, 1H), 8.25-8.17 (m, 1H), 8.15-8.09 (m, 1H), 7.77-7.72 (m, 1H), 7.68-7.62 (m, 1H), 7.60 (d, *J* = 4.65, 1H), 5.51 (dd, *J* = 14.0, 3.7 Hz, 1H), 5.14 (dd, *J* = 14.0, 10.3 Hz, 1H), 4.25-4.16 (m, 2H), 2.54-2.41 (m, 1H), 3.36-2.20 (m, 2H), 1.96-1.78 (m, 3.45), 1.23 (t, *J* = 7.2, 3H). **^{13}C{^{1}H} NMR ($CDCl_3$, 100 MHz)** δ = 212.2, 169.3, 149.9, 148.8, 143.2, 130.7, 129.7, 127.7, 127.5, 122.9, 120.1, 77.0, 62.6, 62.2, 38.7, 37.7, 32.9, 19.4, 14.0. **HRMS (EI/TOF):** *m/z* = Calculated for $C_{19}H_{20}N_2O_5$: 356.1372, found: 356.1367. **HPLC:** (Chiralpak IC-3, hexane/ethanol 85:15, 0.6 mL/min, 220 nm): major diastereomer: $t_{major\ enant.}$ = 20.74 min., $t_{minor\ enant.}$ = 42.94 min., 98% ee; minor diastereomer: $t_{major\ enant.}$ = 29.21 min., $t_{minor\ enant.}$ = 38.99 min., 90% ee.

Ethyl (*R*)-2-((*S*)-2-nitro-1-phenylethyl)-1-oxo-2,3-dihydro-1H-indene-2-carboxylate (**4**)

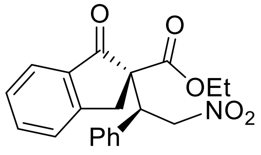

Prepared according to **GP1** using *trans*-β-nitrostyrene and ethyl 1-oxo-2,3-dihydro-1*H*-indene-2-carboxylate as ketoester. This product was purified by flash column chromatography (silica gel, hexane/EtOAc 9:1-7:3). Yellow solid (155.48 mg, 88% yield), R_f = 0.33 (hexane/EtOAc 8:2), m.p. = 129-131ºC, $[\alpha]_D^{25}$ = +14.6 (*c* 0.85, CH_2Cl_2), d.r. = 66:34. **^{1}H NMR ($CDCl_3$, 400 MHz)** δ = 7.80-7.72 (m, 1.1H), 7.74-7.62 (m, 0.6H), 7.58-7.52 (m, 0.5H), 7.52-7.45 (m, 1.2H), 7.43-7.28 (m, 2H)7.26-7.16 (m, 4H), 7.14-7.08 (m, 4H), 5.41 (dd, *J* = 13.6, 3.6 Hz, 0.5H), 5.17 (dd, *J* = 13.5, 10.9 Hz, 1.5H), 5.05 (dd, *J* = 13.4, 3.6 Hz, 1H), 4.47(dd, *J* = 11.0, 3.6 Hz, 1H), 4.26-4.08 (m, 3.5H), 3.75-3.58 (m,0.5H), 3.48 (d, *J* = 17.5 Hz, 1H), 3.28-3.07 (m,1.5H), 1.24-1.13 (m, 4.6H). **^{13}C{^{1}H} NMR ($CDCl_3$, 100 MHz)** δ = 202.2, 200.1, 170.7, 169.4, 152.6, 152.5, 136.3, 135.9, 135.8, 135.0, 134.2, 129.2, 129.1, 128.9, 128.7, 128.4, 128.1, 128.1, 126.6, 126.2, 126.2, 125.4, 125.2, 124.5, 77.3, 77.0, 63.0, 62.5, 62.5, 61.9, 47.6, 47.2, 36.7, 35.3, 14.0, 14.0. **HRMS (EI/TOF):** *m/z* = Calculated for $C_{20}H_{19}NO_5$: 353.1263, found: 353.1251. **HPLC:** (Chiralpak IC-3, hexane/ethanol 85:15, 0.6 mL/min, 220 nm): major diastereomer: $t_{major\ enant.}$ = 16.11 min., $t_{minor\ enant.}$ = 8.66 min., 88% ee; minor diastereomer: $t_{major\ enant.}$ = 9.5 min., $t_{minor\ enant.}$ = 7.98 min., 52% ee.

Methyl (R)-1-((S)-2-nitro-1-phenylethyl)-2-oxocycloheptane-1-carboxylate (**5**)

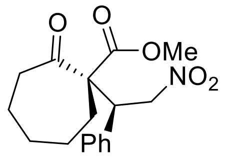

Prepared according to **GP1** using *trans*-β-nitrostyrene and methyl 2-oxocycloheptane-1-carboxylate as ketoester. This product was purified by flash column chromatography (silica gel, hexane/EtOAc 95:5-8:2). White solid (132.53 mg, 83% yield), R_f = 0.37 (hexane/EtOAc 85:15), m.p. = 58-60ºC, $[\alpha]_D^{25}$ = +29.5 (*c* 0.96, $CHCl_3$), d.r. = 94:6. **^{1}H NMR ($CDCl_3$, 400 MHz)** δ = 7.33-7.26 (m, 3H), 7.18-7.12 (m, 2H), 5.01-4.87 (m, 2H), 4.10-4.02 (m, 1H), 3.77 (s, 3H), 2.67-2.46 (m, 2H), 1.95-1.84 (m, 1H), 1.81-1.50 (m, 6H), 1.47-1.35 (m, 1H). **^{13}C{^{1}H} NMR ($CDCl_3$, 100 MHz)** δ = 208.3, 171.4, 135.7, 129.7, 128.8, 128.4, 77.9, 65.6, 52.5, 48.6, 41.5, 33.0, 29.1, 25.2, 24.7. **HRMS (DART/TOF):** m/z $[M+H]^+$: Calculated for $C_{17}H_{21}NO_5$: 320.1493, found: 320.1490. **HPLC:** (Chiralpak IC-3, hexane/ethanol 90:10, 1.0 mL/min, 220 nm): major diastereomer: $t_{major\ enant.}$ = 8.38 min., $t_{minor\ enant.}$ = 10.29 min., 96% ee; minor diastereomer: only one enantiomer, t = 8.88 min.

Ethyl (2*R*,3*S*)-2-acetyl-2-methyl-4-nitro-3-phenylbutanoate (**6**)

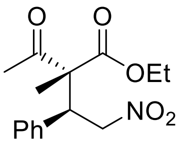

Prepared according to **GP1** using *trans*-β-nitrostyrene and ethyl 2-methyl-3-oxobutanoate as ketoester. This product was purified by flash column chromatography (silica gel, hexane/EtOAc 9:1-8:2). White solid (126.13 mg, 86% yield), R_f = 0.33 (hexane/EtOAc 85:15), m.p. = 64-66ºC, $[\alpha]_D^{25}$ = -26.7 (*c* 0.57, $CHCl_3$), d.r. = 89:11. **^{1}H NMR ($CDCl_3$, 400 MHz)** δ = 7.34-7.25 (m, 3.65H), 7.24-7.19 (m, 2H), 7.15-7.11 (m, 0.26H), 4.99-4.94 (m, 2H), 4.94-4.90 (m, 0.19H), 4.31-4.26 (m 0.26H), 4.26-4.20 (m, 1H), 4.17-4.09 (m, 1H), 4.09-4.00 (m, 1.12H), 2.17 (s, 0.38H), 2.13 (s, 3H), 1.44 (s, 3H), 1.31 (t, *J* = 7.2 Hz, 0.43H), 1.24 (s, 0.34H), 1.20 (t, *J* = 7.2 Hz, 3H). **^{13}C{^{1}H} NMR ($CDCl_3$, 100 MHz)** δ = 205.5, 204.3, 171.3, 170.9, 135.6, 135.5, 129.3, 129.1, 128.9, 128.8, 128.4, 77.1, 62.2, 62.0, 47.8, 47.4, 27.7, 26.5, 20.2, 18.2, 14.0, 13.9. **HRMS (EI/TOF):** *m/z* = Calculated for $C_{15}H_{19}NO_5$ = 293.1263, found: 293.1282. **HPLC:** (Chiralpak IA 250, hexane/ethanol 85:15, 1.0 mL/min, 220 nm): major diastereomer: t_{major} = 6.54 min., t_{minor} = 7.21 min., 97% ee; minor diastereomer: one only enantiomer t = 6.09 min.

Methyl (*R*)-1-((*S*)-2-nitro-1-phenylethyl)-2-oxocyclohexane-1-carboxylate (**7**)

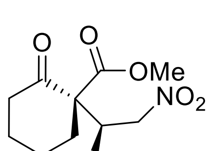

trans-β-nitrostyrene (74.6 mg, 0.5 mmol, 1 equiv.) and the bifunctional organocatalyst **C2** (2.6 mg, 0.005 mmol, 0.01 equiv.) were dissolved in 0.25 mL of acetone (2 M) in a vial. Subsequently, 1-(*tert*-butyl) methyl 2-oxocyclohexane-1-carboxylate (77.6 mg, 0.5 mmol, 1 equiv.) was added, and the reaction mixture was stirred at 40 ºC for 4 hrs. in a sealed vial. After completion, reaction mixture was centrifuged in Eppendorf tubes for 1 minute at 13300 RPM, and the organic layer was carefully transferred to a round bottom flask, and the pellet was rinsed trice with acetone. The solvents used for rinsing were combined with the organic layer and evaporated to dryness.[2] The product was purified by flash column

chromatography (silica gel, hexane/EtOAc 95:5-7:3). White solid (126.71 mg, 98% yield), R_f = 0.37 (hexane/EtOAc = 85:15), m.p. = 84-86ºC, $[\alpha]_D^{25}$ = -75.0 (*c* 0.34, $CHCl_3$), d.r. = 98:2. **^{1}H NMR ($CDCl_3$, 400 MHz)** δ = 7.30-7.23 (m, 3H), 7.14-7.08 (m, 2H), 5.08 (dd, *J* = 13.5, 3.3 Hz, 1H), 4.77 (dd, *J* = 13.5, 11.2 Hz, 1H), 4.00 (dd, *J* = 11.2, 3.3 Hz, 1H), 3.72 (s, 3H), 2.55-2.47 (m, 1H), 2.16-2.37 (m, 1H), 2.13-2.04 (m, 1H), 2.04-1.95 (m, 1H), 1.74-1.55 (m, 3H), 1.52-1.42 (m, 1H). **^{13}C{^{1}H} NMR ($CDCl_3$, 100 MHz)** δ = 207.0, 170.3, 135.4, 129.5, 128.6, 128.3, 77.6, 63.1, 52.6, 47.8, 41.5, 37.0, 28.0, 22.4. **HRMS (FAB+):** m/z [M+H]: Calculated for $C_{16}H_{19}NO_5$ = 306.1263, found: 306.1350. **HPLC:** (Chiralpak IC-3, hexane/ethanol 85:15, 0.6 mL/min, 220 nm): $t_{major\ enant.}$ = 9.56 min., $t_{minor\ enant.}$ = 8.27 min., 91% ee.

1-(*tert*-Butyl)-3-methyl-(*S*)-3-((*S*)-2-nitro-1-phenylethyl)-4-oxopiperidine-1,3-dicarboxylate (**8**)[3]

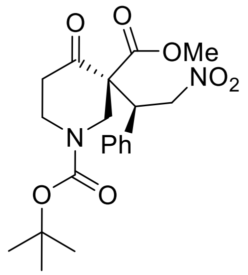

trans-β-nitrostyrene (596.6 mg, 4 mmol, 1 equiv.) and the bifunctional organocatalyst **C2** (20.7 mg, 0.04 mmol, 0.01 equiv.) were dissolved in 2.0 mL of acetone (2 M) in a vial. Subsequently, 1-(*tert*-butyl) 3-methyl 4-oxopiperidine-1,3-dicarboxylate (1.62 g, 4 mmol, 1 equiv.) was added, and the reaction mixture was stirred at 40 ºC for 4 hrs. in a sealed vial. After completion, reaction mixture was centrifuged in Eppendorf tubes for 1 minute at 13300 RPM, and the organic layer was carefully transferred to a round bottom flask, and the pellet was rinsed thrice with acetone. The solvents used for rinsing were combined with the organic layer and evaporated to dryness.[2] The product was purified by flash column chromatography (silica gel, hexane/EtOAc 92:8-72:28). White solid (1.45 g, 88% yield), R_f = 0.53 (hexane/EtOAc = 65:35), $[\alpha]_D^{25}$ = +65.0 (*c* 0.18, $CHCl_3$), d.r. = >99:1. **^{1}H NMR (300 MHz, 55ºC, $CDCl_3$)** δ = 7.34-7.26 (m, 3H), 7.23-7.14 (m, 2H), 5.01 (dd, *J* = 13.6, 3.6 Hz, 1H), 4.85 (dd, *J* = 13.5, 10.9 Hz, 1H), 4.32-3.99 (m, 3H), 3.73 (s, 3H), 3.25(ddd, *J* = 13.5, 10.4, 4.5 Hz, 1H), 3.01 (d, *J* = 13.8 Hz, 1H), 2.68 (ddd, *J* = 14.2, 10.3, 6.5 Hz, 1H), 2.55 (dt, *J* = 14.2, 4.4 Hz, 1H), 1.39 (s, 9H). **^{13}C{^{1}H} NMR (75 MHz, 55ºC, $CDCl_3$** δ = 204.2, 168.9, 154.0, 134.3, 129.5, 128.9, 128.7, 80.9, 77.4, 63.1, 52.9, 50.8, 45.3, 43.8, 40.2, 28.2. **HRMS (DART/TOF):** (DART+): $[M+H]^+$ calcd. for **$C2_0H_{27}N_2O_7$**: 407.18183, found: 407.18209. **HPLC:** (Chiralpak IA, hexane/ethanol 96:04, 0.8 mL/min, 208 nm): $t_{major\ enant.}$ = 22.5 min., $t_{minor\ enant.}$ = 29.5 min., 97% ee.

Ethyl (S)-3-((S)-2-nitro-1-phenylethyl)-2-oxotetrahydrofuran-3-carboxylate (**9**)

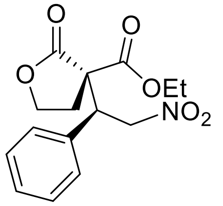

Prepared according to **GP1** using *trans*-β-nitrostyrene (74.6 mg, 0.5 mmol, 1 equiv.), ethyl 2-oxotetrahydrofuran-3-carboxylate (158.2 mg, 1 mmol, 2 equiv.) and C3 as catalyst (3.1 mg, 0.005 mmol, 0.01 equiv.). This product was purified by flash column chromatography (silica gel, hexane/EtOAc 9:1-8:2). Viscous yellow liquid (88.8 mg, 78% yield), R_f = 0.33 (hexane/EtOAc 85:15), $[\alpha]_D^{25}$ = +17.9 (*c* 0.62, $CHCl_3$), d.r. = 85:15 **^{1}H NMR ($CDCl_3$, 400 MHz)** δ = 7.36-7.25 (m, 5.88H), 5.36 (dd, *J* = 13.7, 3.7 Hz, 0.17H), 5.25 (dd, *J* = 13.6, 10.9 Hz, 1H), 5.06 (dd, *J* = 13.6, 11.2 Hz, 0.17H), 4.95 (dd, *J* = 13.6, 3.4 Hz, 1H), 4.50-4.41 (m, 0.17H), 4.36-4.22 (m, 3.51H), 4.19-4.09 (m, 1H), 3.62 (td, *J* = 8.6, 4.4 Hz, 1H), 3.52 (dd, *J* = 9.2, 7.6 Hz, 0.17H), 2.72-2.54 (m, 0.36H), 2.53-2.43 (m, 1 H), 2.35-2.22 (m, 1H), 3.34-1.28 (m, 3.52H). **^{13}C{^{1}H} NMR ($CDCl_3$, 100 MHz)** δ = 174.0, 169.4, 134.8, 134.6, 129.5, 129.2, 129.1, 129.1, 76.8, 76.2, 67.4, 66.3, 63.2, 62.3, 57.0, 46.9, 46.7 46.0, 32.0, 30.9, 14.1, 14.1. **HRMS (EI/TOF):** *m/z* = Calculated for $C_{15}H_{17}NO_6$ = 307.1056, found: 307.1033. **HPLC:** (Chiralpak AD-H 250, hexane/ethanol 85:15, 1.0 mL/min, 220 nm): t_{major} = 12.78 min., t_{minor} = 16.06 min., 96% ee.

2. General procedure 2 for the synthesis of spiro-2-pyrrolidinones (**GP2**)

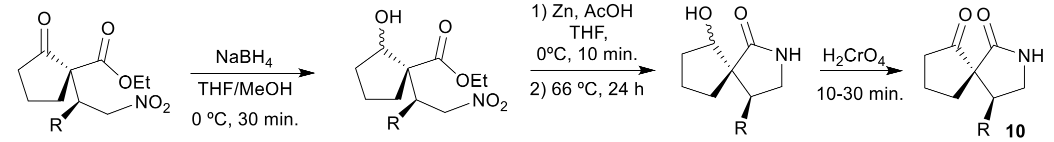

The corresponding Michael adduct (0.3 mmol, 1 equiv.) was dissolved in a 0.6 mL of a 1:1 THF/MeOH mixture (0.5 M) in a screw capped vial. The resulting solution was cooled to 0 ºC and slowly added $NaBH_4$ (22.7 mg, 0.6 mmol, 2 equiv.), and the reaction mixture was stirred at 0 °C for 10 min. After completion of the reaction was diluted with 0.33 mL of THF and added acetic acid (0.26 mL, 4.5 mmol, 15 equiv.). The reaction mixture was cooled to 0 °C, zinc powder (196.1 mg, 3.0 mmol, 10 equiv.) was added, and the mixture was stirred at 0 °C for 10 min. After completion, the vial was sealed and the reaction mixture was stirred at 66 °C for 24 h. The reaction mixture was allowed to warm to room temperature, then an aqueous NH_4Cl solution (5 mL) was added, and the mixture was extracted with ethyl acetate (3×5mL). The combined organic layers were dried over Na_2SO_4, and the solvent was evaporated under reduced pressure. After the *one-pot* reaction, the crude reaction mixture was diluted with 3.1 mL of acetone and cooled to 0 °C. Jones reagent (1.26 equiv, 0.13 mL) was then added slowly, and the mixture was stirred at 0 °C for 10 min. After completion, diethyl ether (2 mL) and isopropanol (0.07 mL) were added, and the mixture was stirred for 2 min. The solvent was evaporated under reduced pressure and the residue dissolved in 5 mL of ethyl acetate and washed with brine (5 mL × 3). The organic layer was dried over Na_2SO_4,

the solvent was evaporated under reduced pressure, and the crude product was purified by flash column chromatography.

(4*S*,5*R*)-4-phenyl-2-azaspiro[4.4]nonane-1,6-dione (**10a**)

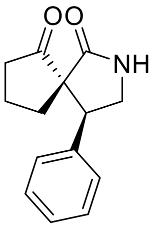

It was prepared according to the general procedure 2 (**GP2**), starting from Michael adduct **3a**. This product was purified by flash column chromatography (silica gel, EtOAc/hexane 6:4-1:0). White solid (35.77 mg, 52% yield), R_f = 0.3 (hexane/EtOAc 5:5), m.p. = 84-86ºC, $[\alpha]_D^{25}$ = +1.2 (*c* 0.26, $CHCl_3$). **^{1}H NMR ($CDCl_3$, 400 MHz)** δ = 7.37-7.22 (m, 4H), 7.19-7.11 (m, 2H), 3.95-3.81 (m, 2H), 3.61-3.51 (dd, *J* = 9.2, 4.9 Hz, 1H), 2.51-2.39 (m, 1H), 2.27-2.08 (m, 3H), 1.67-1.50 (m, 2H). **^{13}C{^{1}H} NMR ($CDCl_3$, 100 MHz)** δ = 216.9, 176.8, 139.3, 128.9, 128.0, 127.7, 62.9, 47.2, 46.2, 38.1, 29.5, 19.4. **HRMS (EI/TOF):** *m/z* = Calculated for $C_{14}H_{15}NO_2$: 229.1103, found: 229.1114.

(4*R*,5*R*)-4-(2-bromophenyl)-2-azaspiro[4.4]nonane-1,6-dione (**10b**)

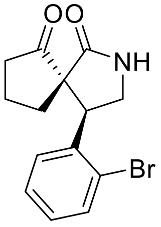

It was prepared according to the **GP3**, starting from Michael adduct **3b** (0.3 mmol, 115.27 mg). This product was purified by flash column chromatography (silica gel, EtOAc/hexane 6:4-1:0). Yellow pale solid (57.32 mg, 62% yield), R_f = 0.33 (hexane/EtOAc 5:5), m.p. = 162-164ºC, $[\alpha]_D^{25}$ = -82.3 (*c* 0.22, $CHCl_3$). **^{1}H NMR ($CDCl_3$, 400 MHz)** δ = 7.57 (dd, *J* = 8.0, 1.2 Hz, 1H), 7.36-7.28 (m, 2H), 7.14 (ddd, *J* = 8.0, 6.8, 2.2 Hz, 1H), 6.38 (br, 1H), 4.28 (dd, *J* = 7.9, 2.0 Hz, 1H), 4.11 (dd, *J* = 9.9, 7.9 Hz, 1H), 3.46 (ddd, *J* = 9.9, 2.0, 1.1 Hz, 1H), 2.51-2.33 (m, 2H), 2.24-2.12 (m, 1H), 2.06 (ddd, *J* = 13.1, 7.3, 5.5 Hz, 1H), 1.91-1.79 (m, 1H), 1.47 (dt, *J* = 13.4, 7.5 Hz, 1H). **^{13}C{^{1}H} NMR ($CDCl_3$, 100 MHz)** δ = 216.5, 175.9, 140.6, 133.1, 129.1, 128.5, 128.0, 125.3, 62.5, 47.2, 45.4, 37.8, 29.6, 19.9. **HRMS (EI/TOF):** *m/z* = Calculated for $C_{14}H_{14}BrNO_2$: 307.0208, found: 307.0219.

(4*R*,5*R*)-4-(2-chlorophenyl)-2-azaspiro[4.4]nonane-1,6-dione (**10c**)

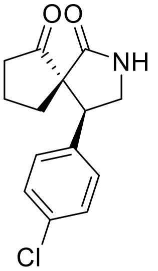

It was prepared according to the **GP3**, starting from Michael adduct **3c** (0.3 mmol, 101.93 mg). This product was purified by flash column chromatography (silica gel, EtOAc/hexane 6:4-1:0). White solid (30.06 mg, 38% yield), R_f = 0.33 (hexane/EtOAc 5:5), m.p. = 138-140ºC, $[\alpha]_D^{25}$ = -149.6 (*c* 0.23, $CHCl_3$). **^{1}H NMR ($CDCl_3$, 400 MHz)** δ = 7.32-7.27 (m, 2H), 7.14-7.05 (m, 2H), 6.70 (br, 1H), 3.92 (dd, *J* = 9.6, 7.7 Hz, 1H), 3.81 (dd, *J* = 7.7, 5.0 Hz, 1H), 3.49 (ddd, *J* = 9.6, 5.0, 0.9 Hz 1H), 2.53-2.39 (m,1H), 2.28-2.07 (m, 3H), 1.71-1.62 (m, 1H), 1.56-1.47 (m, 1H). **^{13}C{^{1}H} NMR ($CDCl_3$, 100 MHz)** δ = 216.5, 176.2, 137.9,

133.6, 129.3, 129.1, 62.6, 46.5, 46.2, 37.9, 29.5, 19.4. **HRMS (EI/TOF):** *m/z* = Calculated for $C_{14}H_{14}ClNO_2$: 263.0713, found: 263.0704.

(4*S*,5*R*)-4-(4-fluorophenyl)-2-azaspiro[4.4]nonane-1,6-dione (**10d**)

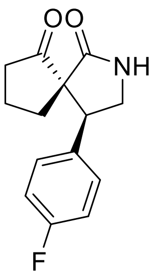

It was prepared according to the **GP3**, starting from Michael adduct **3d** (0.3 mmol, 97.0 mg). This product was purified by flash column chromatography (silica gel, EtOAc/hexane 6:4-1:0). Yellow solid (37.83 mg, 51% yield), R_f = 0.3 (hexane/EtOAc 5:5), m.p. = 134-136ºC, $[\alpha]_D^{25}$ = -131.5 (*c* 0.34, $CHCl_3$). **^{1}H NMR ($CDCl_3$, 400 MHz)** δ = 7.23-7.10 (m, 3H), 7.05-6.97 (m, 2H), 3.94-3.9 (dd, *J* = 9.2, 8.0 Hz, 1H), 3.83 (dd, *J* = 8.0, 5.0 Hz, 1H), 3.51 (dd, *J* = 9.4,5.0 Hz, 1H), 3.53-2.39 (m, 1H), 2.28-2.07 (m, 3.1H), 1.70-1.60 (m, 1H), 1.59-1.49 (m, 1H). **^{13}C{^{1}H} NMR ($CDCl_3$, 100 MHz)** δ = 216.7, 176.6, 162. (d, *J*= 246.6 Hz), 135.1 (d, *J* = 3.2 Hz), 129.6 (d, *J* = 7.8 Hz), 115.8 (d, *J* = 21.3 Hz), 62.9, 46.4, 46.4, 38.0, 29.4, 19.4. **HRMS (EI/TOF):** *m/z* = Calculated for $C_{14}H_{14}FNO_2$: 247.1009, found: 247.1001.

(4*S*,5*R*)-4-(2-(trifluoromethyl)phenyl)-2-azaspiro[4.4]nonane-1,6-dione (**10e**)

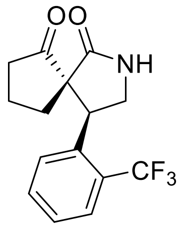

It was prepared according to the **GP3**, starting from Michael adduct **3e** (0.3 mmol, 112.0 mg). This product was purified by flash column chromatography (silica gel, EtOAc/hexane 6:4-1:0). Yellow pale solid (26.76 mg, 30% yield), R_f = 0.4 (hexane/EtOAc 6:4), m.p. = 131-133ºC, $[\alpha]_D^{25}$ = -151.8 (*c* 0.34, $CHCl_3$). **^{1}H NMR ($CDCl_3$, 400 MHz)** δ = 7.63 (dd, *J* = 8.0, 1.3 Hz, 1H), 7.56-7.50 (m, 1H), 7.46 (d, *J* = 7.9 Hz, 1H), 7.37 (t, *J* = 7.5 Hz, 1H), 6.57 (br, 1H), 4.18-4.04 (m, 2H), 3.39 (d, *J* = 9.3 Hz, 1H), 2.50-2.39 (m, 1H), 2.34-2.14 (m,2H), 2.11-2.01 (m, 1H), 1.81-1.70 (m, 1H), 1.42-1.33 (m, 1H). **^{13}C{^{1}H} NMR ($CDCl_3$, 100 MHz)** δ = 216.3, 176.2, 141.0, 132.8, 128.7 (q, *J* = 29.1 Hz), 128.18, 127.6, 125.8 (q, *J* = 5.9 Hz), 124.2 (q, *J* = 274.1 Hz), 62.8, 48.3, 41.9, 37.6, 29.9, 19.6. **HRMS (DART/TOF):** m/z $[M+H]^+$: Calculated for $C_{15}H_{14}F_3NO_2$: 298.1049, found: 298.1045.

(4*S*,5*R*)-4-(4-(trifluoromethyl)phenyl)-2-azaspiro[4.4]nonane-1,6-dione (**10f**)

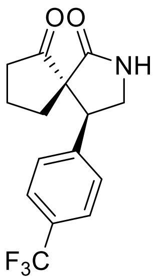

It was prepared according to the **GP3**, starting from Michael adduct **3f** (0.3 mmol, 112.0 mg). This product was purified by flash column chromatography (silica gel, EtOAc/hexane 6:4-1:0). Viscous yellow liquid (40.13 mg, 45% yield), R_f = 0.33 (hexane/EtOAc 5:5), $[\alpha]_D^{25}$ = -93.1 (*c* 0.29, $CHCl_3$). **^{1}H NMR ($CDCl_3$, 400 MHz)** δ = 7.57 (d, *J* = 7.9 Hz, 2H), 7.27 (d, *J* = 8.0 Hz, 1H), 3.94 (dd, *J* = 8.1, 1.0 Hz, 1H), 3.87 (dd, *J* = 7.7, 4.6 Hz, 1H), 3.52 (dd, *J* = 13.9, 4.7 Hz, 1H), 2.52-2.41 (m, 1H), 2.27-2.07 (m, 3H), 1.72-1.60 (m, 1H),

1.50-1.41 (m, 1H). **$^{13}C\{^1H\}$ NMR ($CDCl_3$, 100 MHz)** δ = 216.3, 176.2, 143.5, 130.1 (q, *J* = 32.7 Hz), 128.4, 125.9 (q, *J* = 3.8 Hz), 124.0 (q, *J* = 272.0 Hz), 62.6, 46.8, 46.1, 37.8, 29.5, 19.3. **HRMS (EI/TOF):** *m/z* = Calculated for $C_{15}H_{14}F_3NO_2$: 297.0977, found: 297.0952.

3-((4*S*,5*R*)-1,6-dioxo-2-azaspiro[4.4]nonan-4-yl)benzonitrile (**10g**)

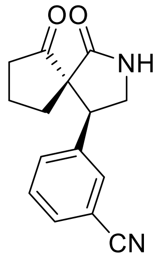

It was prepared according to the **GP3**, starting from Michael adduct **3g** (0.3 mmol, 99.10 mg). This product was purified by flash column chromatography (silica gel, EtOAc/hexane 6:4-1:0), Yellow solid (29.0 mg, 38% yield), m.p. = 119-121ºC, R_f = 0.27 (hexane/EtOAc 5:5), $[\alpha]_D^{25}$ = -174.3 (*c* 0.21, $CHCl_3$). **^1H NMR ($CDCl_3$, 400 MHz)** δ = 7.53 (dt, *J* = 7.3, 1.6 Hz, 1H), 7.43-7.37 (m, 2H), 7.35 (dt, *J* = 7.9, 1.7 Hz, 1H), 6.75 (br, 1H), 3.91 (dd, *J* = 9.7, 7.63 Hz, 1H), 3.79 (dd, *J* = 7.6, 4.7 Hz, 1H), 3.45 (dd, *J* = 14.4, 4.7 Hz), 2.48-2.38 (m, 1H), 2.25-2.12 (m, 1H), 2.10-2.02 (m, 1H), 1.69-1.59 (m, 1H), 1.42-1.31 (m, 1H). **$^{13}C\{^1H\}$ NMR ($CDCl_3$, 100 MHz)** δ = 216.0, 175.8, 141.2, 132.5, 131.5, 131.4, 129.9, 118.5, 113.2, 62.5, 46.5, 46.1, 37.7, 29.5, 19.3. **HRMS (EI/TOF):** *m/z* = Calculated for $C_{15}H_{14}N_2O_2$: 254.1055, found: 254.1048.

(4*S*,5*R*)-4-(3-methoxyphenyl)-2-azaspiro[4.4]nonane-1,6-dione (**10h**)

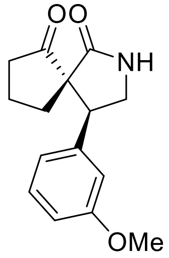

It was prepared according to the **GP3**, starting from Michael adduct **3h** (0.3 mmol, 100.61 mg). This product was purified by flash column chromatography (silica gel, EtOAc/hexane 6:4-1:0). Yellow pale solid (31.9 mg, 41% yield), R_f = 0.33 (hexane/EtOAc 3:7), m.p. = 76-78ºC, $[\alpha]_D^{25}$ = -130.9 (*c* 0.57, $CHCl_3$). **^1H NMR ($CDCl_3$, 400 MHz):** δ = 7.22 (t, *J* = 8.0 Hz, 1H), 6.79 (ddd, *J* = 8.3, 2.6, 0.9 Hz, 1H), 6.72 (d, *J* = 7.7 Hz, 1H), 6.67 (t, *J* = 2.1 Hz, 1H), 6.62 (br, 1H), 3.87 (dd, *J* = 17.1, 8.2 Hz, 1H), 3.81-3.75 (m, 4H), 3.52 (dd, *J* = 14.5, 5.1 Hz, 1H), 2.49-2.38 (m, 1H), 2.27-2.05 (m, 3H), 1.68-1.51 (m, 2H). **$^{13}C\{^1H\}$ NMR ($CDCl_3$, 100 MHz)** δ = 216.9, 176.4, 159.9, 140.9, 130.0, 120.2, 114.1, 112.6, 62.7, 55.3, 47.2, 46.1, 38.0, 29.4, 19.5. **HRMS (DART/TOF):** m/z $[M+H]^+$: Calculated for $C_{15}H_{17}NO_3$: 260.1281, found: 260.1284.

(4*S*,5*R*)-4-(3,4-dimethoxyphenyl)-2-azaspiro[4.4]nonane-1,6-dione (**10i**)

It was prepared according to the **GP3**, starting from Michael adduct **3k** (0.3 mmol, 109.61 mg). This product was purified by flash column chromatography (silica gel, EtOAc/hexane 6:4-1:0). Yellow solid (36.46 mg, 42% yield), R_f = 0.27 (hexane/EtOAc 3:7), m.p. = 139-

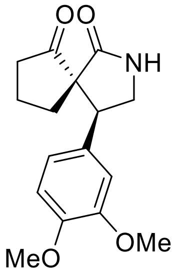

141ºC, $[\alpha]_D^{25}$ = -167.4 (*c* 0.31, $CHCl_3$). **^{1}H NMR ($CDCl_3$, 400 MHz)** δ = 6.81 (d, *J* = 8.3 Hz, 1H), 6.72 (dd, *J* = 10.3, 2.0 Hz, 1H), 6.63 (d, *J* = 2.1 Hz, 1H), 6.49 (br, 1H), 3.91-3.86 (m, 4H), 3.85 (s, 3H), 3.80 (dd, *J* = 7.7, 5.3 Hz, 1H), 3.52 (dd, *J* = 14.6, 5.4 Hz, 1H), 2.52-2.40 (m, 1H), 2.27-2.10 (m, 3H), 1.68-1.56 (m, 2H). **^{13}C{^{1}H} NMR ($CDCl_3$, 100 MHz)** δ = 217.0, 176.4, 149.2, 148.6, 131.6, 120.1, 111.3, 111.1, 62.8, 56.0, 56.0, 47.0, 46.2, 38.1, 29.5, 19.4. **HRMS (EI/TOF):** *m/z*: Calculated for $C_{16}H_{19}NO_4$: 289.1314, found: 289.1307.

(4*S*,5*R*)-4-phenethyl-2-azaspiro[4.4]nonane-1,6-dione (**10j**)

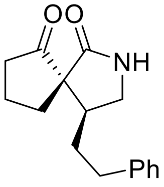

It was prepared according to the **GP3**, starting from Michael adduct **3j** (0.3 mmol, 100.0 mg). This product was purified by flash column chromatography (silica gel, EtOAc/hexane 6:4-1:0). Green solid (21.62 mg, 28% yield), R_f = 0.23 (hexane/EtOAc 5:5), m.p. = 86-88ºC, $[\alpha]_D^{25}$ = -85.4 (*c* 0.13, $CHCl_3$). **^{1}H NMR ($CDCl_3$, 400 MHz):** δ = 7.34-7.24 (m, 2H), 7.23-7.18 (m, 1H), 7.14 (d, *J* = 7.0 Hz, 2H), 6.36 (br, 0.7H), 3.60 (dd, *J* = 9.0, 7.5, 1.2 Hz, 1H), 3.03 (dd, *J* = 9.4, 7.0 Hz, 1H), 2.85-2.71 (m, 1H), 2.63-2.56 (m, 1H), 2.55-2.46 (m, 2H), 2.34-2.19 (m, 3H), 2.11-2.02 (m, 1H), 1.92-1.87 (m, 1H), 1.74-1.56 (m, 2H). **^{13}C{^{1}H} NMR ($CDCl_3$, 100 MHz)** δ = 217.1, 177.2, 141.2, 128.6, 128.3, 126.3, 61.1, 45.3, 40.9, 38.0, 33.8, 31.5, 28.2, 19.. **HRMS (EI/TOF):** *m/z* = Calculated for $C_{16}H_{19}NO_2$: 257.1416, found: 257.1410.

(4*S*,5*R*)-4-(thiophen-2-yl)-2-azaspiro[4.4]nonane-1,6-dione (**10k**)

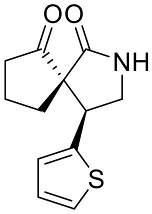

It was prepared according to the **GP3**, starting from Michael adduct **3k** (0.3 mmol, 93.41 mg). This product was purified by flash column chromatography (silica gel, EtOAc/hexane 6:4-1:0). Yellow pale solid (23.3 mg, 33% yield), R_f = 0.23 (hexane/EtOAc 4:6), m.p. = 79-81ºC, $[\alpha]_D^{25}$ = -194.0 (*c* 0.30, $CHCl_3$). **^{1}H NMR ($CDCl_3$, 400 MHz)** δ = 7.19 (dd, *J* = 5.2, 1.2 Hz, 1H), 6.95 (dd, *J* = 5.2, 3.5 Hz, 1H), 6.83 (dd, *J* = 3.5, 1.2 Hz, 1H), 6.47 (br, 1H), 4.20 (t, *J* = 6.9 Hz, 1H), 3.89 (ddd, *J* = 9.5, 7.5, 1.0 Hz, 1H), 3.50 (dd, *J* = 15.9, 6.6 Hz, 1H), 2.51-2.41 (m, 1H), 2.26-2.10 (m, 3H), 1.74-1.63 (m, 2H). **^{13}C{^{1}H} NMR ($CDCl_3$, 100 MHz)** δ = 216.3, 176.4, 141.1, 127.1, 125.8, 124.6, 62.9, 46.8, 42.9, 38.2, 29.3, 19.4. HRMS (EI/TOF): *m/z* = Calculated for $C_{12}H_{13}NO_2S$: 235.0667, found: 235.0654.

(2*R*,4'*S*)-4'-phenylspiro[indene-2,3'-pyrrolidine]-1,2'(3H)-dione (**11**)

It was prepared according to the **GP3**, starting from Michael adduct **4** (0.3 mmol, 103.01 mg). This product was purified by flash column chromatography (silica gel, EtOAc/hexane 6:4-1:0). Yellow pale solid (32.45 mg, 39% yield), R_f = 0.28 (hexane/EtOAc 5:5), m.p. =

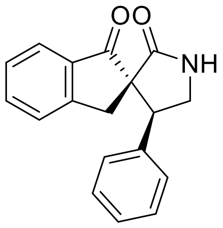

162-164ºC, $[\alpha]_D^{25}$ = +117.8 (*c* 0.23, $CHCl_3$). **¹H NMR ($CDCl_3$, 400 MHz)** δ = 7.53-7.45 (m, 2H), 7.43-7.38 (m, 1H), 7.29-7.21 (m, 3H), 7.21-7.11 (m, 3H), 6.74 (br, 1H), 4.32 (dd, *J* = 10.8, 9.2 Hz, 1H), 3.89-3.74 (m, 2H), 3.62 (ddd, *J* = 9.3, 7.9, 1.5 Hz, 1H), 3.17 (d, *J* = 17.2 Hz, 1H). **$^{13}C\{^1H\}$ NMR ($CDCl_3$, 100 MHz)** δ = 202.9, 175.6, 153.6, 135.9, 135.3, 128.6, 127.9, 127.6, 126.2, 124.3, 63.4, 51.7, 44.7, 35.1. **HRMS (EI/TOF):** *m/z* = Calculated for $C_{18}H_{15}NO_2$: 277.1103, found: 277.1103.

(4*S*,5*R*)-4-phenyl-2-azaspiro[4.5]decane-1,6-dione (**12**)

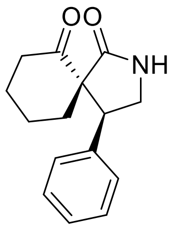

It was prepared according to the **GP3**, starting from Michael adduct **7** (0.3 mmol, 95.81 mg). This product was purified by flash column chromatography (silica gel, EtOAc/hexane 6:4-1:0). Yellow pale solid (33.58 mg, 46% yield), R_f = 0.3 (hexane/EtOAc 5:5), m.p. = 109-111ºC, $[\alpha]_D^{25}$ = +11.6 (*c* 0.32, $CHCl_3$). **¹H NMR ($CDCl_3$, 400 MHz)** δ = 7.36-7.21 (m, 5H), 6.60 (br, 1H), 4.43 (t, *J* = 7.5 Hz, 1H), 3.66 (ddd, *J* = 9.8, 7.8, 1.1 Hz, 1H), 3.59 (ddd, *J* = 9.8, 7.3, 0.8 Hz, 1H), 2.99(ddd, *J* = 14.4, 12.5, 6.2 Hz, 1H), 2.51 (dtd, *J* = 14.4, 4.0, 1.5 Hz, 1H), 2.24-2.11 (m, 1H), 2.05-1.96 (m, 1H), 1.93-1.86 (m, 1H), 1.61-1.48 (m, 2H) 1.30-1.23 (m, 1H). **$^{13}C\{^1H\}$ NMR ($CDCl_3$, 100 MHz)** δ = 207.9, 176.0, 138.2, 128.9, 128.6, 127.4, 61.8, 45.0, 44.4, 40.7, 31.8, 26.2, 20.7. **HRMS (EI-TOF):** *m/z* = Calculated for $C_{15}H_{17}NO_2$ = 243.1259, found: 243.1242.

(4*S*,5*R*)-4-phenyl-2-azaspiro[4.6]undecane-1,6-dione (**13**)

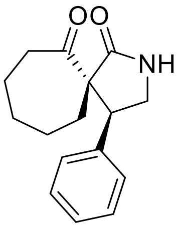

It was prepared according to the **GP3**, starting from Michael adduct **5** (0.3 mmol, 100.0 mg). This product was purified by flash column chromatography (silica gel, EtOAc/hexane 6:4-1:0). Colorless liquid (36.28 mg, 47% yield), R_f = 0.23(hexane/EtOAc 5:5), $[\alpha]_D^{25}$ = +19.3 (*c* 0.27, $CHCl_3$). **¹H NMR ($CDCl_3$, 400 MHz)** δ = 7.35-7.26 (m, 3H), 7.25-7.20 (m, 2H), 6.15 (br, 1H), 4.28 (dd, 1H, *J* = 7.5, 5.1 Hz, 1H), 3.78 (ddd, *J* = 9.7, 7.6, 0.9 Hz, 1H), 3.55 (ddd, *J* = 9.8, 5.2, 1.0 Hz, 1H), 3.10-2.99 (m, 1H), 2.49-2.40 (m, 1H), 1.95 (dd, *J* = 10.2, 6.3 Hz, , 1H), 1.91-1.82 (m, 1H), 1.67-1.56 (m, 2H), 1.54-1.41 (m, 3H), 0.81-0.71 (m, 1H). **$^{13}C\{^1H\}$ NMR ($CDCl_3$, 100 MHz)** δ = 210.6, 175.8, 139.6, 128.8, 128.5, 127.5, 65.9, 46.1, 45.6, 42.1, 31.7, 30.6, 28.3, 26.7, 24.7, 22.7, 14.2. **HRMS (EI/TOF):** *m/z* = Calculated for $C_{16}H_{19}NO_2$: 257.1416, found: 257.1427.

(3*R*,4*S*)-3-acetyl-3-methyl-4-phenylpyrrolidin-2-one (**14**)

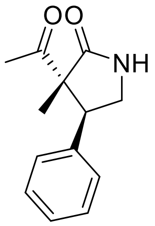

It was prepared according to the **GP3**, starting from Michael adduct **6** (0.3 mmol, 88.0 mg). This product was purified by flash column chromatography (silica gel, EtOAc/hexane 6:4-1:0). White solid (11.10 mg, 17% yield), R_f = 0.27 (hexane/EtOAc 5:5), $[\alpha]_D^{25}$ = +143.57 (*c* 0.14, $CHCl_3$). **^{1}H NMR ($CDCl_3$, 400 MHz)** δ = 7.39-7.30 (m, 3H), 7.25-7.20 (m, 2H), 6.48 (br, 1H), 3.87 (t, *J* = 9.61, 1H), 3.60 (ddd, *J* = 9.6, 7.6, 1.3 Hz, 1H), 3.48 (dd, *J* = 9.6, 7.6 Hz, 1H), 1.60 (s, 3H), 1.59 (s, 1H). **^{13}C{^{1}H} NMR ($CDCl_3$, 100 MHz)** δ = 207.9, 177.2, 135.8, 129.0, 128.2, 128.2, 61.6, 53.3, 44.1, 28.7, 19.4. **HRMS (EI/TOF):** *m/z* = Calculated for $C_{13}H_{15}NO_2$: 217.1103, found: 217.1099.

(5*S*,9*S*)-9-phenyl-2-oxa-7-azaspiro[4.4]nonane-1,6-dione (**15**)

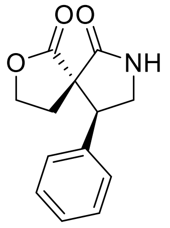

It was prepared according to the **GP3**, starting from Michael adduct **9** (0.3 mmol, 92.2 mg). This product was purified by flash column chromatography (silica gel, EtOAc/hexane 6:4-1:0). White solid (37.5 mg, 54% yield), R_f = 0.27 (hexane/EtOAc 6:4), $[\alpha]_D^{25}$ = +151.6 (*c* 0.38, $CHCl_3$), d.r. = 8:2. **^{1}H NMR ($CDCl_3$, 400 MHz)** δ = 7.45-7.29 (m, 5.7H), 7.22-7.16 (m, 0.5H), 6.58 (br, 0.3H), 6.58 (br, 1H), 4.49 (q, *J* = 8.3 Hz, 0.2H), 4.26 (td, *J* = 8.9, 3.9 Hz, 1H), 4.11 (t, *J* = 9.7 Hz, 1.2H), 4.06-3.98 (m, 0.5H), 3.71 (q, *J* = 8.3 Hz, 1H), 3.62 (dd, *J* = 10.5, 7.7 Hz, 1.25H), 3.54 (td, *J* = 8.3, 7.6, 1.5 Hz, 1H), 3.01 (td, *J* = 13.3, 8.8 Hz, 1H), 2.25 (ddd, *J* = 13.3, 7.6, 3.9 Hz, 1.25H), 1.85 (dt, *J* = 13.3, 8.7 Hz, 0.25H). **^{13}C{^{1}H} NMR ($CDCl_3$, 100 MHz)** δ = 174.1, 173.3, 134.6, 129.3, 129.2, 128.8, 128.7, 128.2, 127.9, 66.9, 66.6, 57.2, 56.5, 52.1, 47.0, 46.3, 44.4, 30.0, 28.7. **HRMS (EI/TOF):** *m/z* = Calculated for $C_{13}H_{13}NO_3$: 231.0895, found: 231.0905.

3. General procedure 3 for the synthesis of spiro-2-pyrrolidinones (**GP3**).

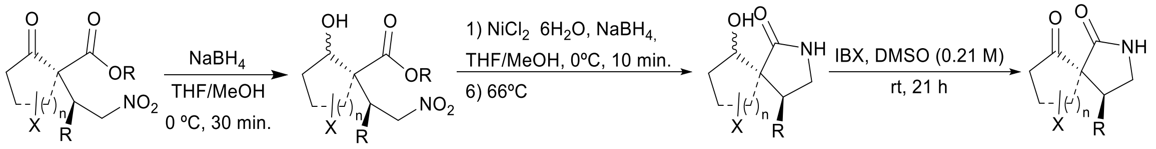

The corresponding Michael adduct (0.30 mmol, 1.0 equiv.) was dissolved in 0.6 mL of a 1:1 THF/MeOH mixture (0.5 M) in a vial and cooled to 0 °C. $NaBH_4$ (22.7 mg, 0.60 mmol, 2.0 equiv.) was then added slowly, and the reaction mixture was stirred at 0 °C for 10 min. After completion, $NiCl_2 \cdot 6H_2O$ (71.3 mg, 0.30 mmol, 1.0 equiv.) was added, and the mixture was maintained at 0 °C. Subsequently, more $NaBH_4$ (56.7 mg, 1.50 mmol, 5.0 equiv.) was added slowly, and the reaction mixture was stirred at 0 °C for 10 min. After completion, the vial was sealed and the reaction mixture was stirred at 66 °C for 24 h. The mixture was cooled to

room temperature, quenched with aqueous NH_4Cl (5 mL), and extracted with ethyl acetate (5 mL × 3). The combined organic layers were dried over Na_2SO_4 and concentrated under reduced pressure.

The crude material was diluted in 1.43 mL of DMSO (0.21 M), IBX (305.2 mg, 1.09 mmol, 3.63 equiv.) was added, and the reaction was stirred at room temperature for 21 h. The mixture was diluted with ethyl acetate (10 mL) and washed with saturated aqueous K_2CO_3 (5 mL × 3). The combined aqueous layers were extracted with ethyl acetate (15 mL). The combined organic layers were washed with brine (3 × 5 mL), dried over Na_2SO_4, filtered, and concentrated in vacuo. The crude product was purified by flash column chromatography.

(4*S*,5*R*)-4-(quinolin-3-yl)-2-azaspiro[4.4]nonane-1,6-dione (**10l**)

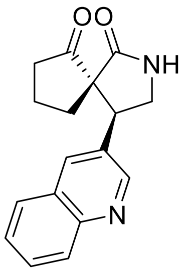

It was prepared according to the **GP3**, starting from Michael adduct **3l** (0.3 mmol, 106.9 mg). This product was purified by flash column chromatography (silica gel, EtOAc/hexane 6:4-1:0). Yellow solid (36.16 mg, 43% yield), R_f = 0.33 (EtOAc 85:15), m.p. = 164-166ºC, $[\alpha]_D^{25}$ = -21.0 (*c* 0.20, $CHCl_3$). **^{1}H NMR ($CDCl_3$, 400 MHz)** δ = 8.91 (d, *J* = 4.6 Hz, 1H), 8.16 (d, *J* = 8.5 Hz, 1H), 7.96 (d, *J* = 8.5 Hz, 1H), 7.76 (t, *J* = 7.4 Hz, 1H), 7.60 (t, *J* = 7.7 Hz, 1H), 7.38 (d, *J* = 4.6 Hz, 1H), 6.41 (s, 0.88H), 4.77 (dd, *J* = 8.0, 4.5 Hz, 1H), 4.09 (dd, *J* = 9.7, 8.0 Hz, 1H), 3.70 (dd, *J* = 9.8, 4.6 Hz, 1H), 2.57-2.42 (m, 1H), 2.26-2.10 (m, 3H), 1.60-1.48 (m, 1H), 1.43-1.33 (m, 1H). **^{13}C{^{1}H} NMR ($CDCl_3$, 100 MHz)** δ = 216.9, 175.9, 150.2, 148.6, 145.9, 130.8, 129.8, 127.4, 127.2, 122.8, 119.5, 62.4, 46.4, 40.6, 37.8, 30.0, 19.6. **HRMS (EI/TOF):** *m/z* = Calculated for $C_{17}H_{16}N_2O_2$: 280.1212, found: 280.1199.

tert-butyl (4S,5S)-1,10-dioxo-4-phenyl-2,7-diazaspiro[4.5]decane-7-carboxylate (**17**)

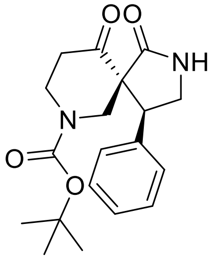

It was prepared according to the **GP3**, starting from Michael adduct **8** (0.52 mmol, 214.2 mg). This product was purified by flash column chromatography (silica gel, EtOAc/hexane 3:7-45:55). White solid (103.0 mg, 57% yield), R_f = 0.2 (EtOAc/hexane 3:7), m.p. = 84-86ºC, $[\alpha]_D^{25}$ = -0.35 (*c* 0.13, $CHCl_3$). **^{1}H NMR ($CDCl_3$, 400 MHz)** δ = 7.38-7.20 (m, 5H), 6.32 (br, 0.4H), 6.38 (br, 0.5H), 4.63-3.97 (m, 3H), 3.84-3.49 (m, 2H), 3.33-2.94 (m, 2H), 2.90-2.28 (m, 2H), 1.64-1.20 (m, 9H). **^{13}C{^{1}H} NMR ($CDCl_3$, 100 MHz)** δ = 204.7, 173.2, 154.7, 128.9, 128.6, 127.9, 80.4, 61.0, 60.8, 47.4, 46.9, 44.7, 43.9, 43.6, 43.3, 42.6, 39.9, 28.4. **HRMS (EI/TOF):** *m/z* = Calculated for $C_{19}H_{24}N_2O_4$: 344.1736, found: 344.1723.

(4*R*,5*S*)-4-phenyl-2,6-diazaspiro[4.5]decane-1,7-dione (**17**)

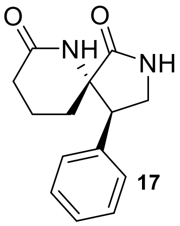

17

To a 4 mL vial were added 45.5 mg (0.2 mmol, 1 equiv.) of lactam **10a**, 137.6 mg (0.6 mmol, 3 equiv.) of sodium acetate and 41.7 mg (0.6, 3 equiv.) of hydroxylamine hydrochloride. The vial was purged with N_2, and 2 mL of anhydrous MeOH were added. The reaction mixture was heated to reflux (66 °C) for 2 h and then allowed to cool to room temperature. After completion of the reaction, the solvent was removed *in vacuo*, and the resulting residue was diluted with 2 mL of pyridine. To the solution were added 95.3 mg (0.5 mmol, 2.5 equiv.) of *p*-TsCl and 0.5 mg (0.004 mmol, 0.02 equiv.) of DMAP were then added. The mixture was heated at 85 °C for 17 h and subsequently cooled to room temperature. The reaction mixture was concentrated *in vacuo*, and the crude material was diluted with ethyl acetate (10 mL) and washed with 1 M HCl (10 mL) and saturated aqueous $NaHCO_3$ (10 mL). The combined organic layers were dried over Na_2SO_4, filtered, and concentrated.[4] The product **17** was purified by flash column chromatography (silica gel, EtOAc/hexane 9:1–5:5). White solid (19.1 mg, 39% yield), R_f = 0.17 (hexane/EtOAc 5:5), m.p. = 84-86ºC, $[\alpha]_D^{25}$ = -1.2 (0.10 *c* , DMSO). **^{1}H NMR (DMSO-*d6*, 400 MHz)** δ = 10.65 (s, 1H), 7.92 (s, 1H), 7.3-7.25 (m, 2H), 7.24-7.20 (m, 1H), 7.19-7.16 (m, 2H), 3.70 (t, *J* = 6.6 Hz, 1H), 3.59 (dd, *J* = 9.5, 7.3 Hz, 1H), 3.39 (dd, *J* = 9.6, 5.9 Hz, 1H), 2.27 (t, *J* = 7.4, 1H), 1.74-1.57 (m, 2H), 1.31-1.14 (m, 2H). **^{13}C{^{1}H} NMR (DMSO-*d6*, 100 MHz)** δ = 177.9, 165.9, 140.5, 129.0, 128.4, 127.5, 57.8, 48.8, 45.0, 31.3, 27.8, 21.4. **HRMS (EI/TOF):** *m/z* = Calculated for $C_{14}H_{16}N_2O_2$: 244.1212, found: 244.1218.

(4*S*,5*R*,6*R*)-6-hydroxy-4-phenyl-2-azaspiro[4.4]nonan-1-one (**18**)

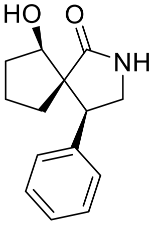

Lactam **10a** (29.8 mg, 0.13 mmol, 1 equiv.) was diluted in THF/MeOH 1:1 mixture (0.5 M, 0.26 mL) in a vial and cooled at 0ºC. $NaBH_4$ (9.8 mg, 0.26 mmol, 2 equiv.) was added to the solution, and the reaction was stirred at 0ºC for 10 minutes. After the completion, aqueous saturated solution of NH_4Cl (5 mL) was added and subsequently extracted with ethyl acetate (3 × 5 mL). The organic layers were mixed, dried with Na_2SO_4, filtered and concentrated in vacuo. White solid (29.8 mg, 99% yield), R_f = 0.33 (hexane/EtOAc 8:2), m.p. = 76-78ºC, $[\alpha]_D^{25}$ = -0.3 (*c* 0.15, CH_3OH). d. r. = 93:7. **^{1}H NMR ($CDCl_3$, 400 MHz)** δ = 7.57-7.41 (m, 0.07H), 7.39-7.21 (m, 5.8H), 7.19-7.12 (m, 0.07H), 6.34 (br, 1H), 4.52-4.39 (m, 0.07), 4.12 (q, *J* = 5.94 Hz, 1H), 3.93 (dd, *J* = 7.9, 4.2 Hz, 0.07H), 3.82 (t, *J* = 8.8 Hz, 0.07H), 3.70 (dd, *J* = 9.5, 7.9 Hz, 1H), (dd, *J* = 7.4, 16.9 Hz, 1H), 3.50 (t, *J* = 7.4 Hz, 1H), 3.42 (d, *J* = 7.1 Hz, 1H), 2.49-2.38 (m, 0.07H), 2.18-2.01 (m,0.14H), 1.95-1.71 (m, 4.14 H), 1.05, 1.40 (m, 1.07H), 1.15-1.02 (m, 1H). **^{13}C{^{1}H} NMR ($CDCl_3$, 100 MHz)** δ = 181.0, 138.9, 128.7, 127.6, 79.7, 57.9, 49.2, 45.9, 33.9, 28.7, 21.0. **HRMS (EI/TOF):** *m/z* = Calculated for $C_{14}H_{17}NO_2$: 231.1259, found: 231.1268.

(3*R*,4'*S*)-4'-phenyl-1,4-dihydro-2H-spiro[cyclopenta[b]indole-3,3'-pyrrolidin]-2'-one (**19**)

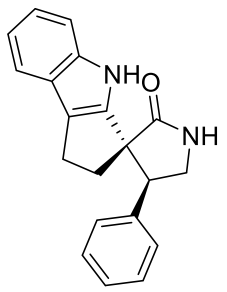

In a small vial with 25.2 mg (0.11 mmol, 1 equiv.) of lactam **10a** and 13 µL (0.13 mmol, 1.2 equiv.) of phenylhydrazine was purged with N_2 and diluted with 1.1 mL of anhydrous toluene. The reaction mixture was stirred at room temperature for 1 h, then added 56.8mg (0.33 mmol, 3 equiv.) of *p*-TsOH. The mixture was then stirred at 80 °C for 4 h. Upon completion, the reaction mixture was diluted with 10 mL of aqueous NH_4Cl solution and 10 mL of EtOAc. The organic layer was extracted with more EtOAc (10 mL × 3). The combined organic layers were dried over Na_2SO_4, filtered, and concentrated *in vacuo*.[4] The crude product was purified by flash column chromatography (silica gel, EtOAc/hexane 5:5–1:0). Yellow pale solid (19.1 mg, 39% yield), Rf = 0.1 (hexane/EtOAc 5:5), m.p. = 72-74ºC, $[\alpha]_D^{25}$ = -1.6 (*c* 0.13, $CHCl_3$). **^{1}H NMR ($CDCl_3$, 400 MHz)** δ = 8.73 (br, 1H), 7.50 (br, 1H), 7.36-7.32 (m, 1H), 7.20-7.08 (m, 4H), 7.07-6.99 (m, 2H), 6.72 (d, *J* = 6.7 Hz, 2H), 3.73-3.71 (m, 2H), 3.55-3.44 (m, 1H), 2.67-2.57 (m, 1H), 2.57-2.45 (m, 1H), 2.37-2.25 (m, 1H), 2.01-1.90 (ddd, *J* = 13.8, 7.9, 6.4 Hz, 1H). **^{13}C{^{1}H} NMR ($CDCl_3$, 100 MHz)** δ = 180.6, 142.1, 142.0, 128.4, 127.8, 127.3, 124.3, 123.1, 121.4, 119.5, 119.1, 112.3, 49.6, 43.8, 35.2, 34.8, 23.5. **HRMS (EI/TOF):** *m/z* = Calculated for $C_{20}H_{18}N_2O$: 302.1419, found: 302.1422.

4. Copies of NMR spectra of the products

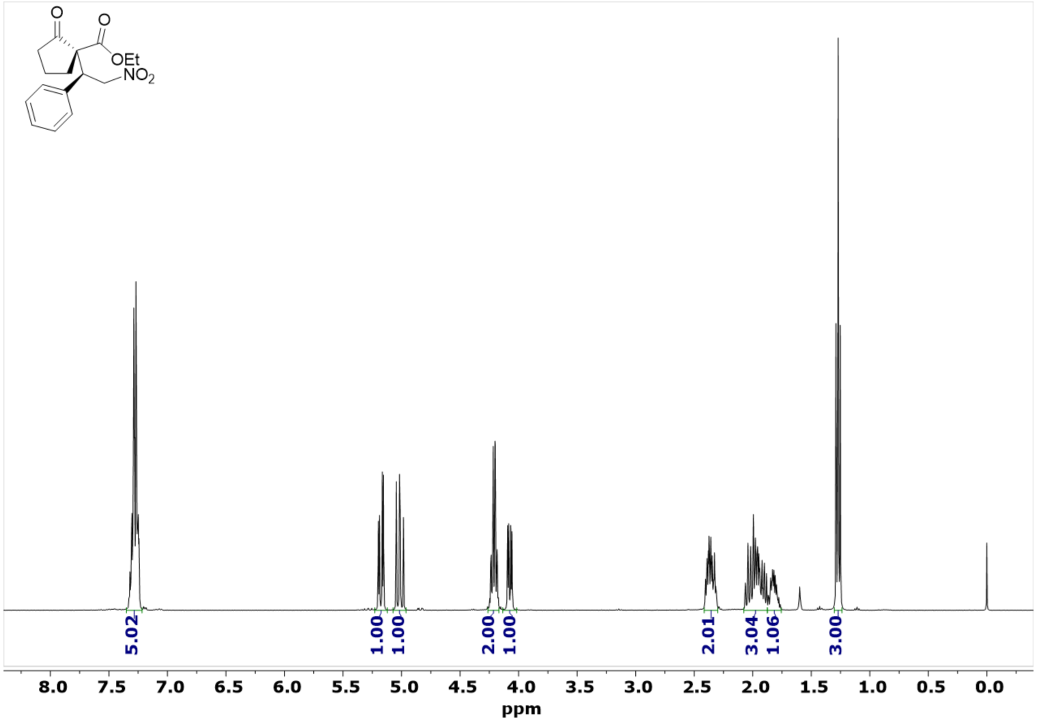

Figure S2: ^{1}H NMR, $CDCl_3$, 400 MHz (ppm) **3a**

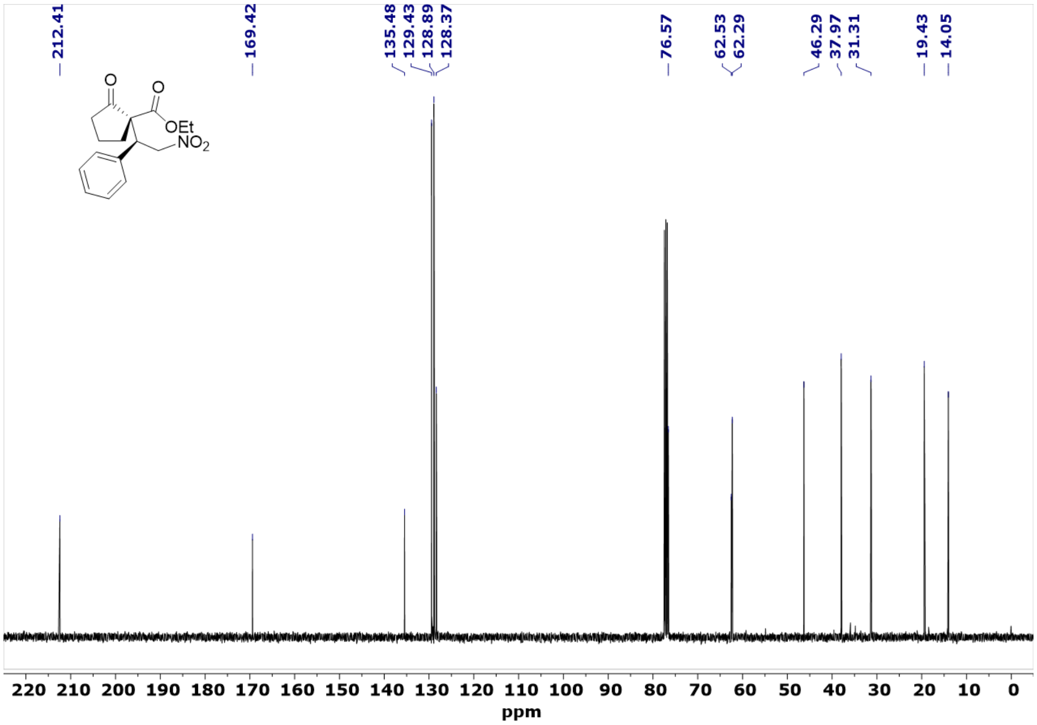

Figure S3: ^{13}C NMR, $CDCl_3$, 100 MHz (ppm) **3a**

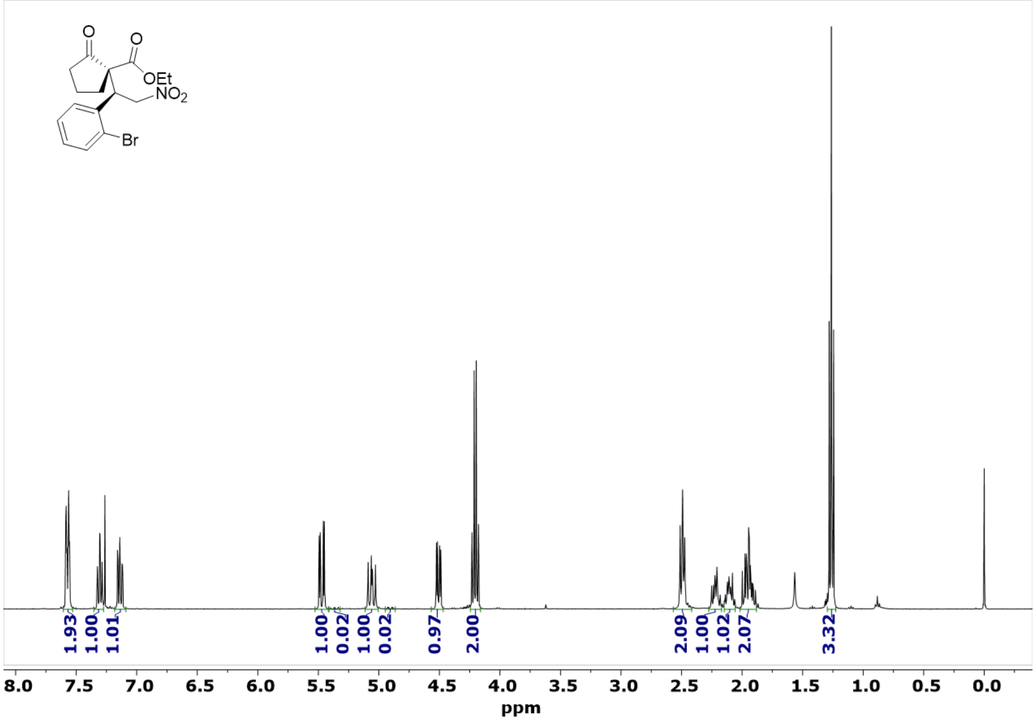

Figure S4: ^{1}H NMR, $CDCl_3$, 400 MHz (ppm) **3b**

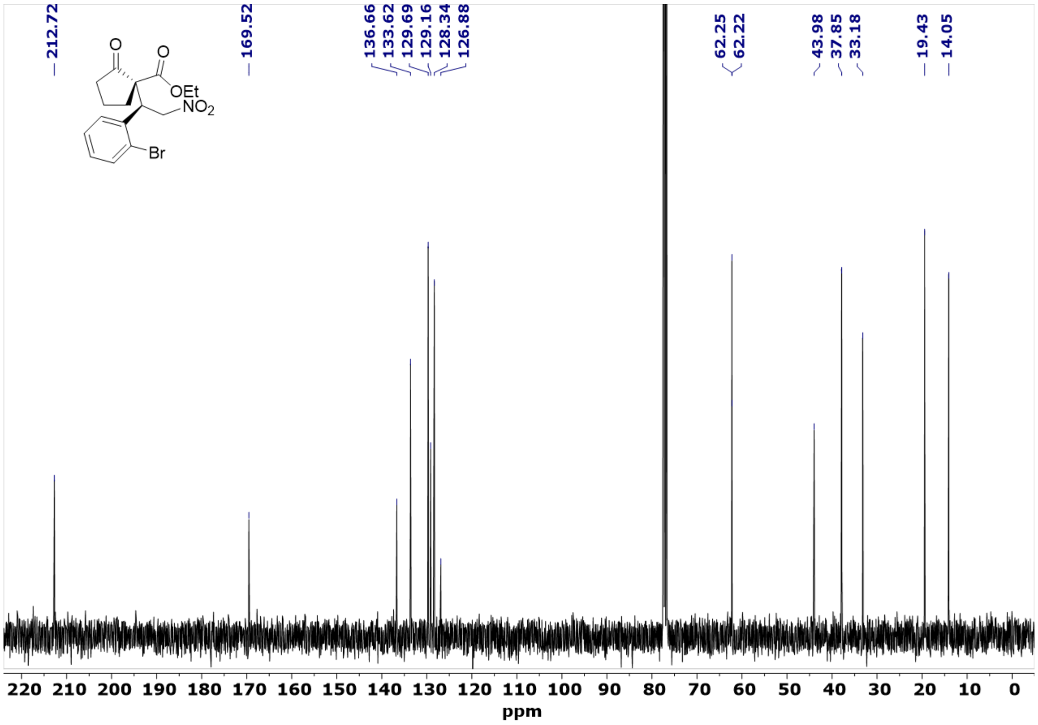

Figure S5: ^{13}C NMR, $CDCl_3$, 100 MHz (ppm) **3b**

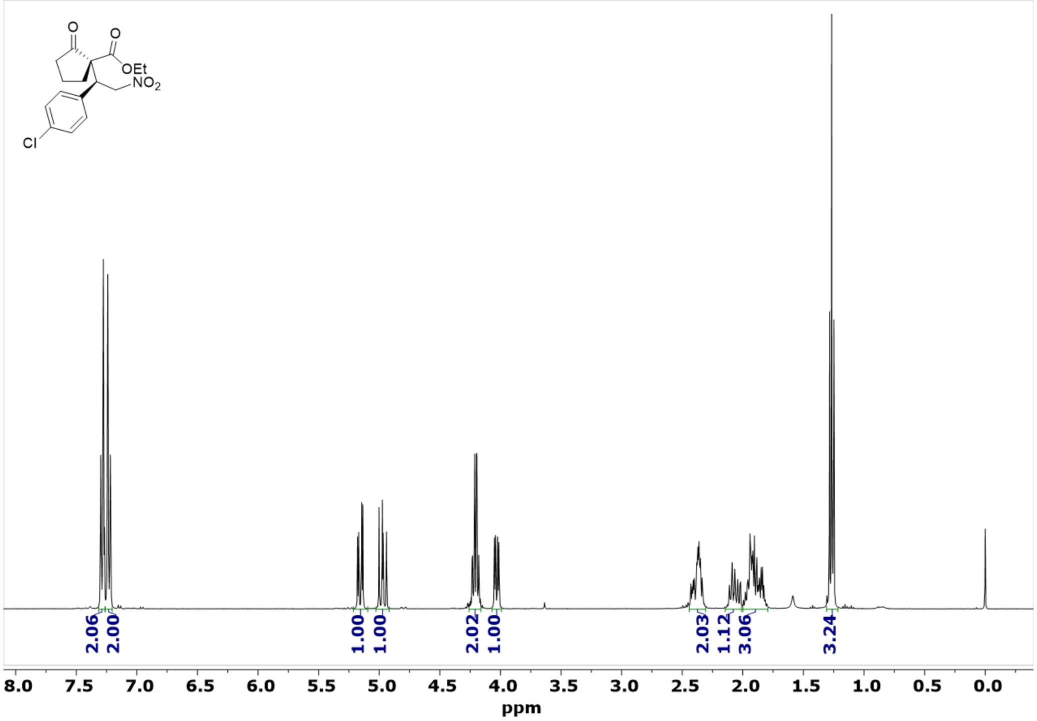

Figure S6: ^{1}H NMR, $CDCl_3$, 400 MHz (ppm) **3c**

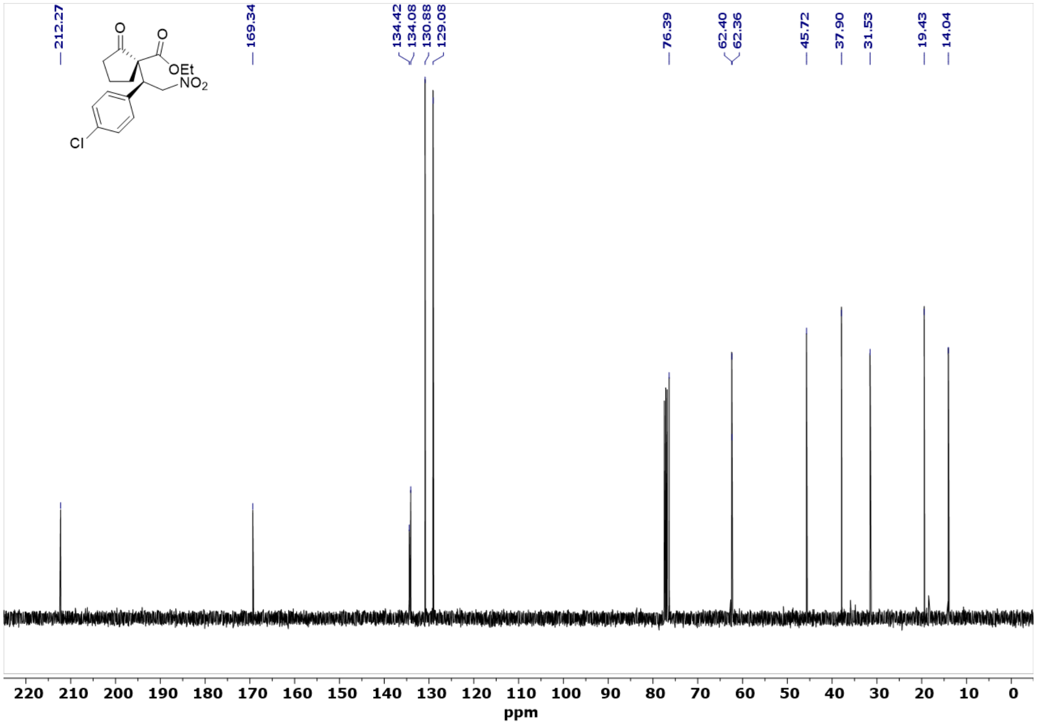

Figure S7: ^{13}C NMR, $CDCl_3$, 100 MHz (ppm) **3c**

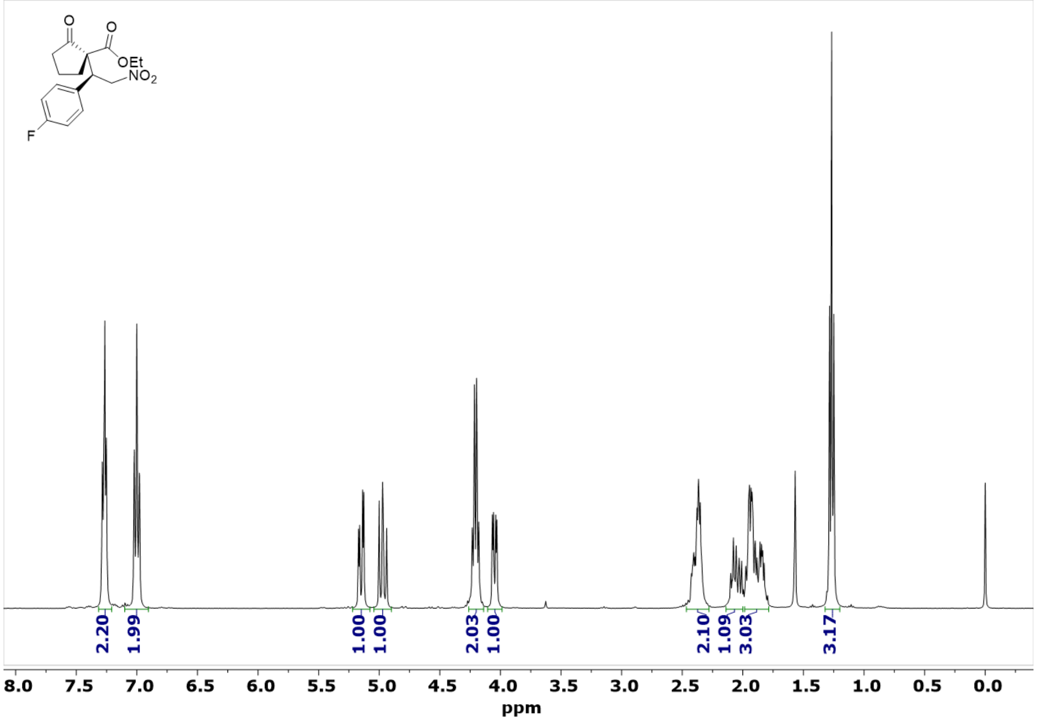

Figure S8: ^{1}H NMR, $CDCl_3$, 400 MHz (ppm) **3d**

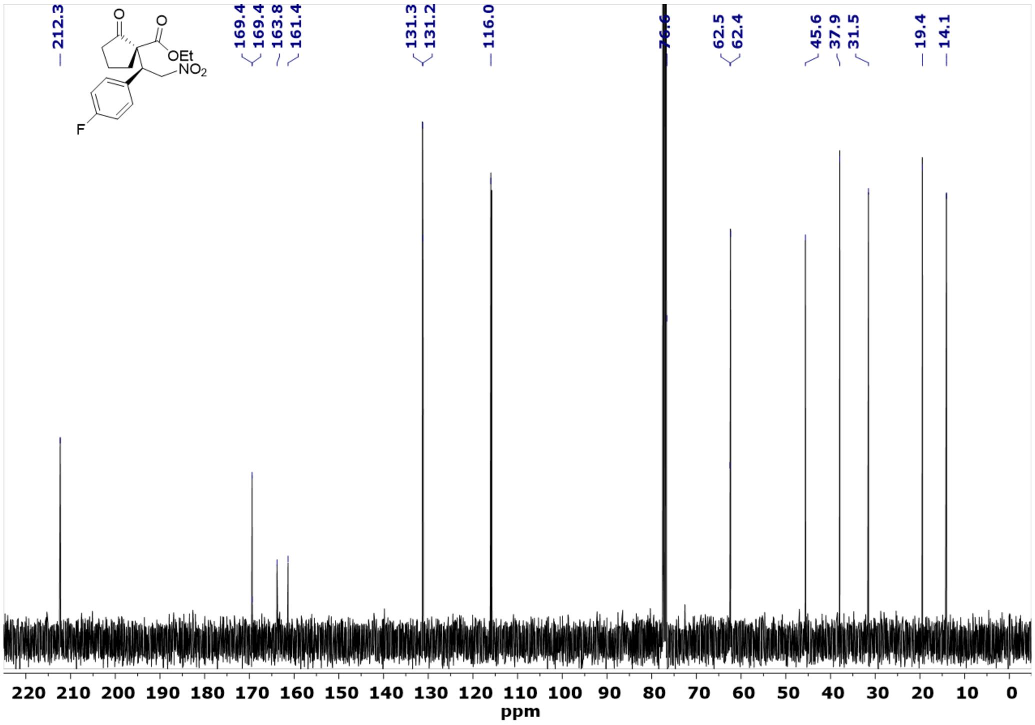

Figure S9: ^{13}C NMR, $CDCl_3$, 100 MHz (ppm) **3d**

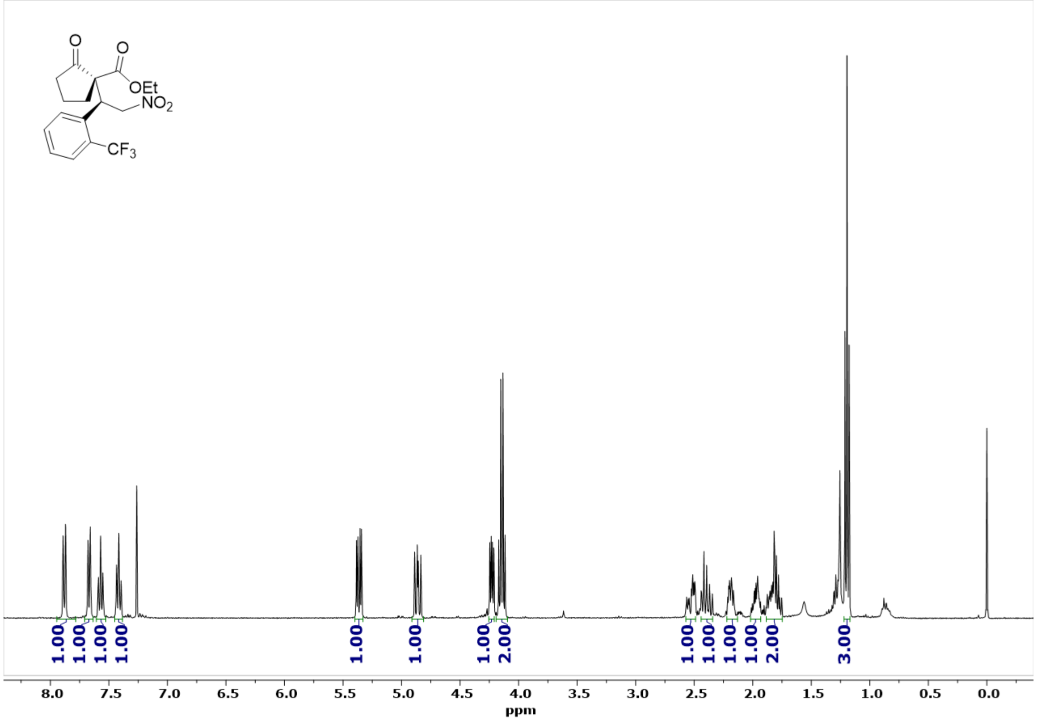

Figure S10: ^{1}H NMR, $CDCl_3$, 400 MHz (ppm) **3e**

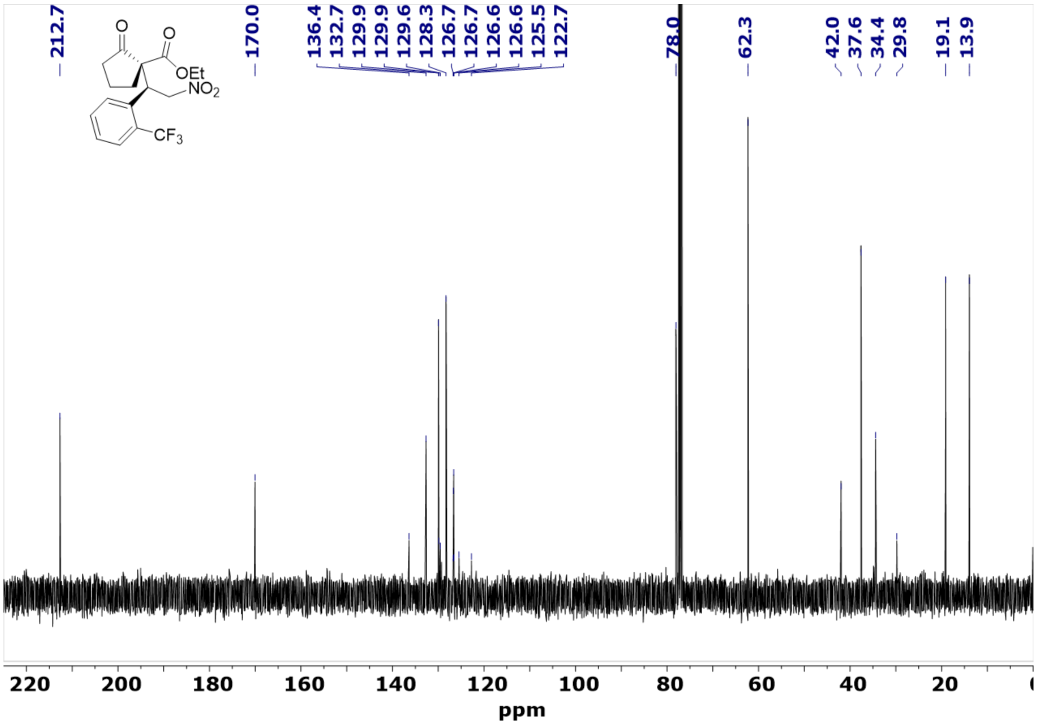

Figure S11: ^{13}C NMR, $CDCl_3$, 100 MHz (ppm) **3e**

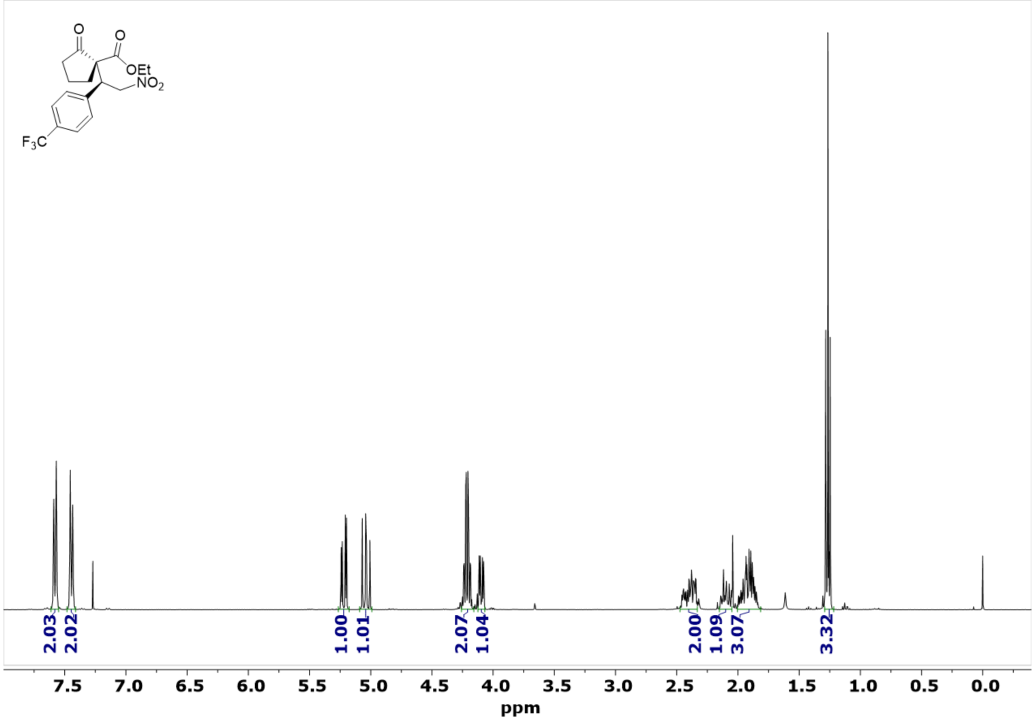

Figure S12: ^{1}H NMR, $CDCl_3$, 400 MHz (ppm) **3f**

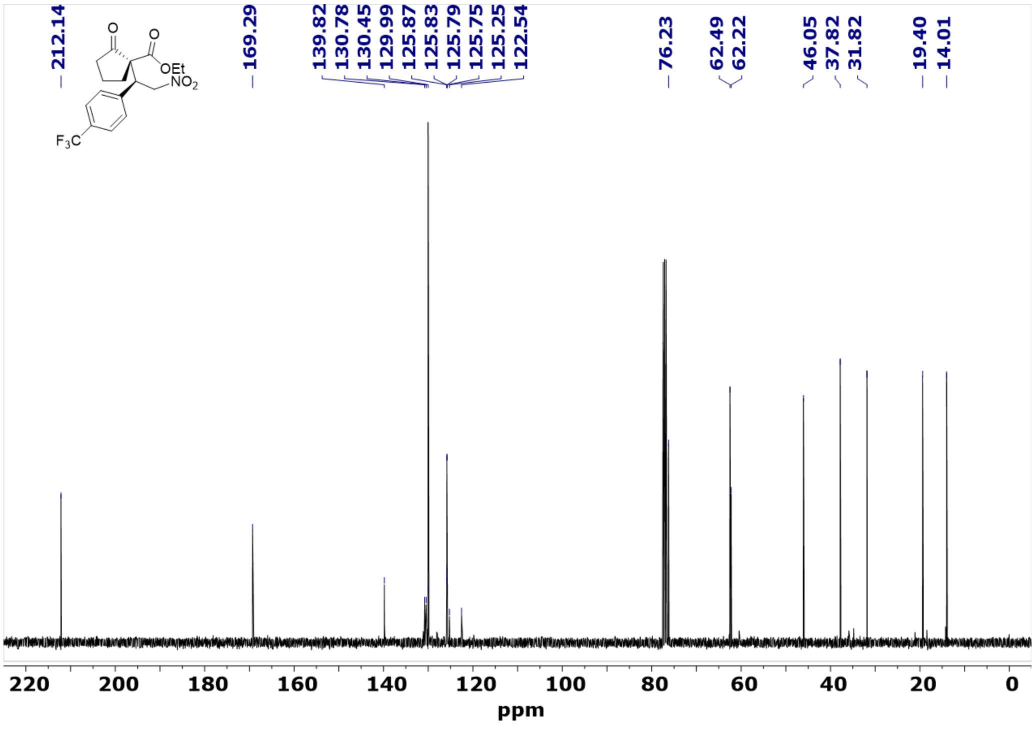

Figure S13: ^{13}C NMR, $CDCl_3$, 100 MHz (ppm) **3f**

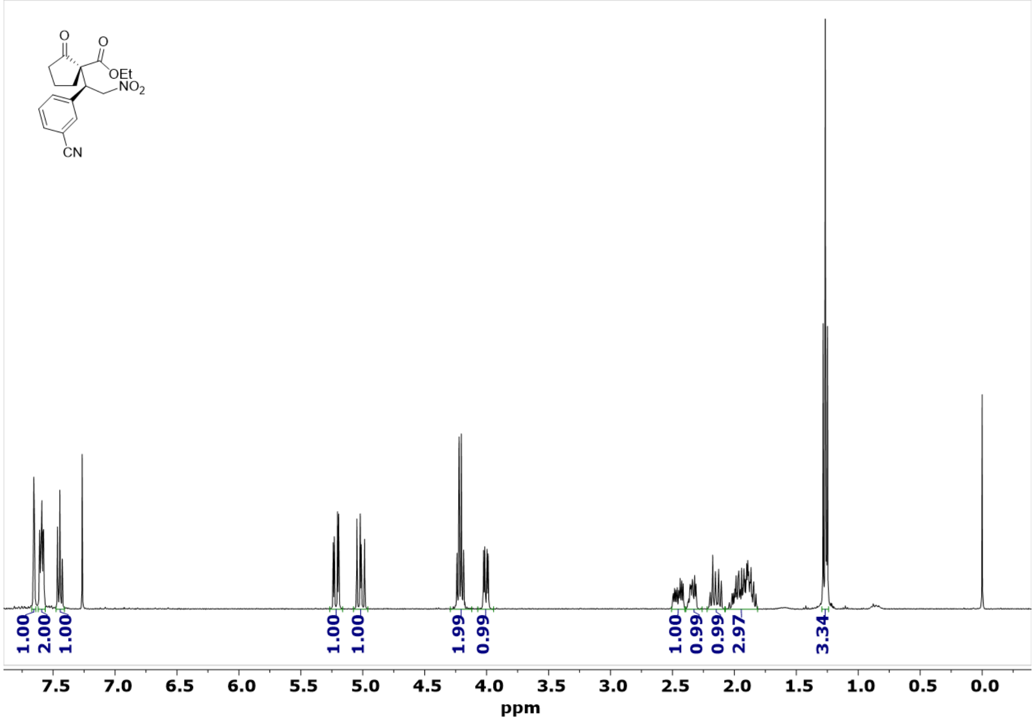

Figure S14: ^{1}H NMR, $CDCl_3$, 400 MHz (ppm) **3g**

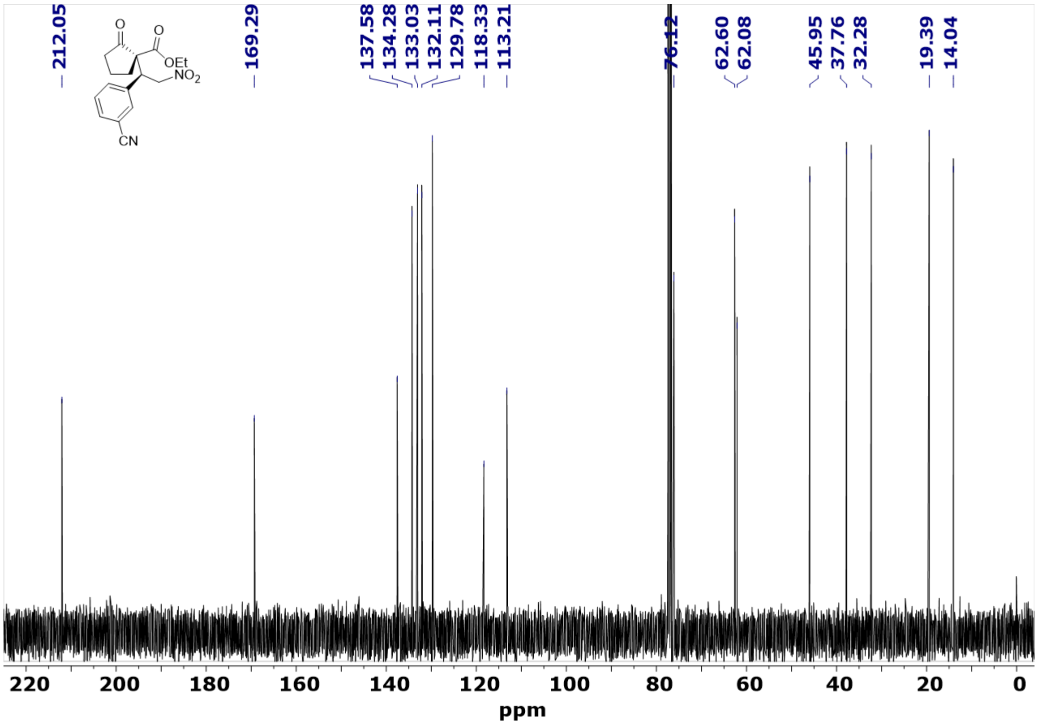

Figure S15: ^{13}C NMR, $CDCl_3$, 100 MHz (ppm) **3g**

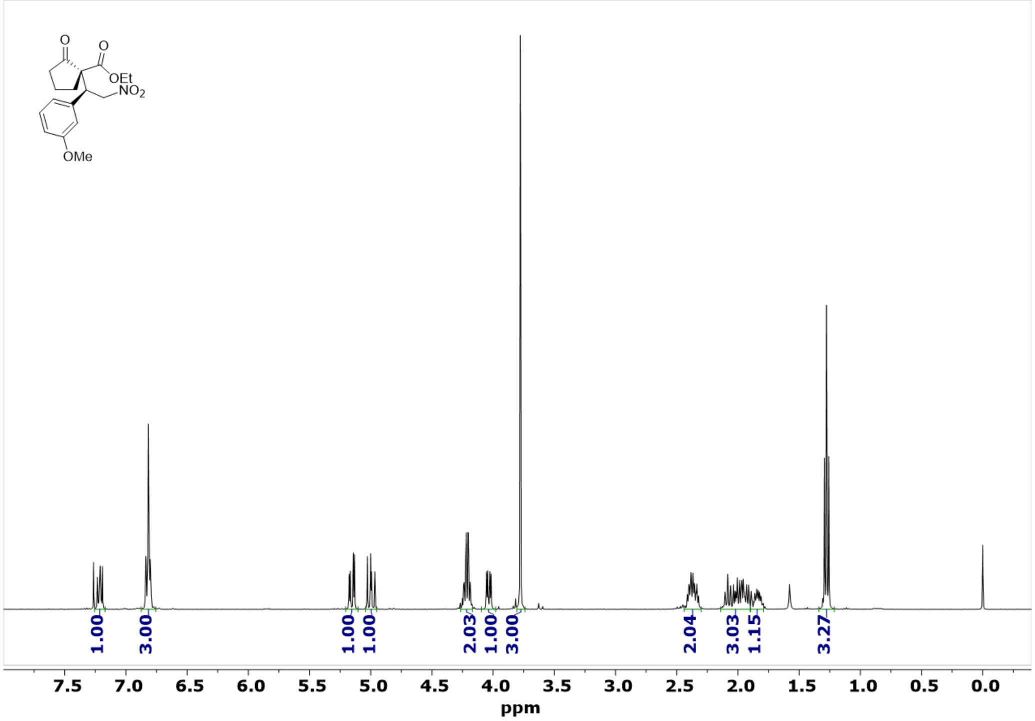

Figure S16: ^{1}H NMR, $CDCl_3$, 400 MHz (ppm) **3h**

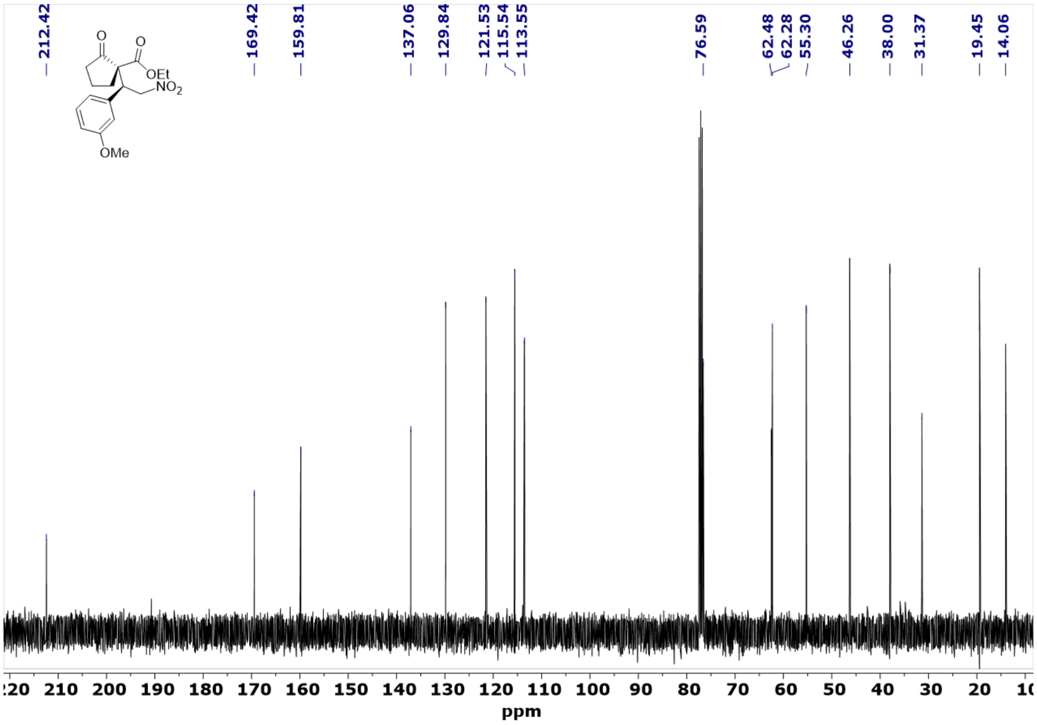

Figure S17: ^{13}C NMR, $CDCl_3$, 100 MHz (ppm) **3h**

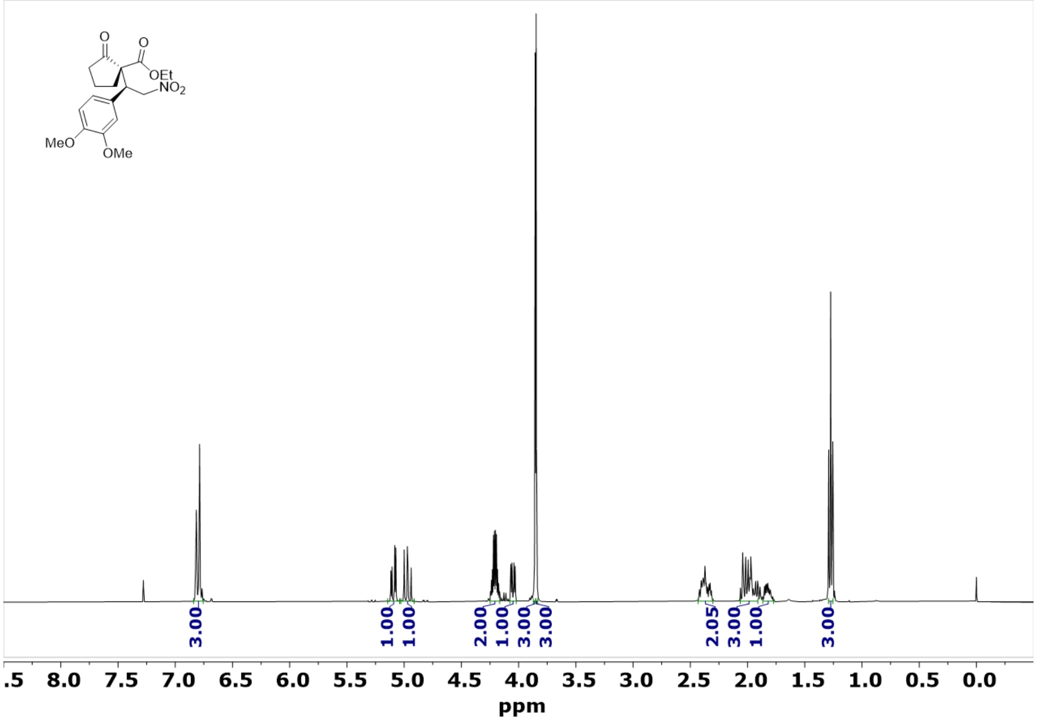

Figure S18: ^{1}H NMR, $CDCl_3$, 400 MHz (ppm) **3i**

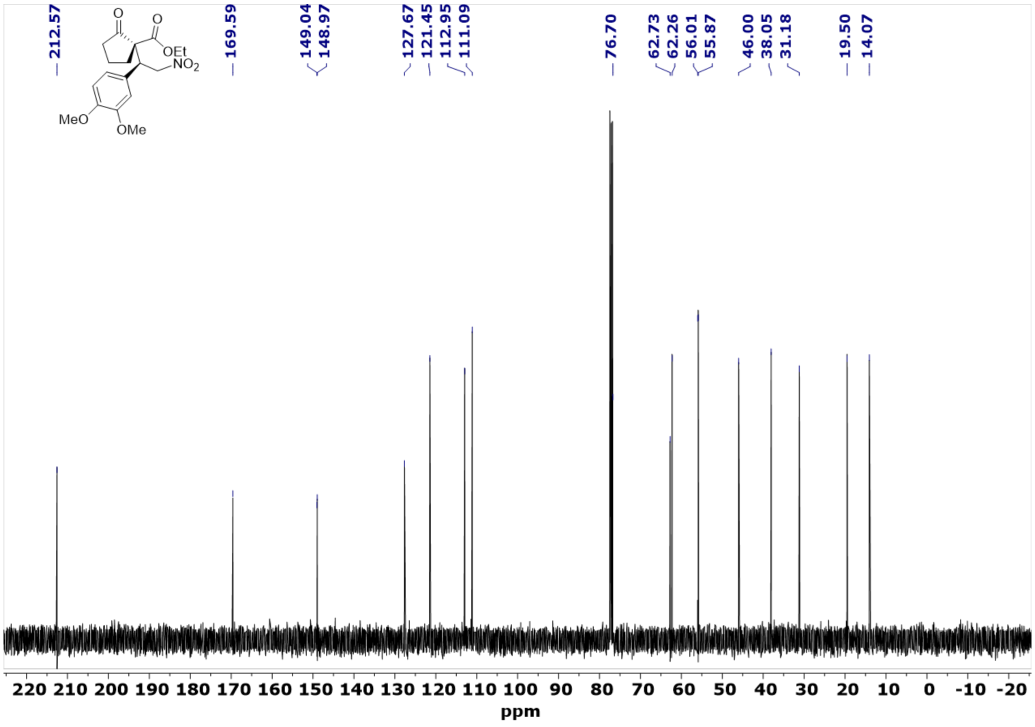

Figure S19: ^{13}C NMR, $CDCl_3$, 100 MHz (ppm) **3i**

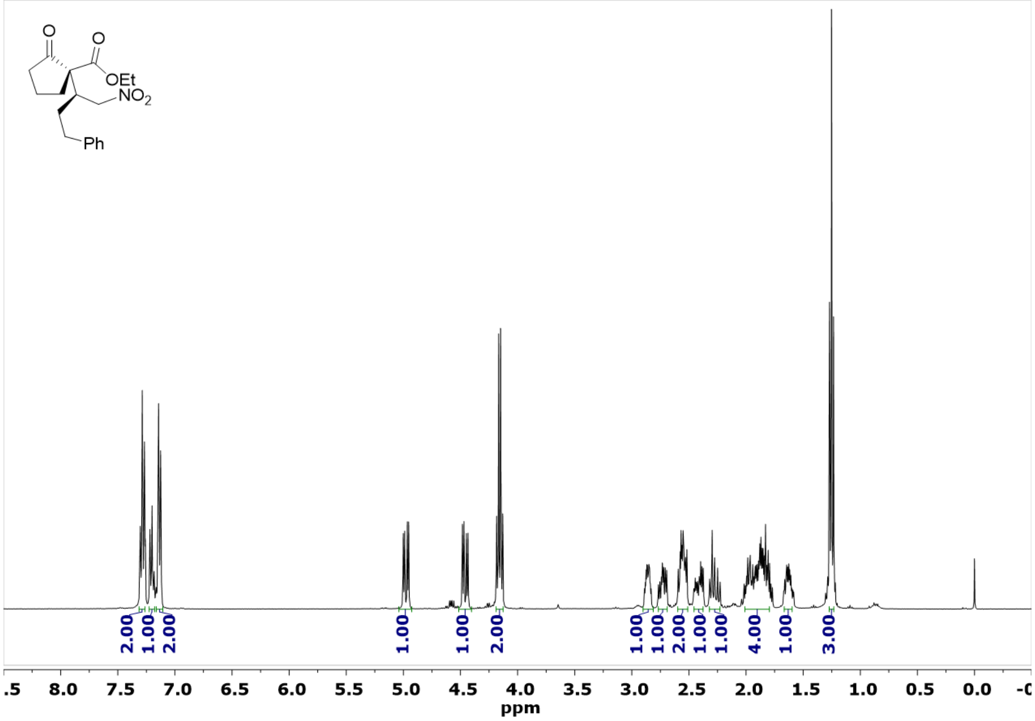

Figure S20: ^{1}H NMR, $CDCl_3$, 400 MHz (ppm) **3j**

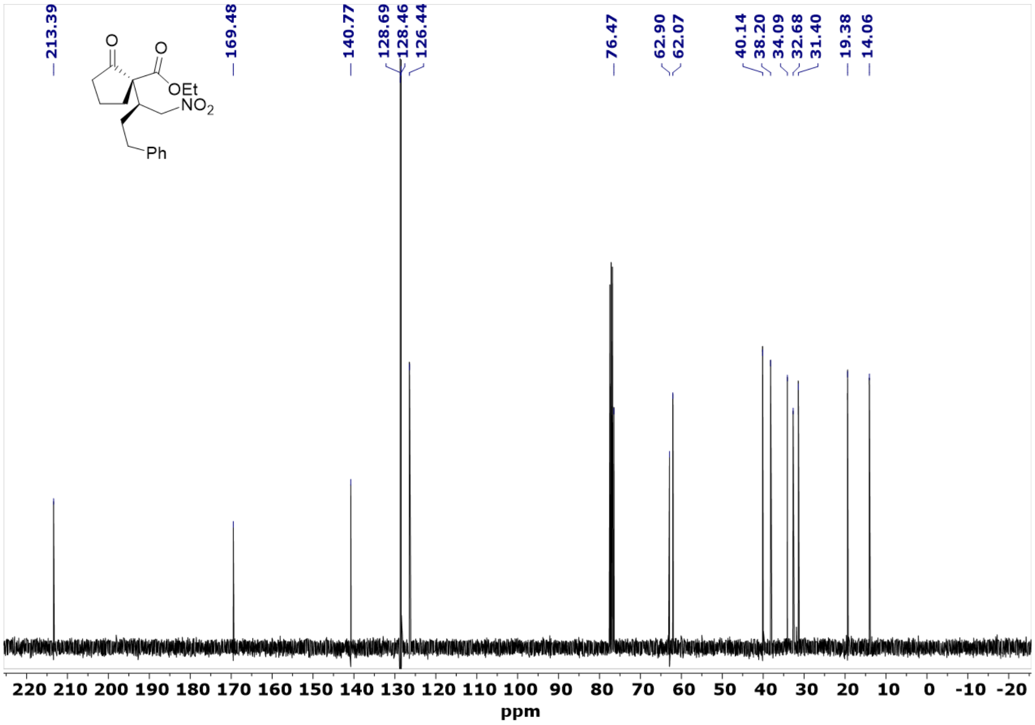

Figure S21: ^{13}C NMR, $CDCl_3$, 100 MHz (ppm) **3j**

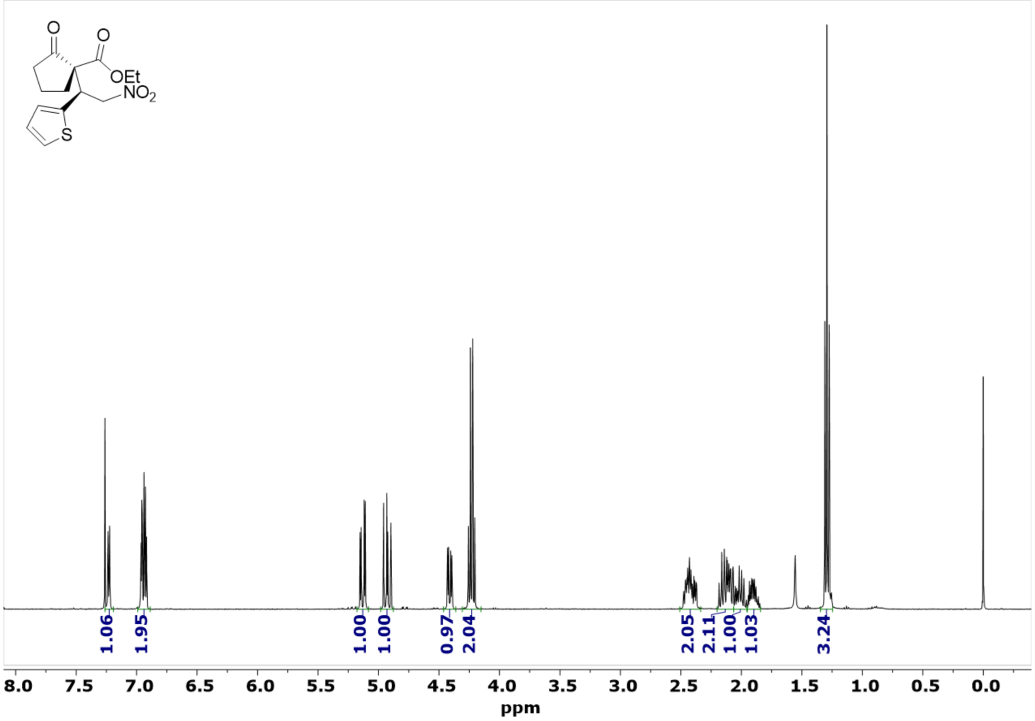

Figure S22: ^{1}H NMR, $CDCl_3$, 400 MHz (ppm) **3k**

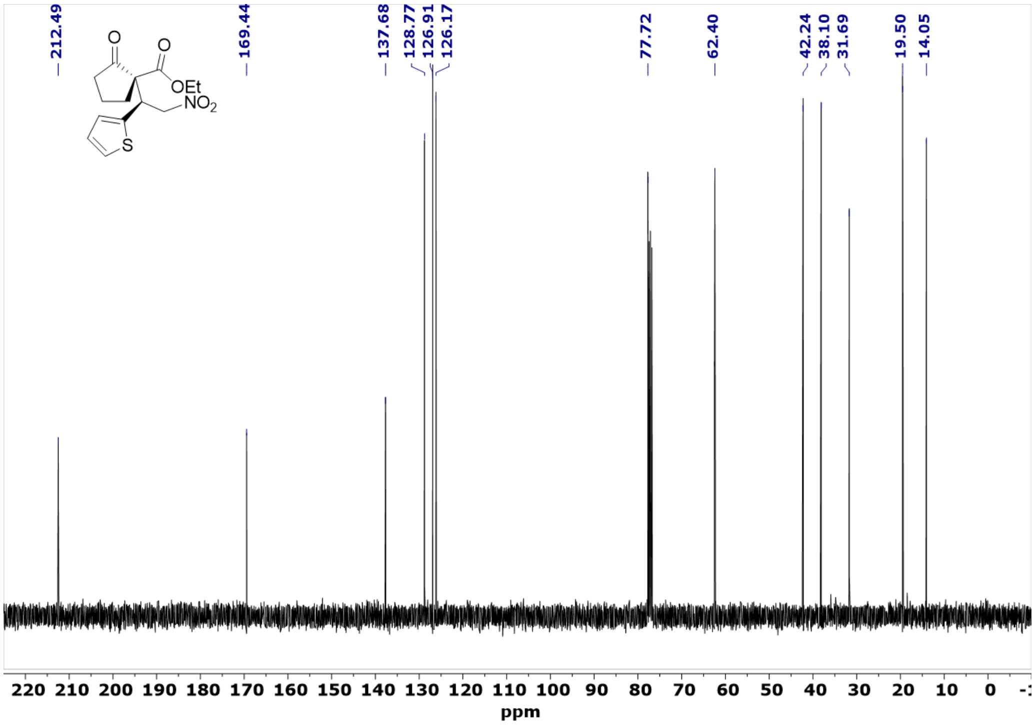

Figure S23: ^{13}C NMR, $CDCl_3$, 100 MHz (ppm) **3k**

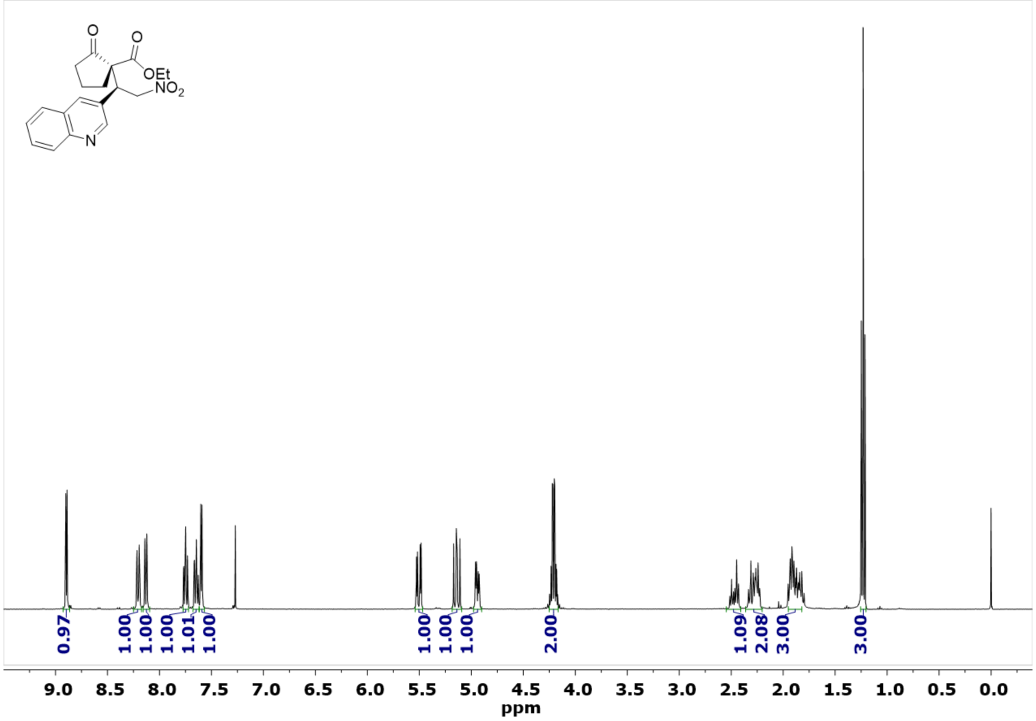

Figure S24: ^{1}H NMR, $CDCl_3$, 400 MHz (ppm) **3l**

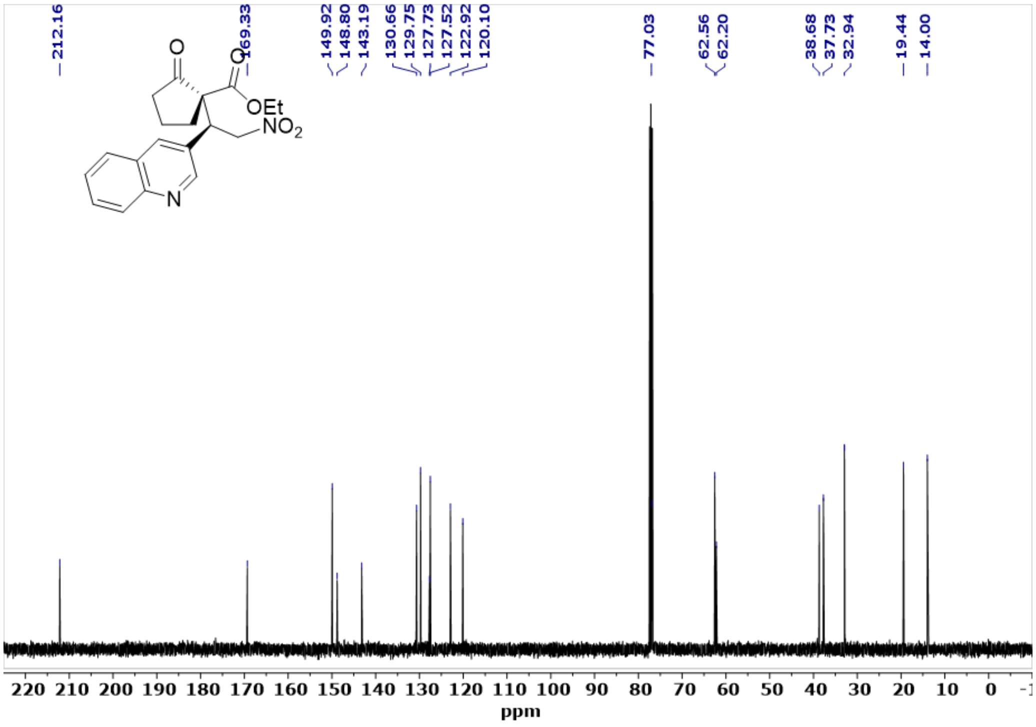

Figure S25: ^{13}C NMR, $CDCl_3$, 100 MHz (ppm) **3l**

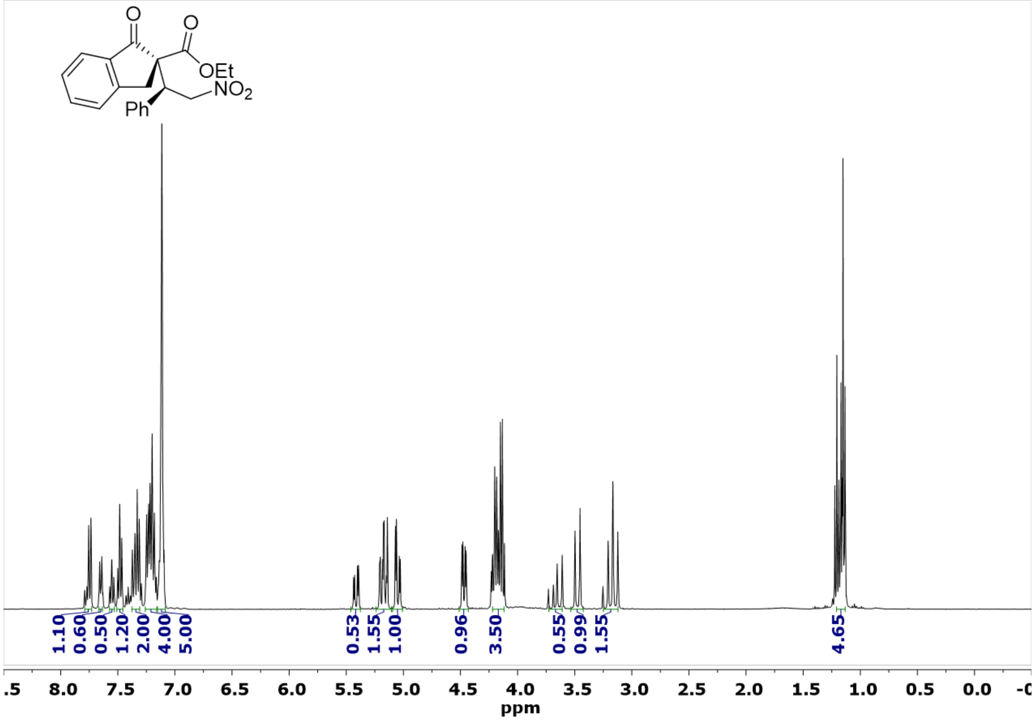

Figure S26: ^{1}H NMR, $CDCl_3$, 400 MHz (ppm) **4**

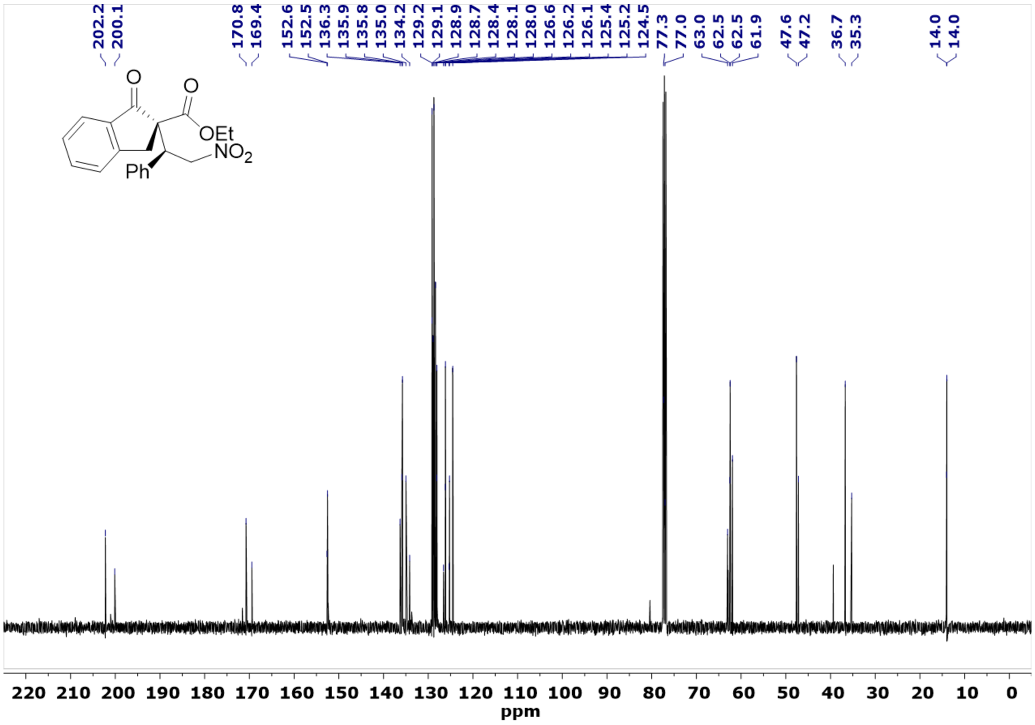

Figure S27: ^{13}C NMR, $CDCl_3$, 100 MHz (ppm) **4**

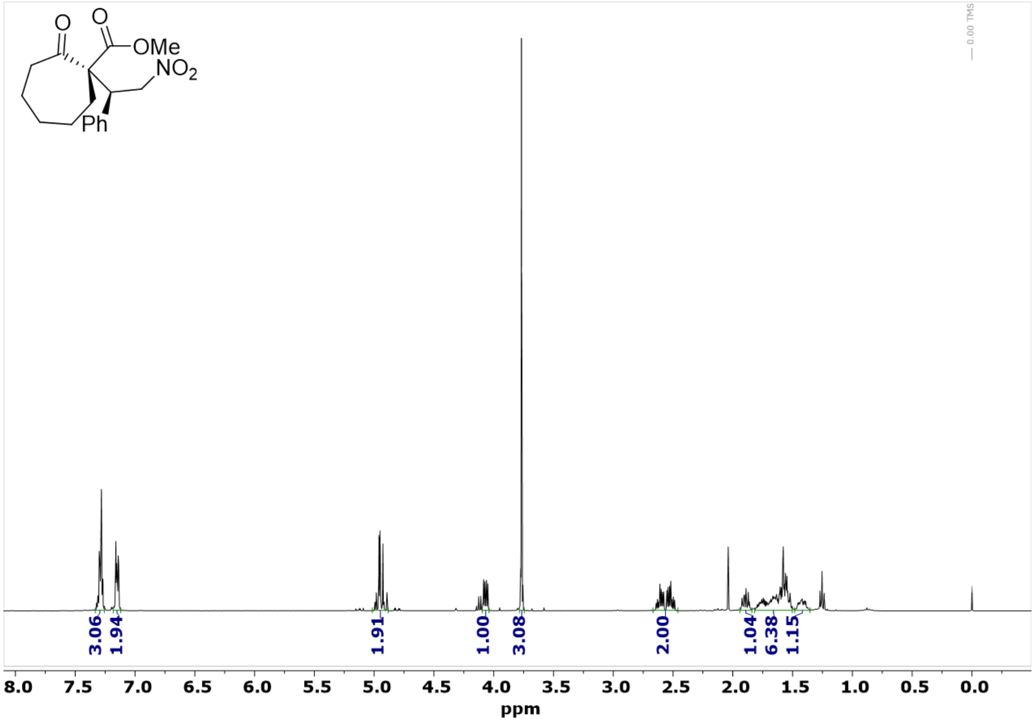

Figure S28: ^{1}H NMR, $CDCl_3$, 400 MHz (ppm) **5**

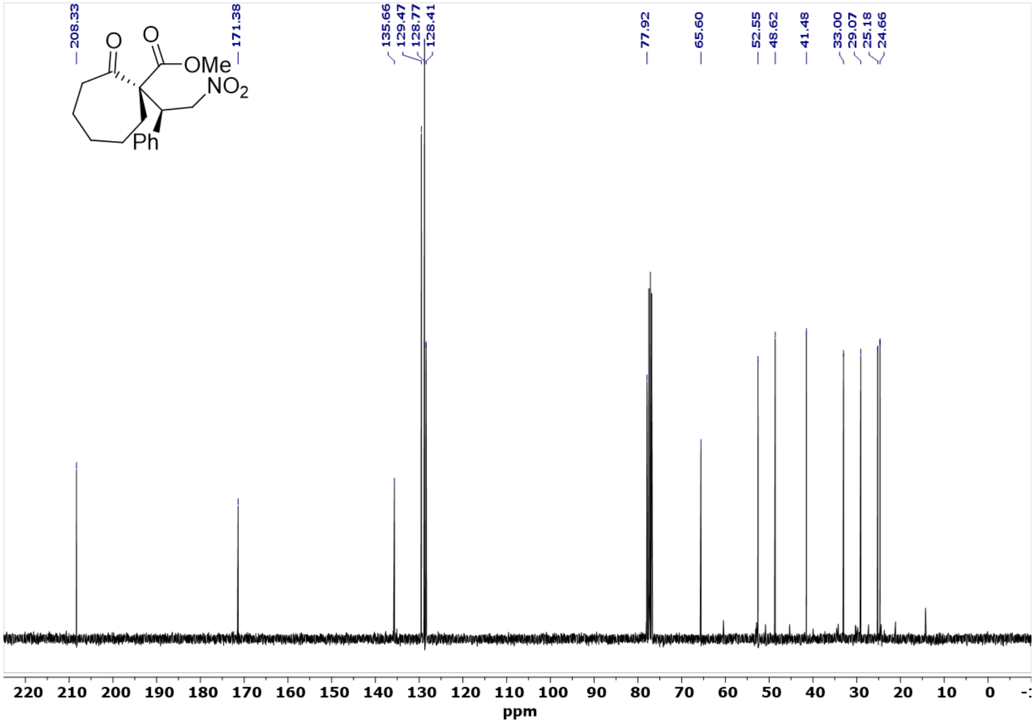

Figure S29: ^{13}C NMR, $CDCl_3$, 100 MHz (ppm) **5**

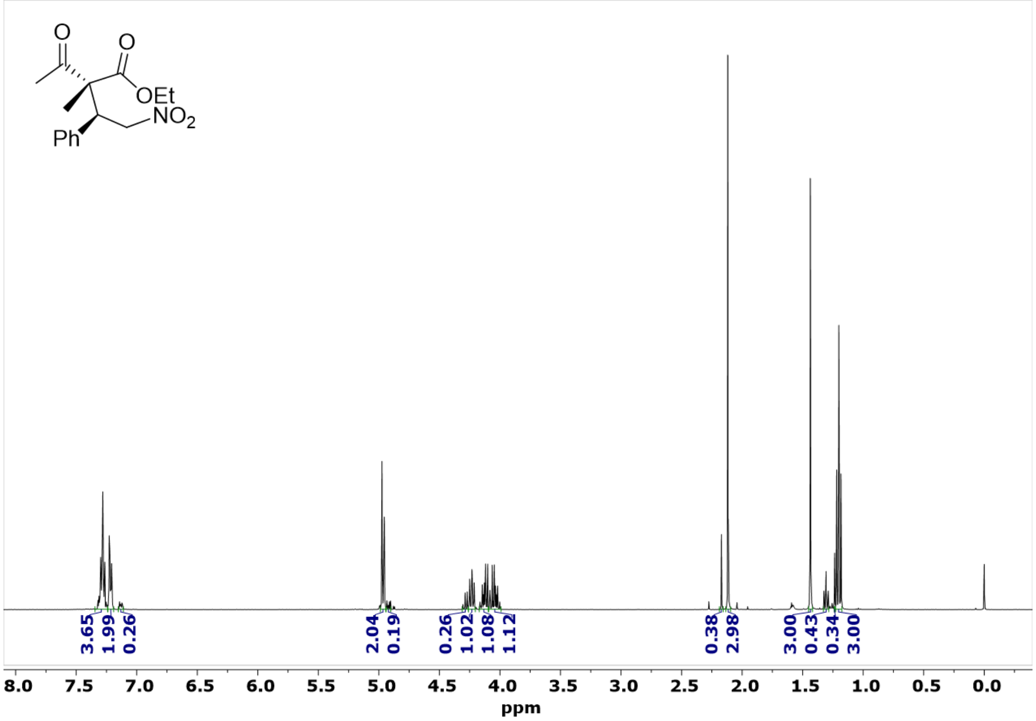

Figure S30: ^{1}H NMR, $CDCl_3$, 400 MHz (ppm) **6**

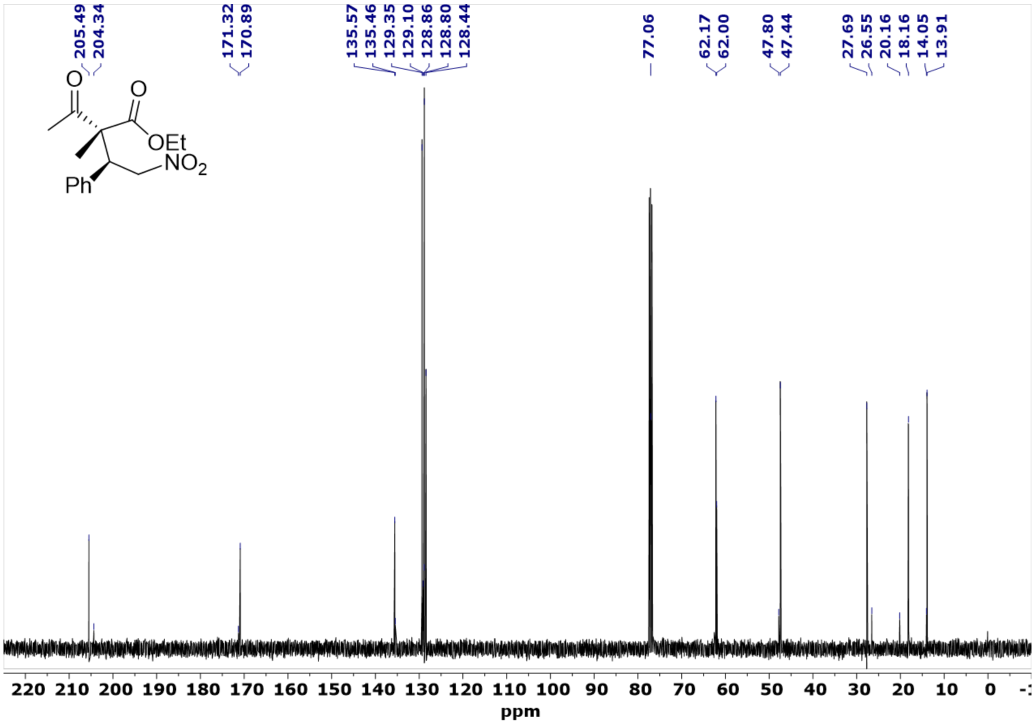

Figure S31: ^{13}C NMR, $CDCl_3$, 100 MHz (ppm) **6**

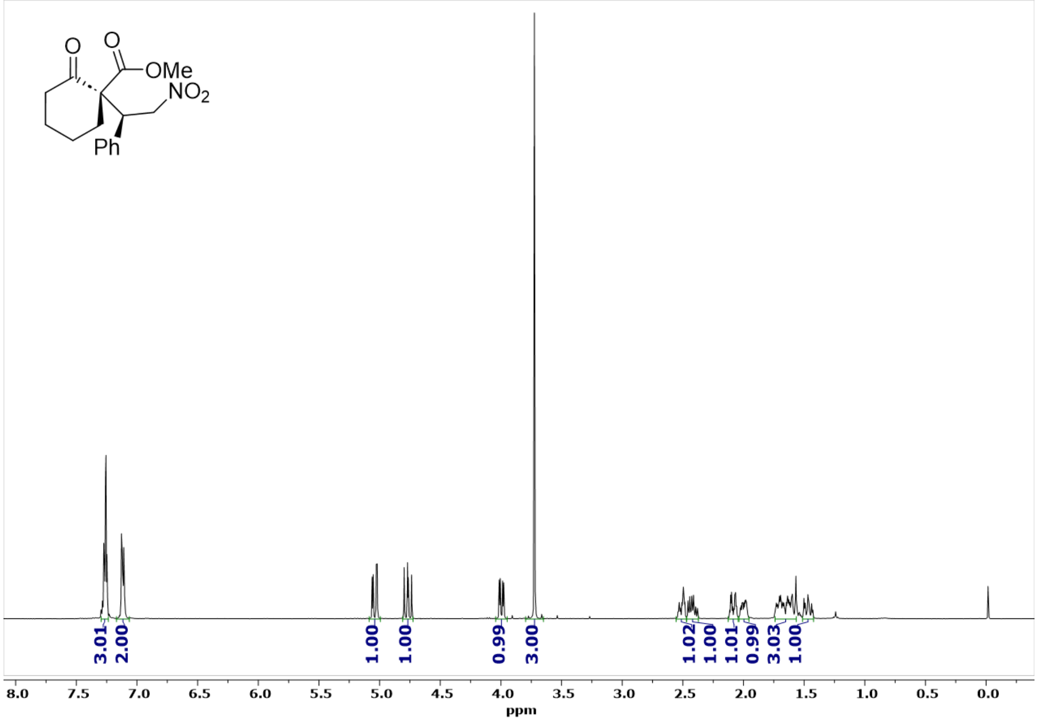

Figure S32: ^{1}H NMR, $CDCl_3$, 400 MHz (ppm) **7**

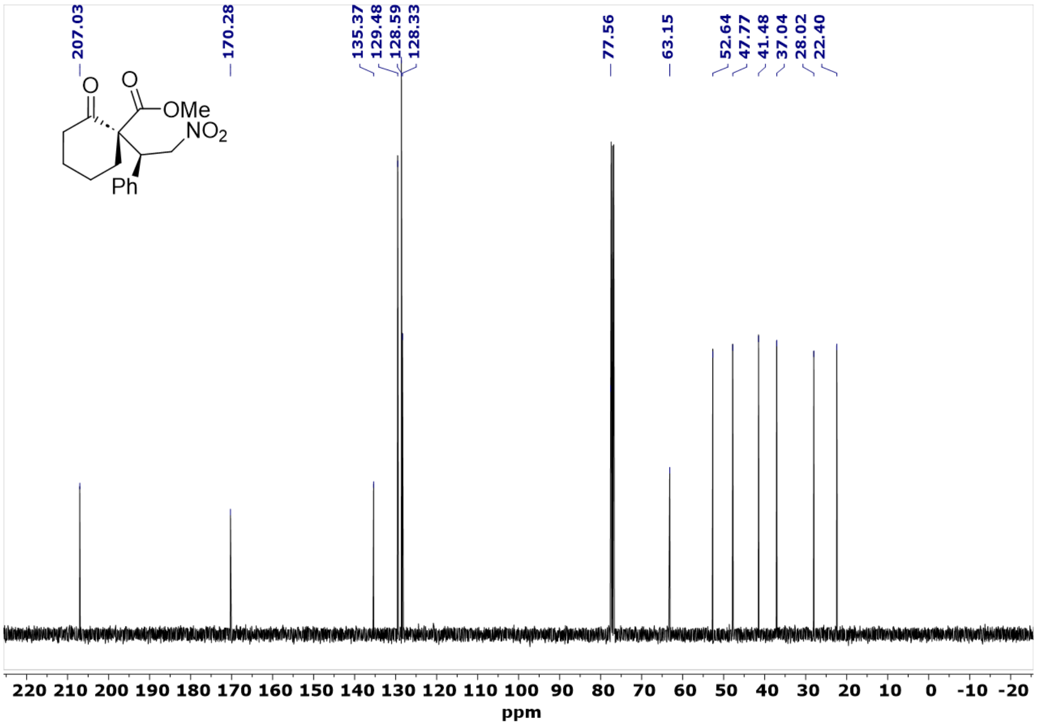

Figure S33: ^{13}C NMR, $CDCl_3$, 100 MHz (ppm) **7**

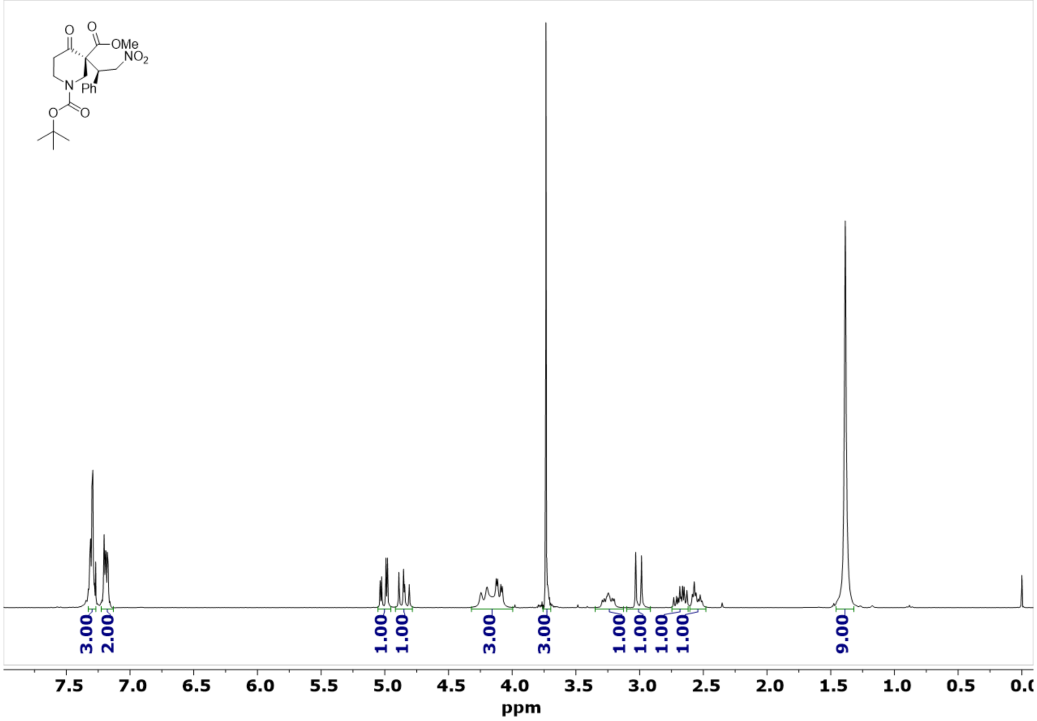

Figure S34: ^{1}H NMR, $CDCl_3$, 400 MHz (ppm) **8**

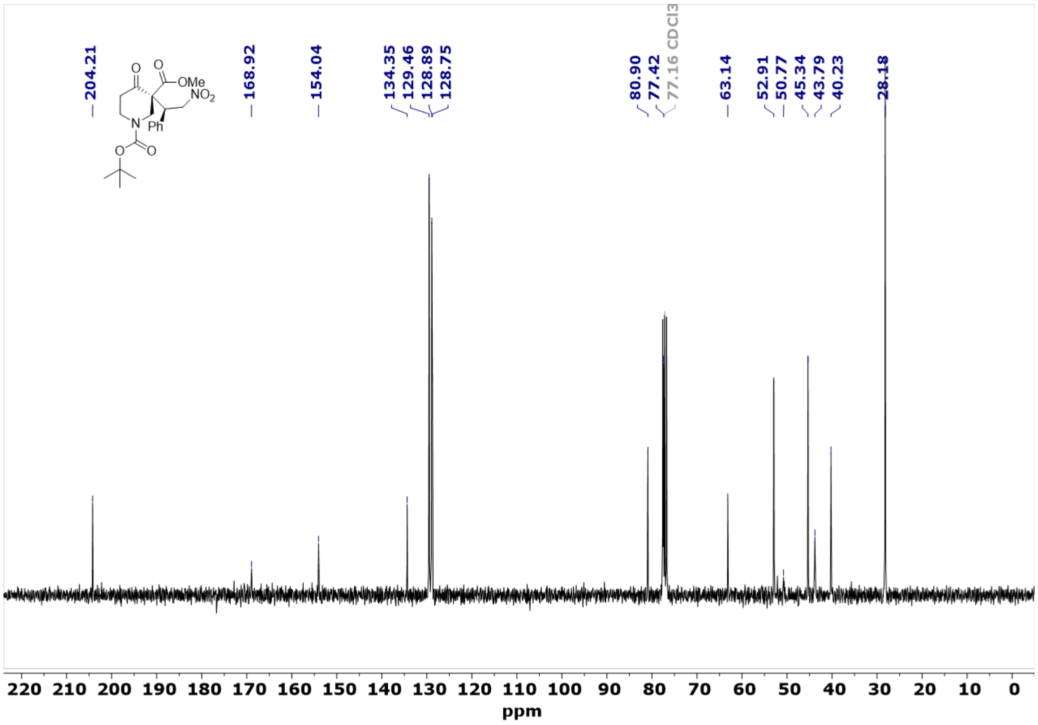

Figure S35: ^{13}C NMR, $CDCl_3$, 100 MHz (ppm) **8**

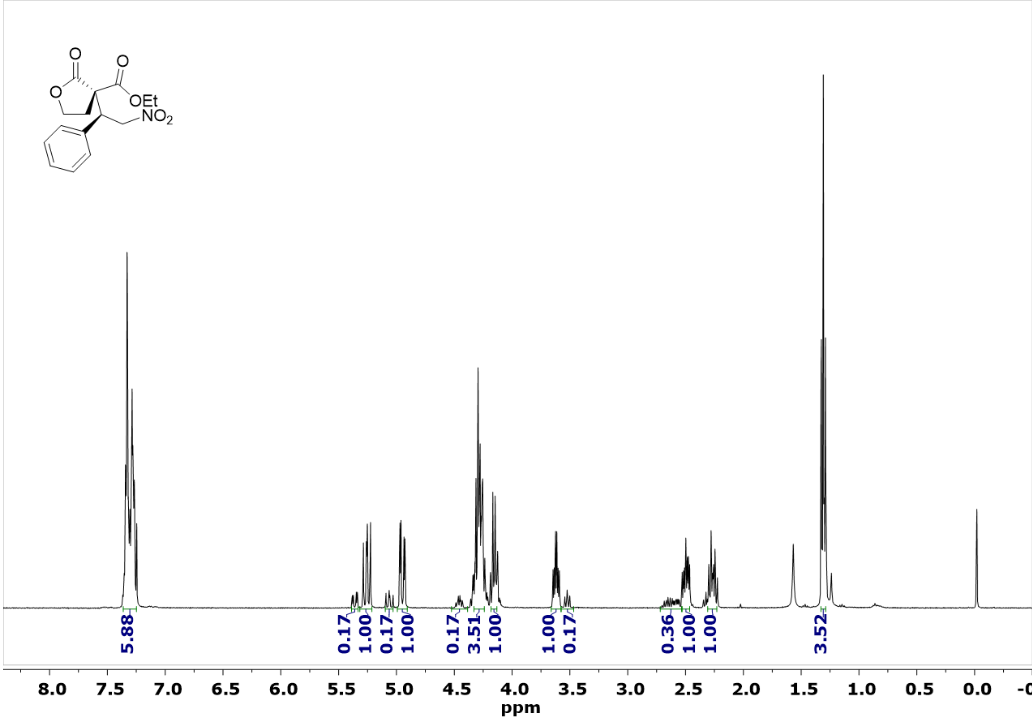

Figure S36: ^{1}H NMR, $CDCl_3$, 400 MHz (ppm) **9**

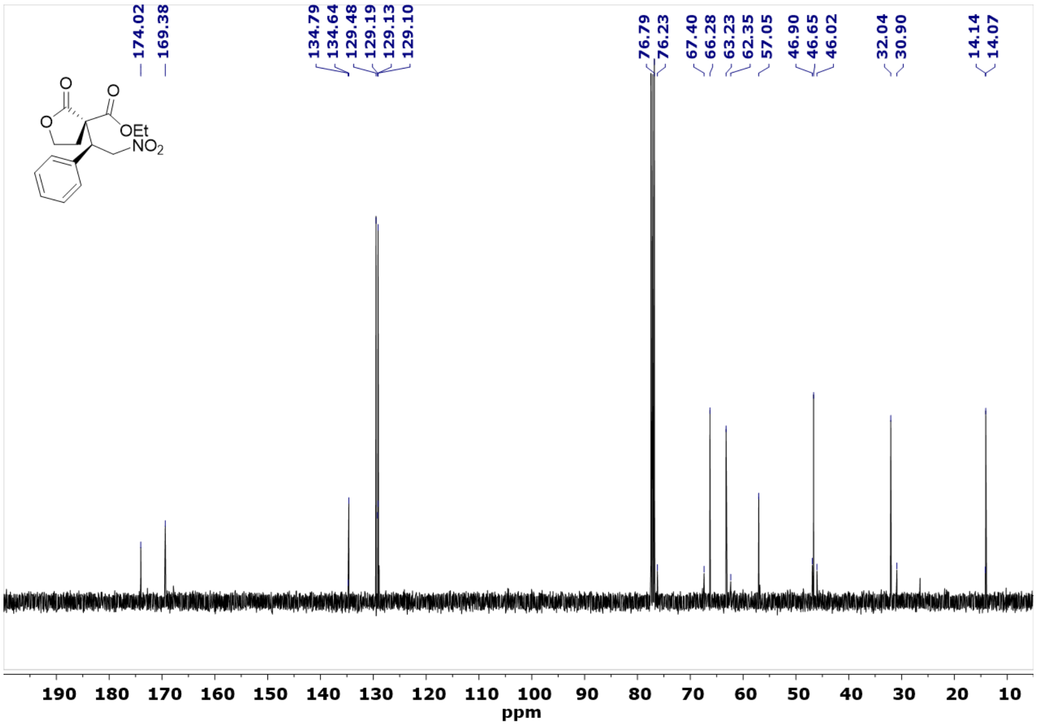

Figure S37: ^{13}C NMR, $CDCl_3$, 100 MHz (ppm) **9**

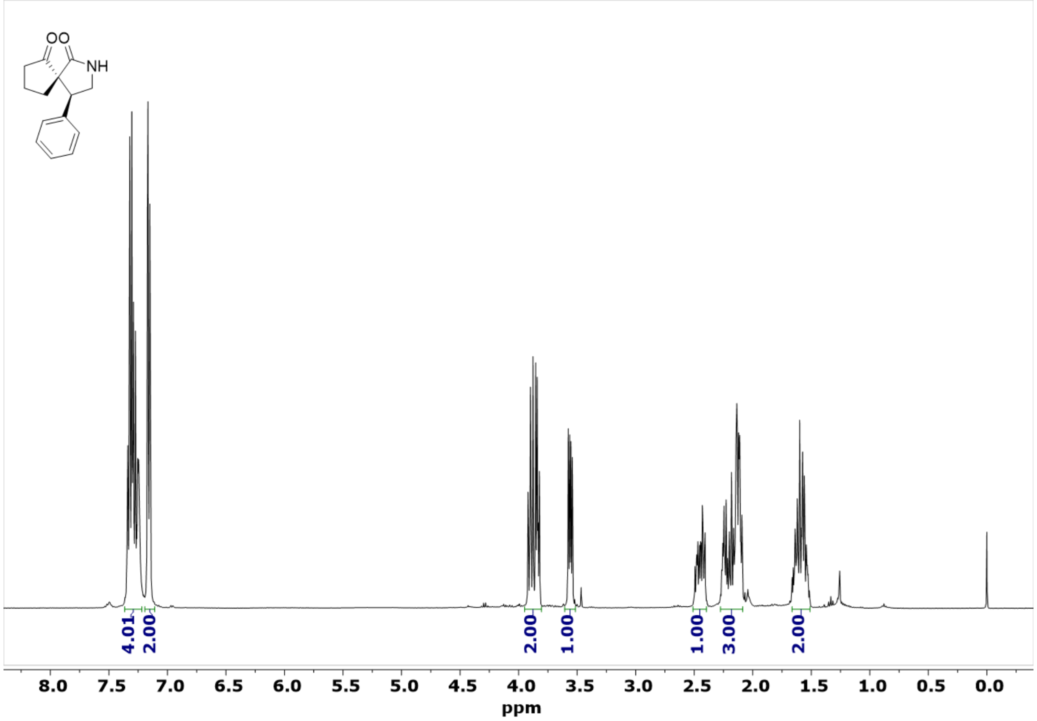

Figure S38: ^{1}H NMR, $CDCl_3$, 400 MHz (ppm) **10a**

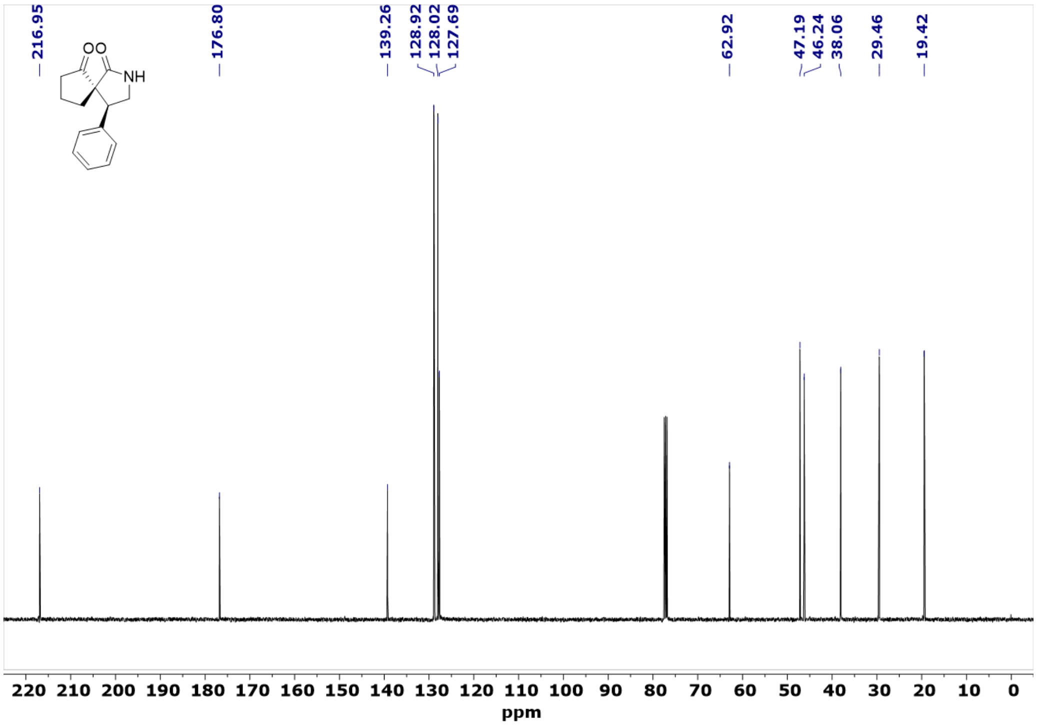

Figure S39: ^{13}C NMR, $CDCl_3$, 100 MHz (ppm) **10a**

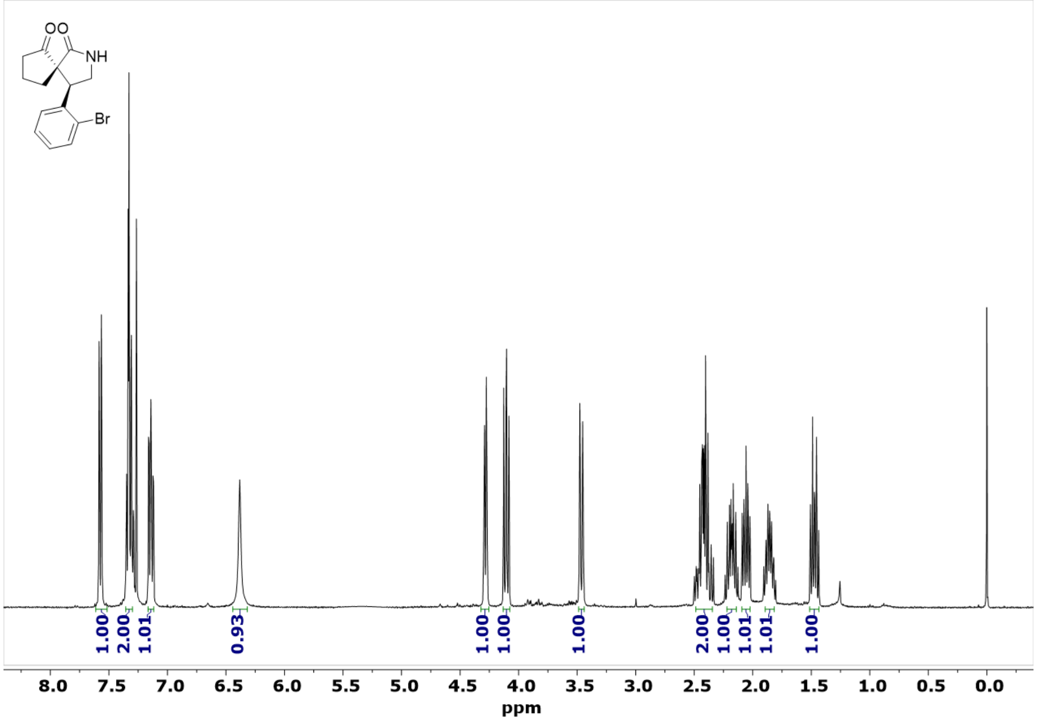

Figure S40: ^{1}H NMR, $CDCl_3$, 400 MHz (ppm) **10b**

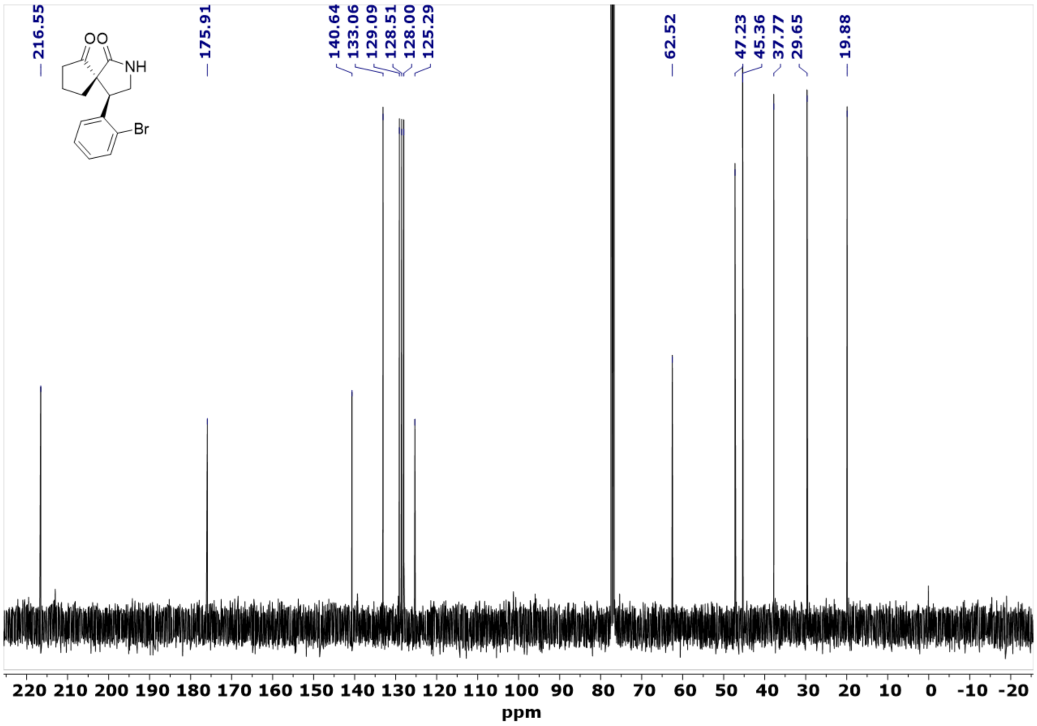

Figure S41: ^{13}C NMR, $CDCl_3$, 100 MHz (ppm) **10b**

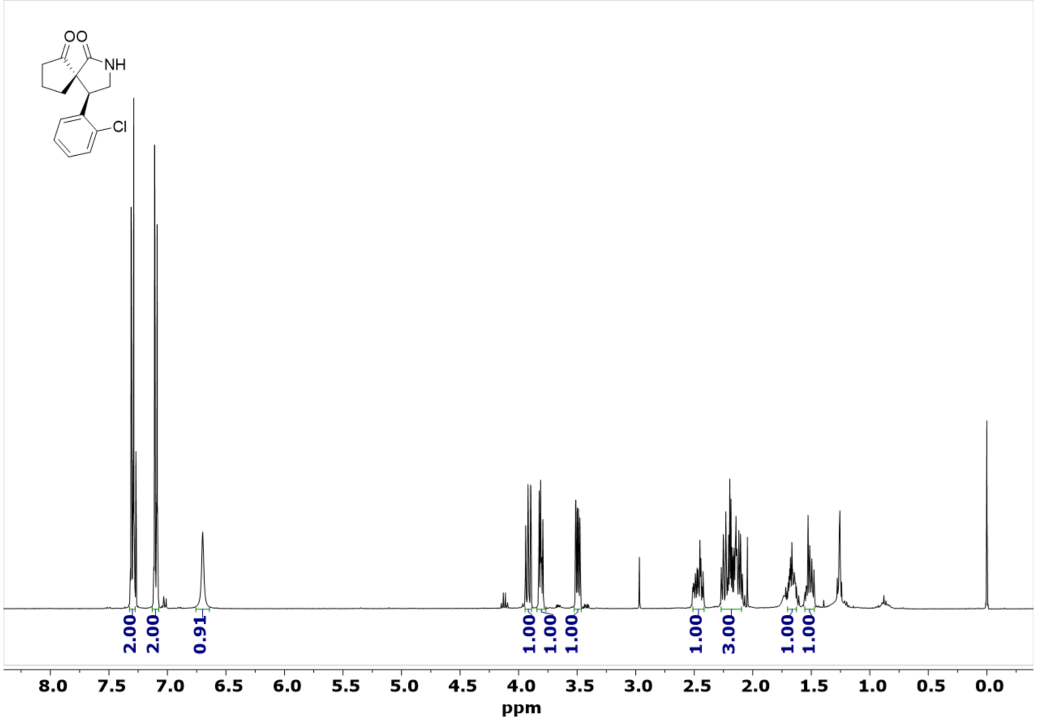

Figure S42: ^{1}H NMR, $CDCl_3$, 400 MHz (ppm) **10c**

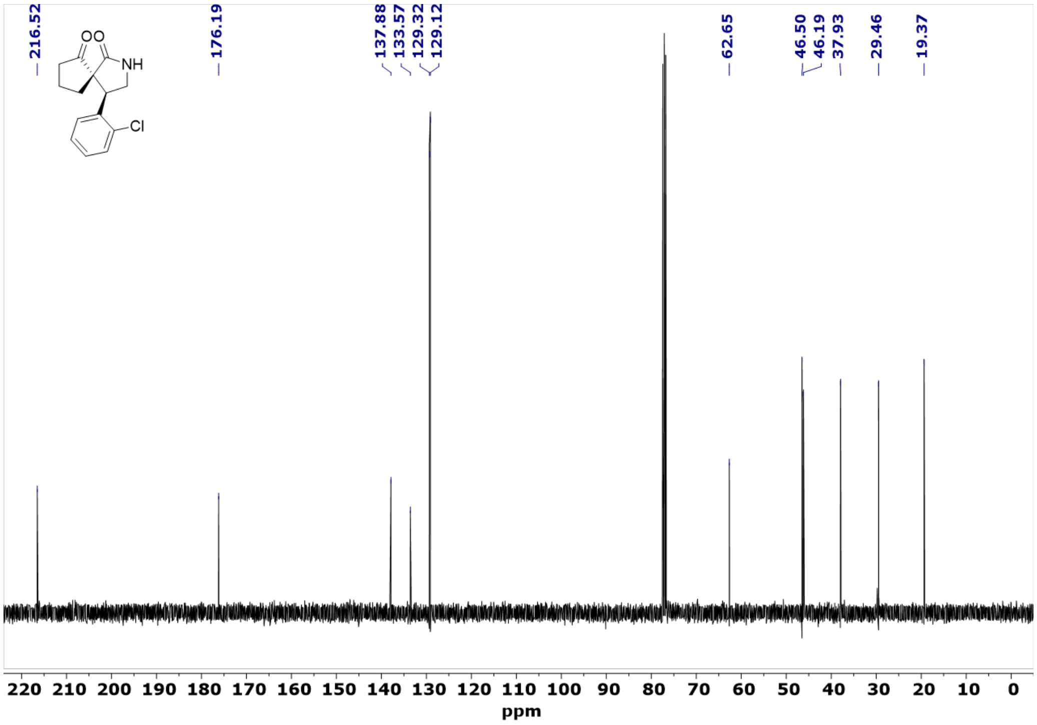

Figure S43: ^{13}C NMR, $CDCl_3$, 100 MHz (ppm) **10c**

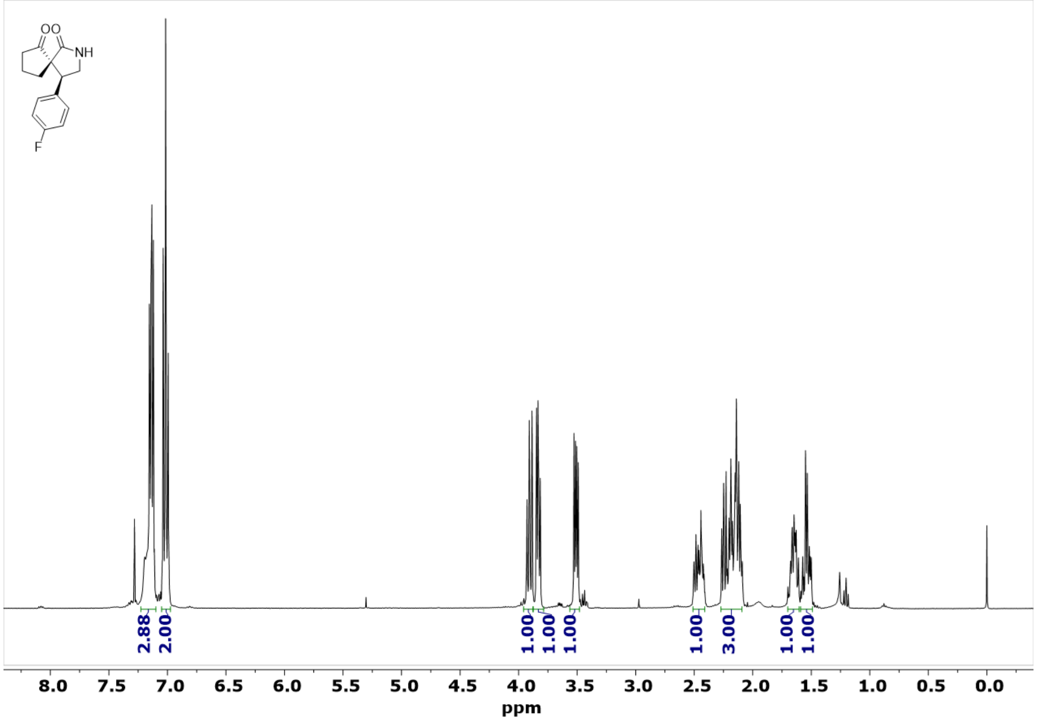

Figure S44: ^{1}H NMR, $CDCl_3$, 400 MHz (ppm) **10d**

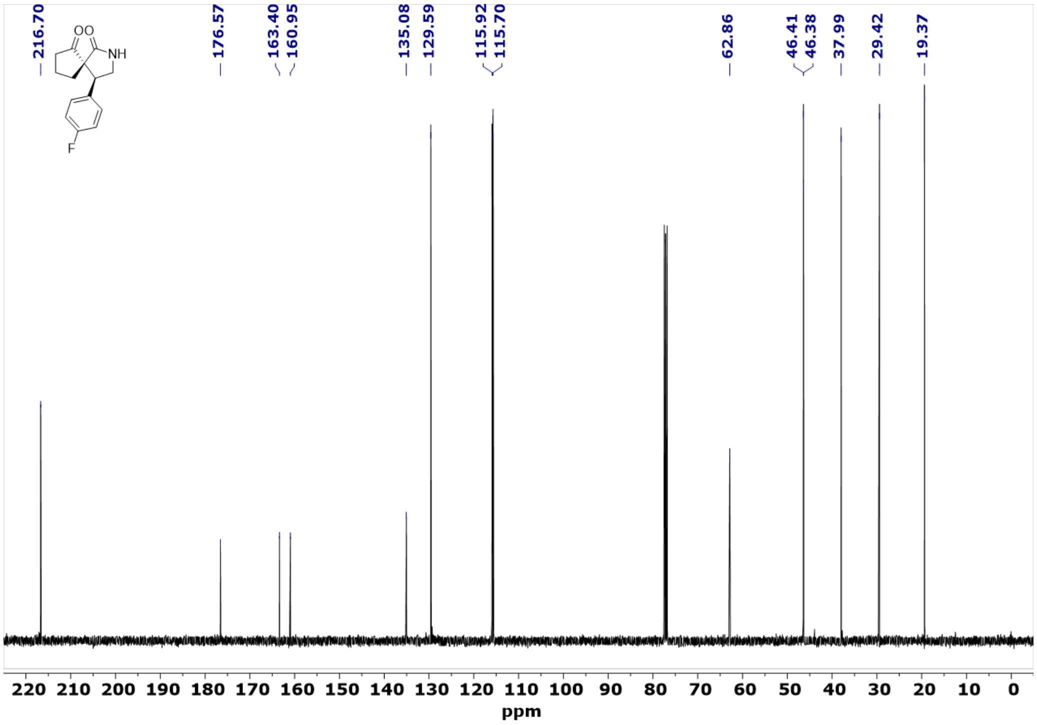

Figure S45: ^{13}C NMR, $CDCl_3$, 100 MHz (ppm) **10d**

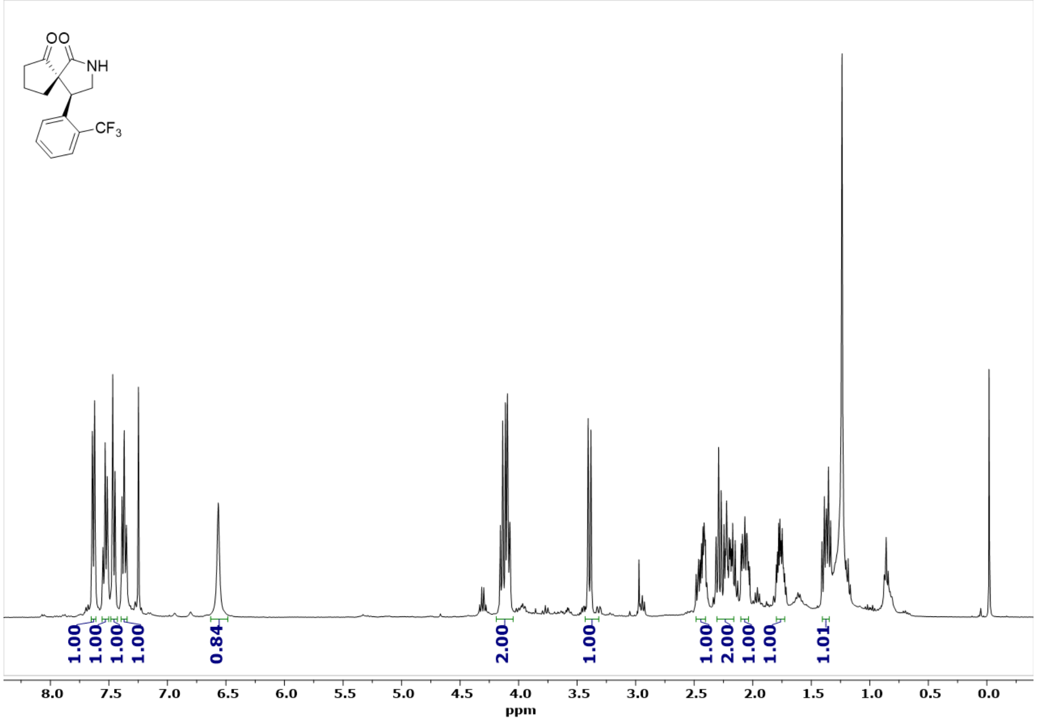

Figure S46: ^{1}H NMR, $CDCl_3$, 400 MHz (ppm) **10e**

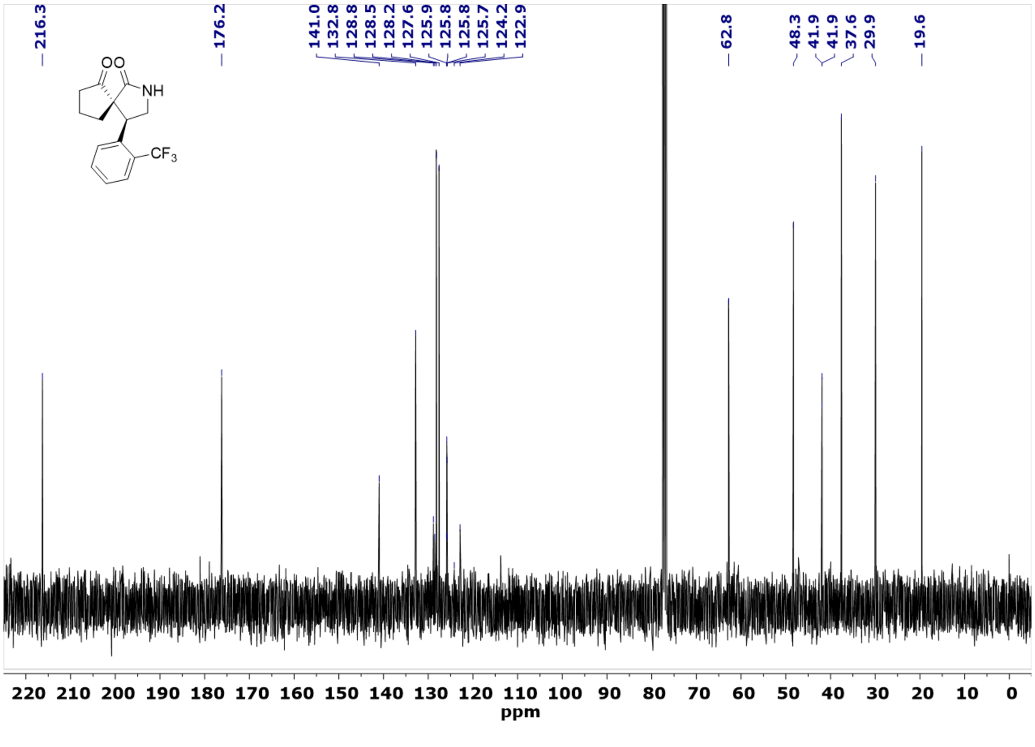

Figure S47: ^{13}C NMR, $CDCl_3$, 100 MHz (ppm) **10e**

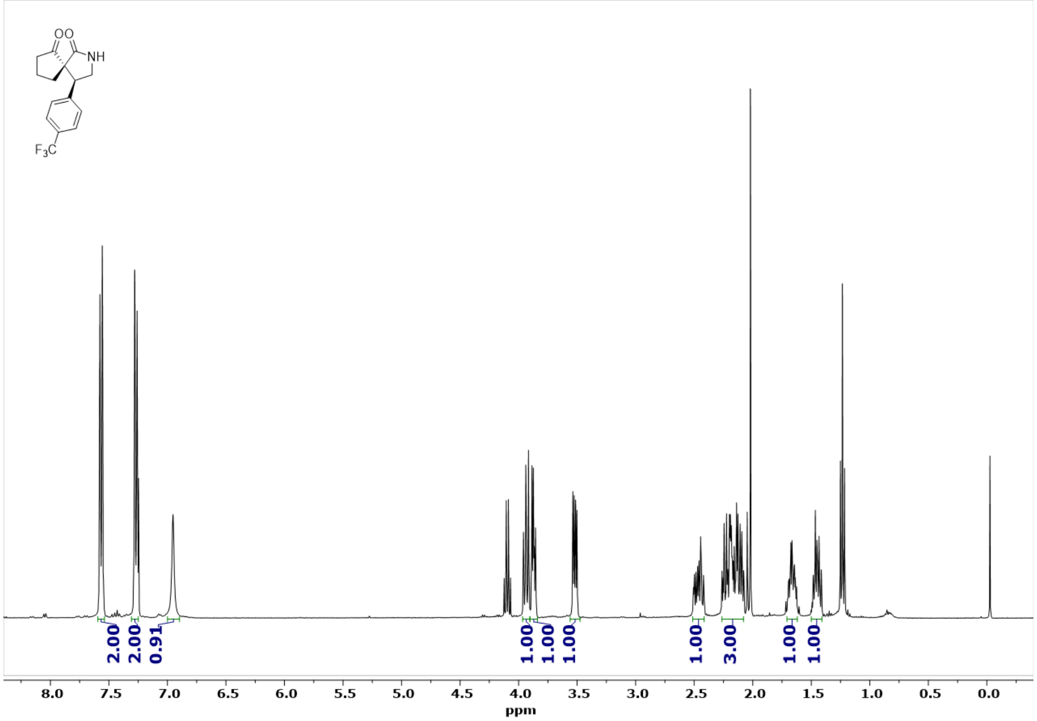

Figure S48: ^{1}H NMR, $CDCl_3$, 400 MHz (ppm) **10f**

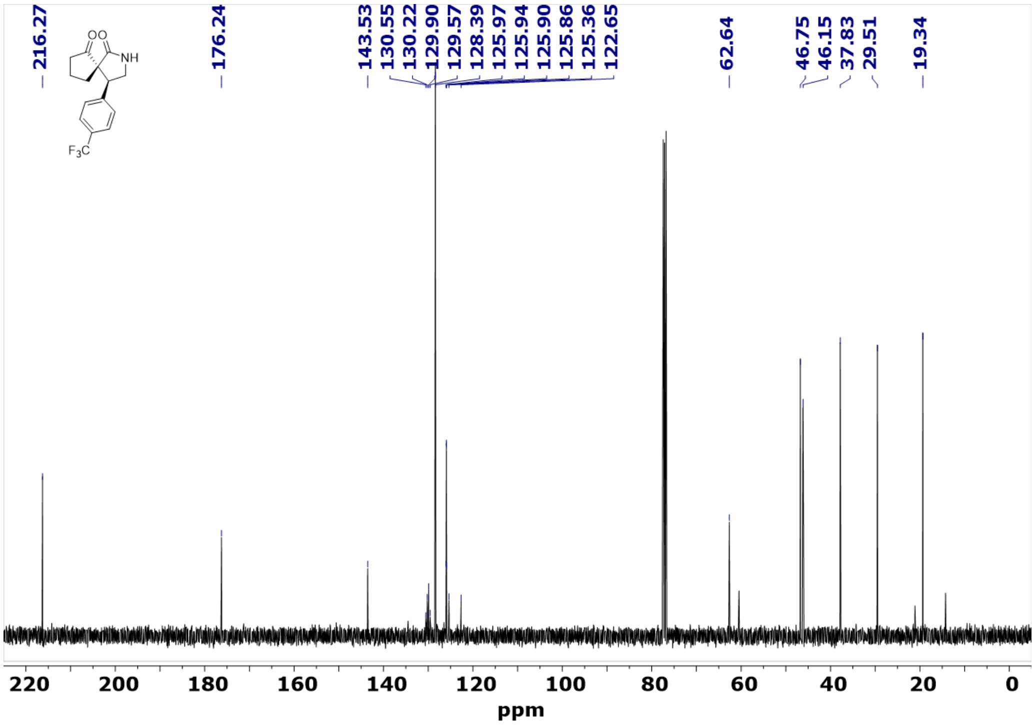

Figure S49: ^{13}C NMR, $CDCl_3$, 100 MHz (ppm) **10f**

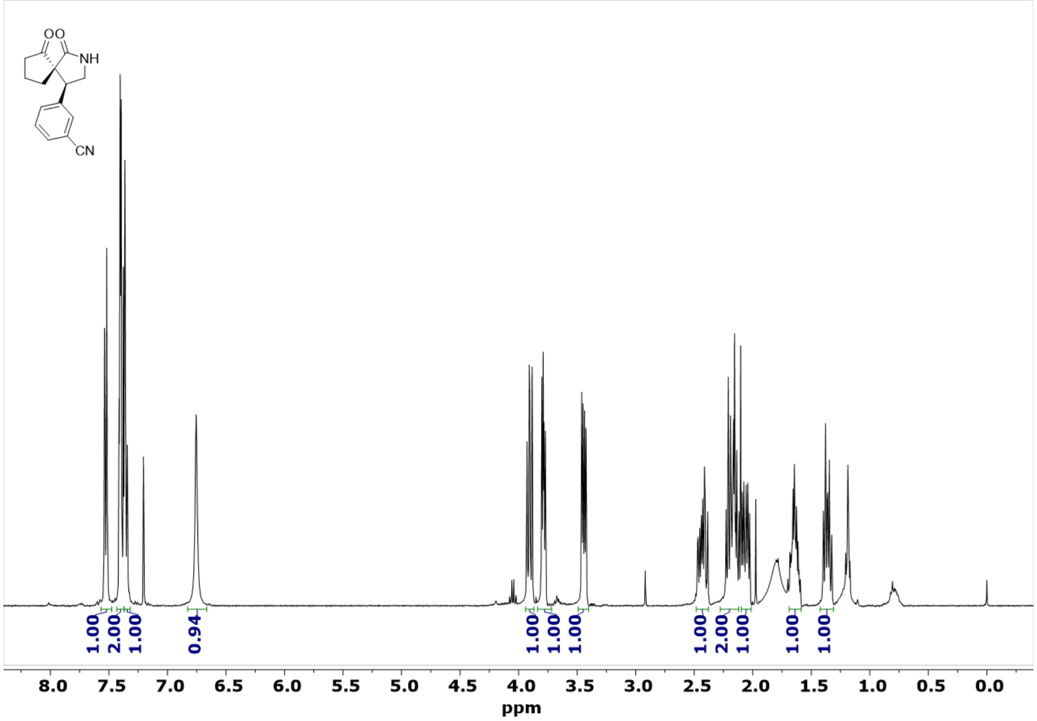

Figure S50: ^{1}H NMR, $CDCl_3$, 400 MHz (ppm) **10g**

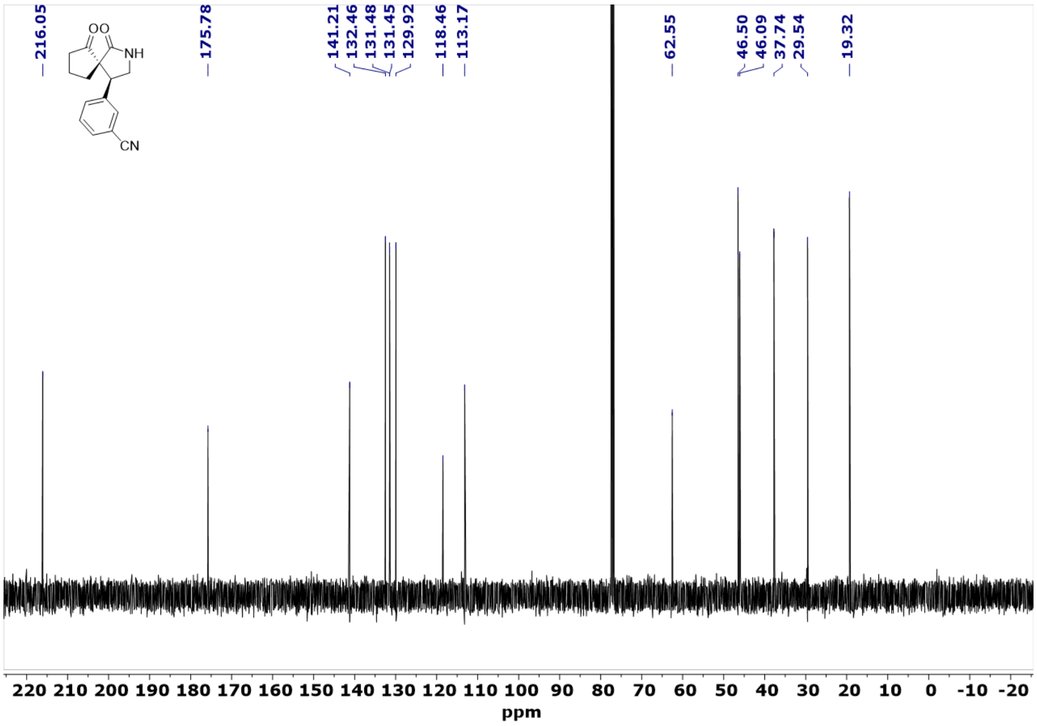

Figure S51: ^{13}C NMR, $CDCl_3$, 100 MHz (ppm) **10g**

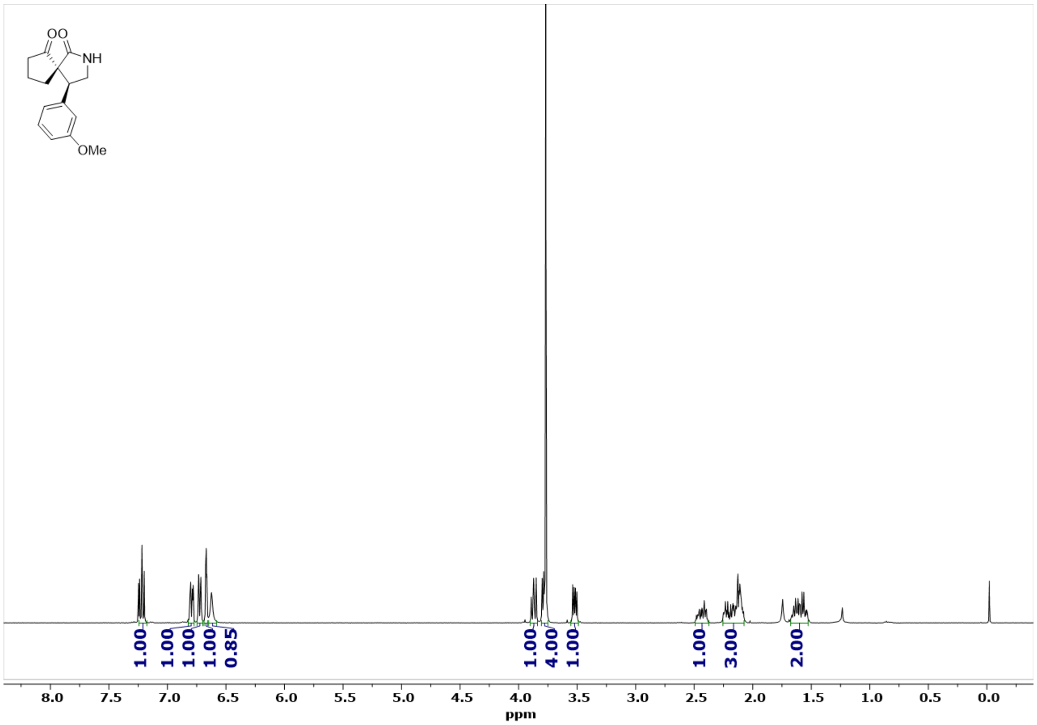

Figure S52: ^{1}H NMR, $CDCl_3$, 400 MHz (ppm) **10h**

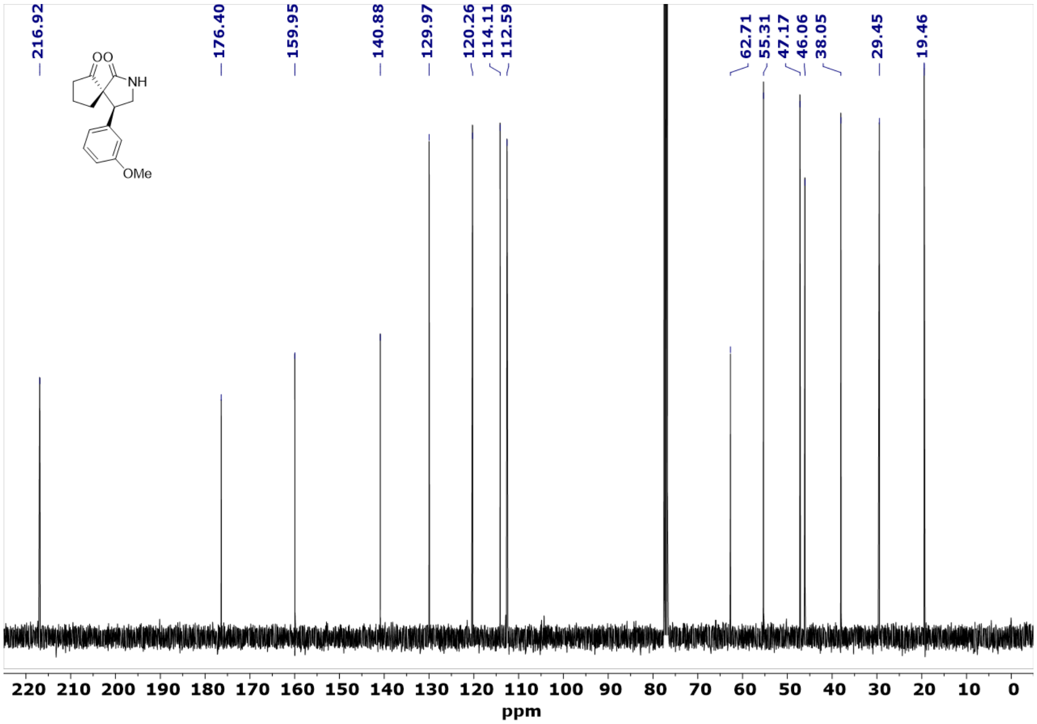

Figure S53: ^{13}C NMR, $CDCl_3$, 100 MHz (ppm) **10h**

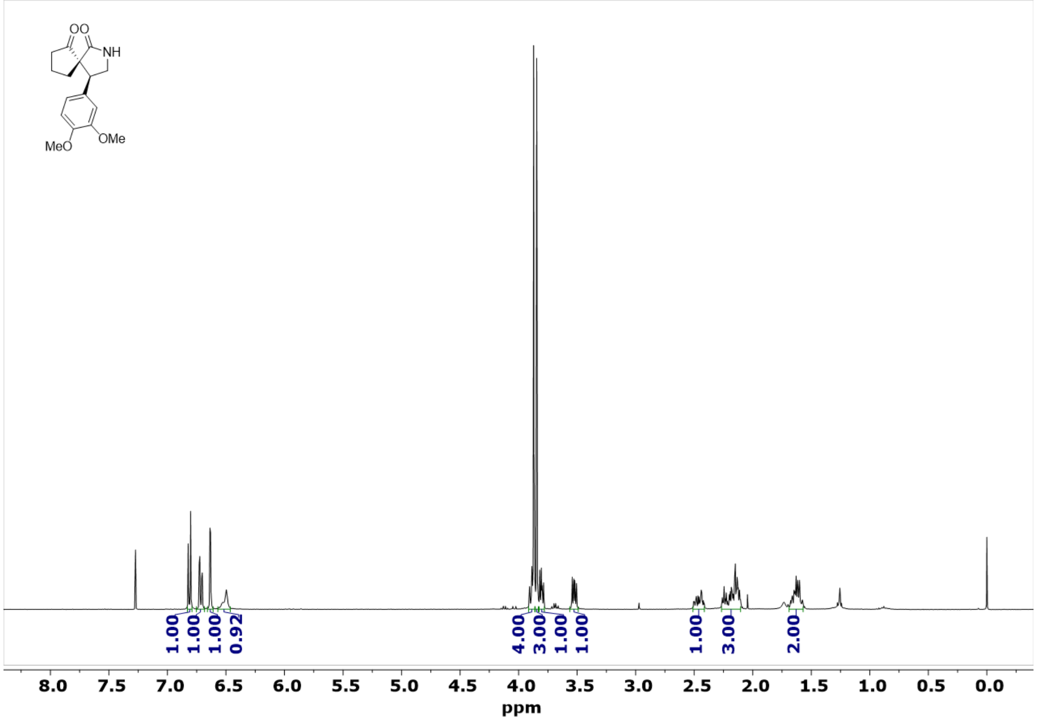

Figure S54: ^{1}H NMR, $CDCl_3$, 400 MHz (ppm) **10i**

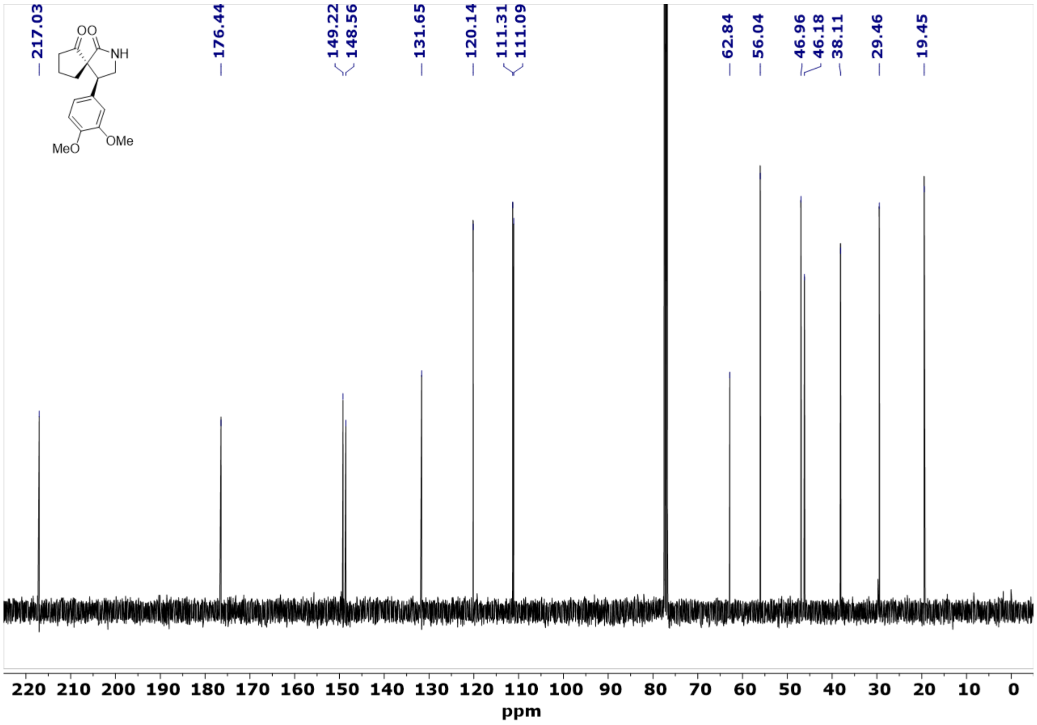

Figure S55: ^{13}C NMR, $CDCl_3$, 100 MHz (ppm) **10i**

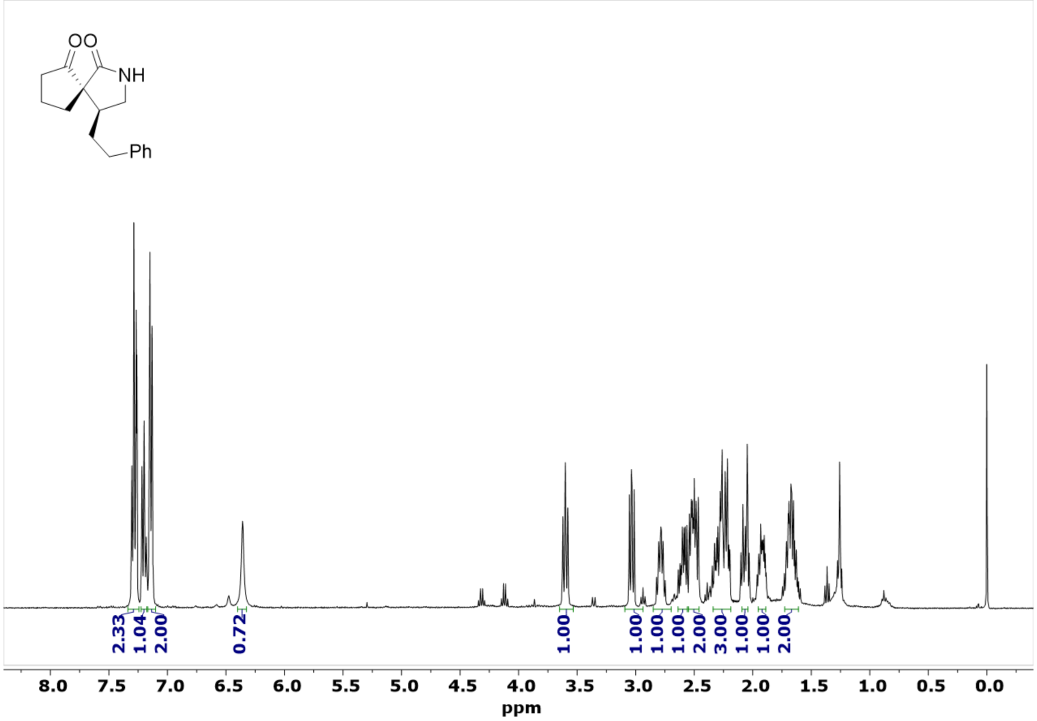

Figure S56: ^{1}H NMR, $CDCl_3$, 400 MHz (ppm) **10j**

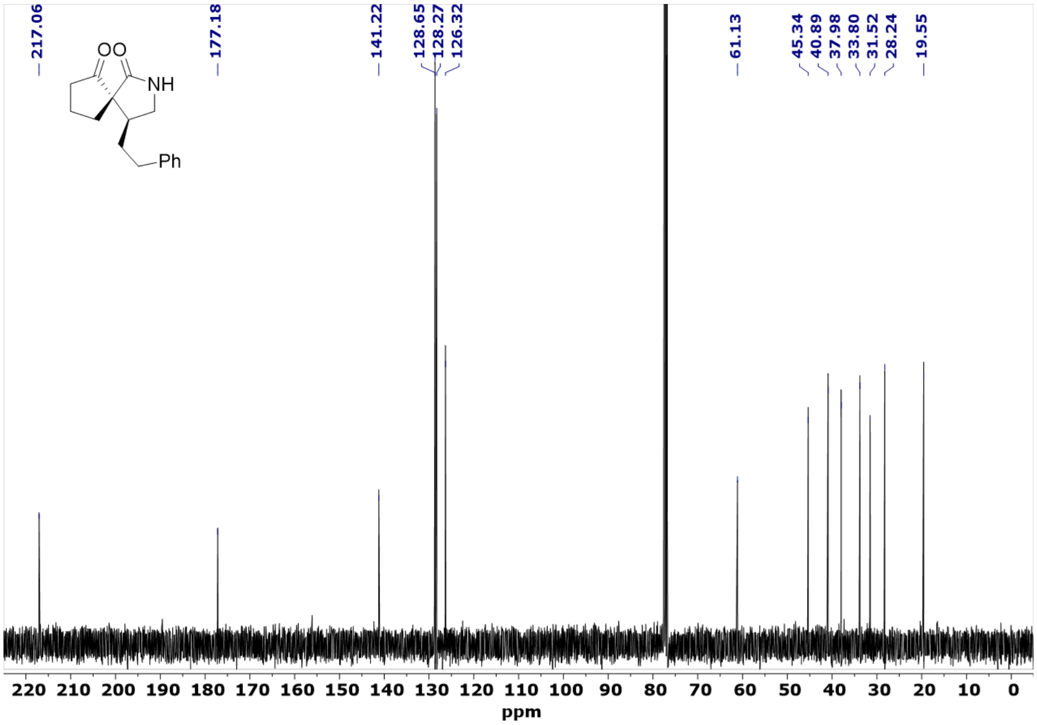

Figure S57: ^{13}C NMR, $CDCl_3$, 100 MHz (ppm) **10j**

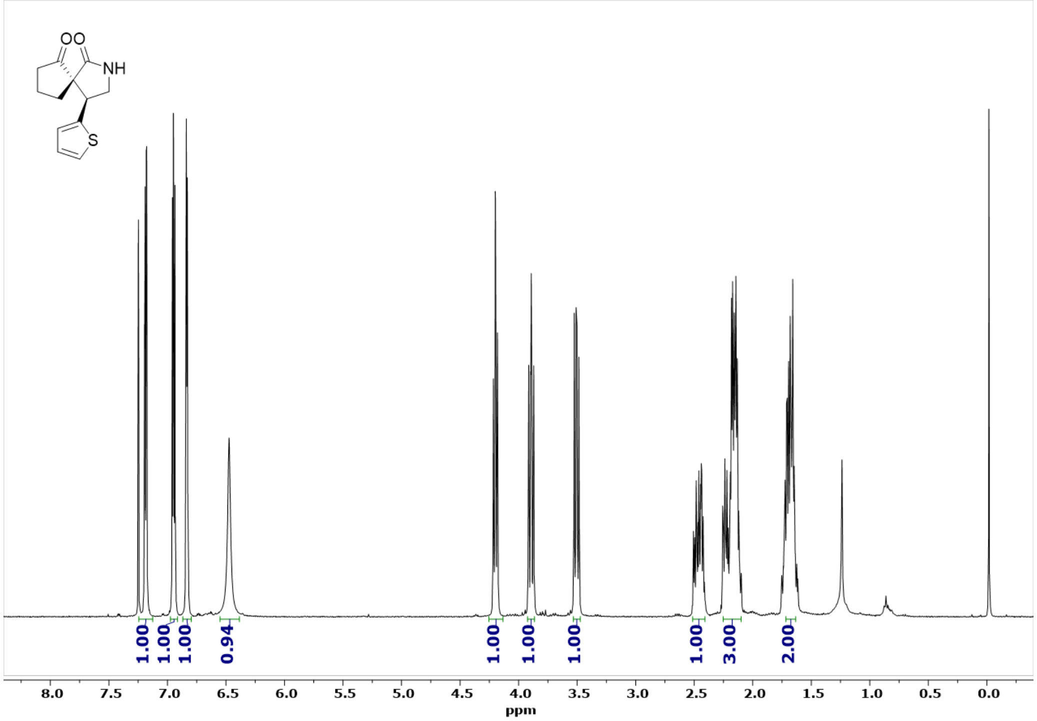

Figure S58: ^{1}H NMR, $CDCl_3$, 400 MHz (ppm) **10k**

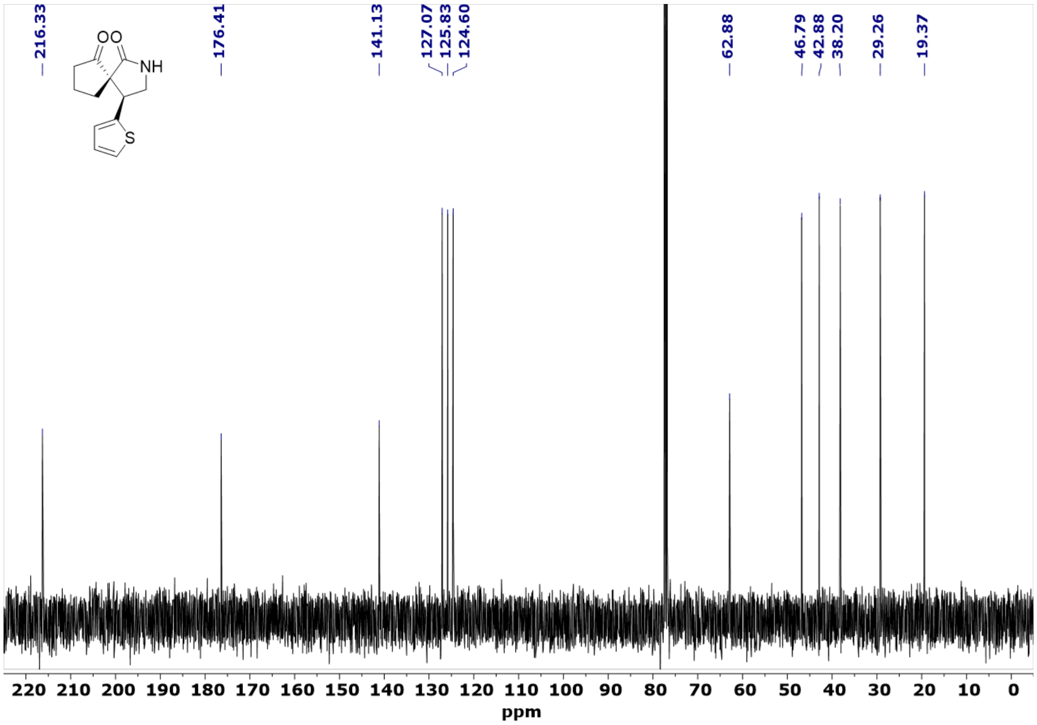

Figure S59: ^{13}C NMR, $CDCl_3$, 100 MHz (ppm) **10k**

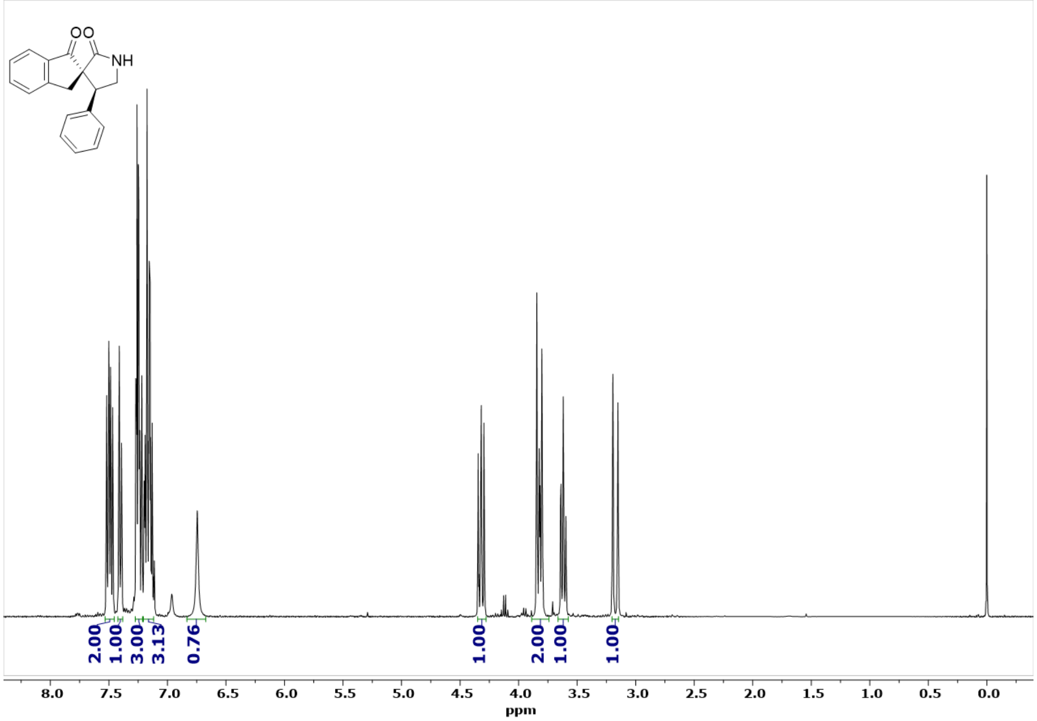

Figure S60: ^{1}H NMR, $CDCl_3$, 400 MHz (ppm) **11**

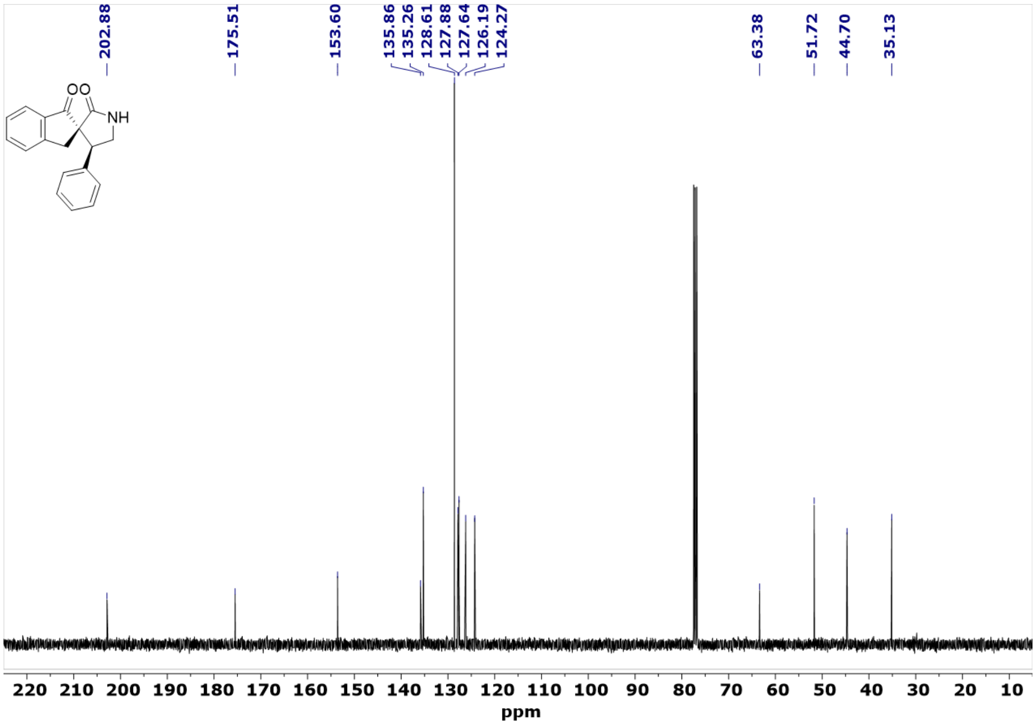

Figure S61: ^{13}C NMR, $CDCl_3$, 100 MHz (ppm) **11**

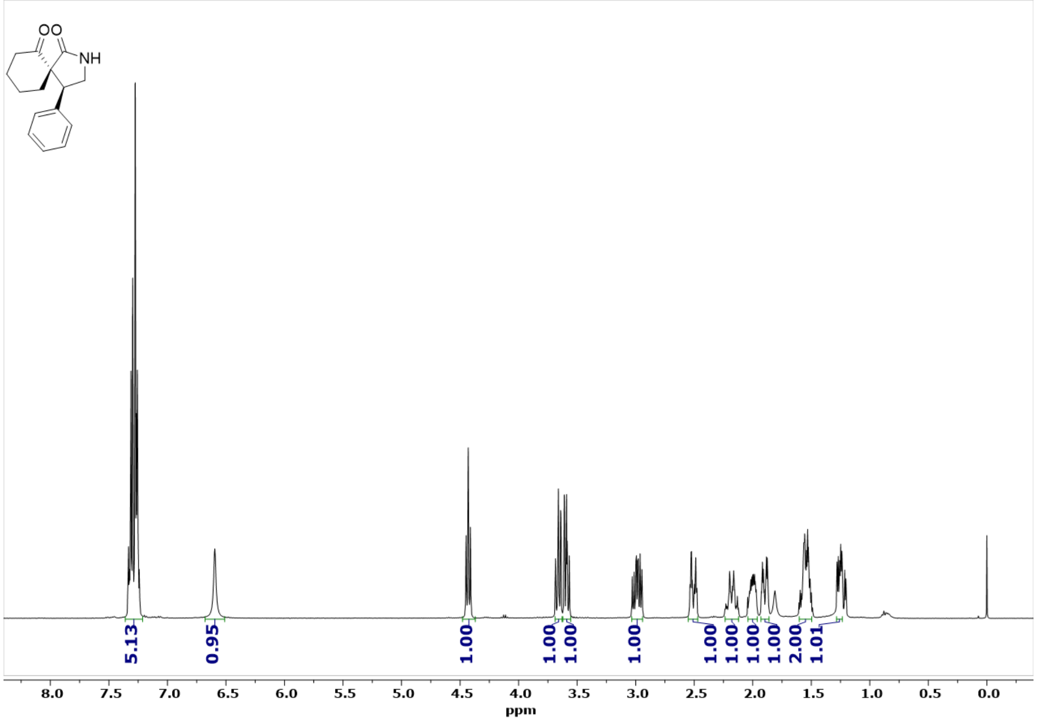

Figure S62: ^{1}H NMR, $CDCl_3$, 400 MHz (ppm) **12**

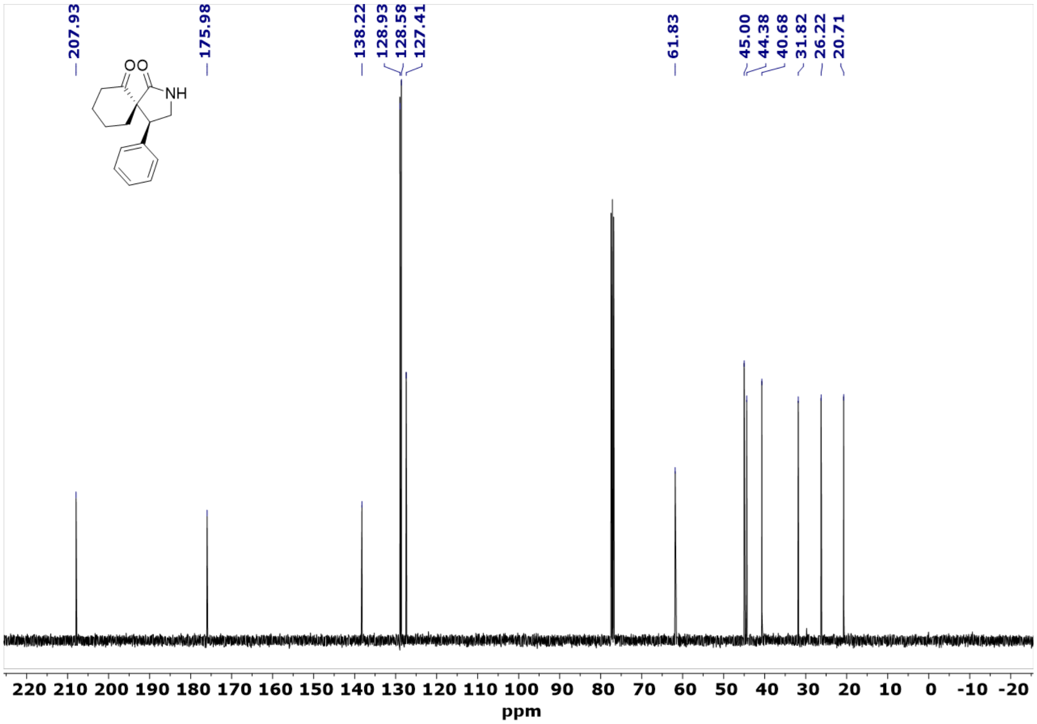

Figure S63: ^{13}C NMR, $CDCl_3$, 100 MHz (ppm) **12**

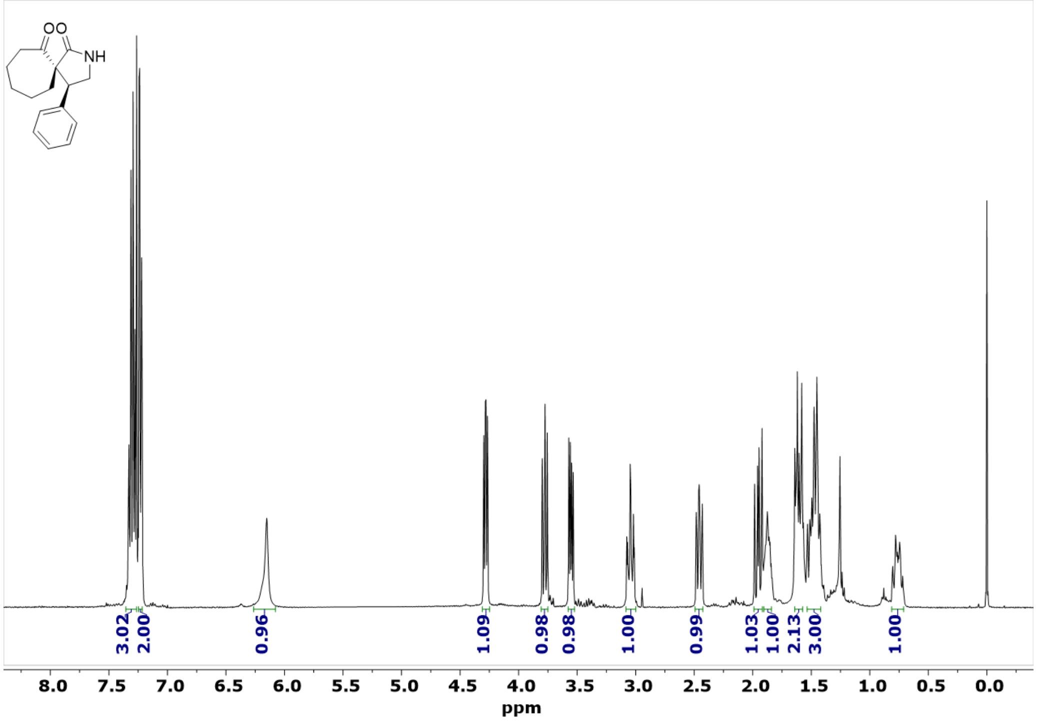

Figure S64: ^{1}H NMR, $CDCl_3$, 400 MHz (ppm) **13**

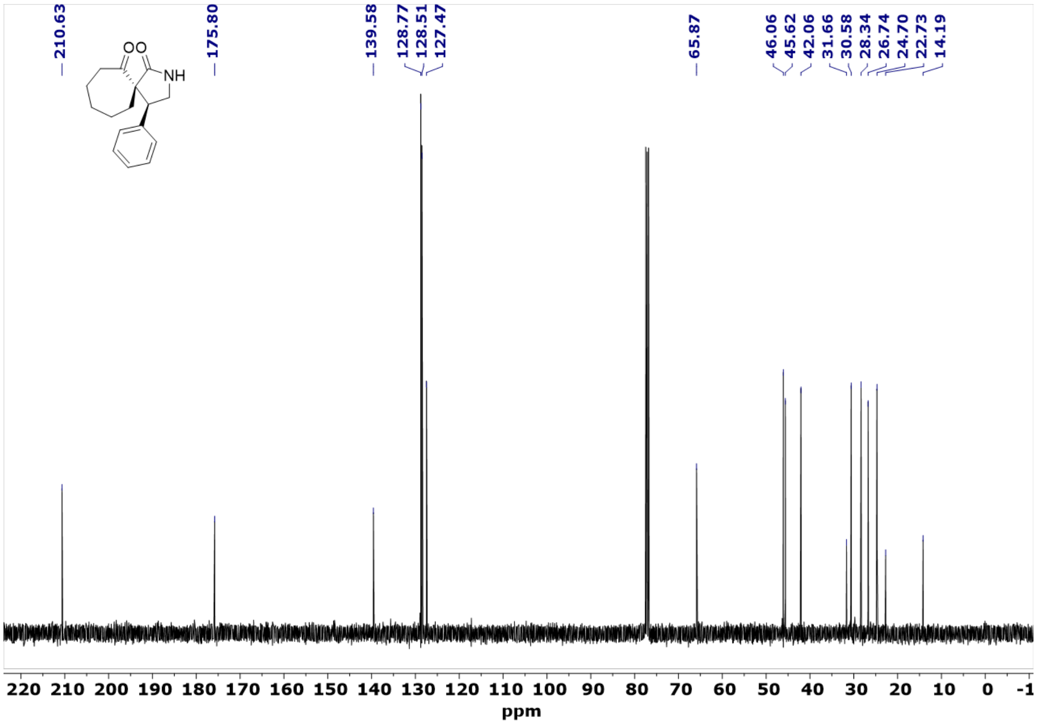

Figure S65: ^{13}C NMR, $CDCl_3$, 100 MHz (ppm) **13**

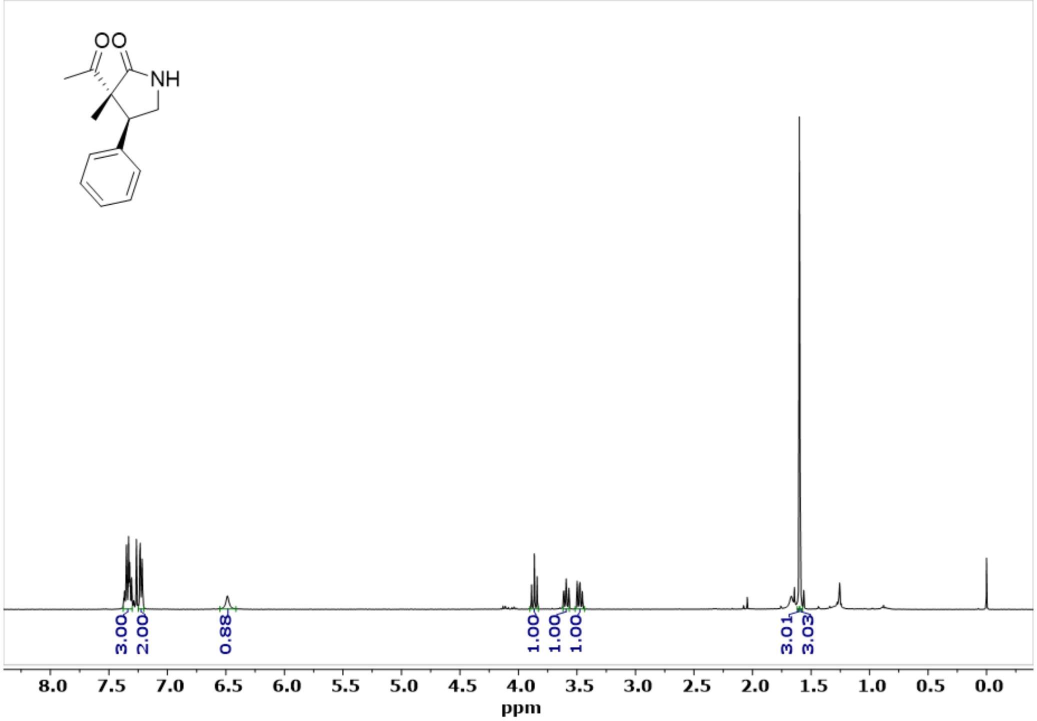

Figure S66: ^{1}H NMR, $CDCl_3$, 400 MHz (ppm) **14**

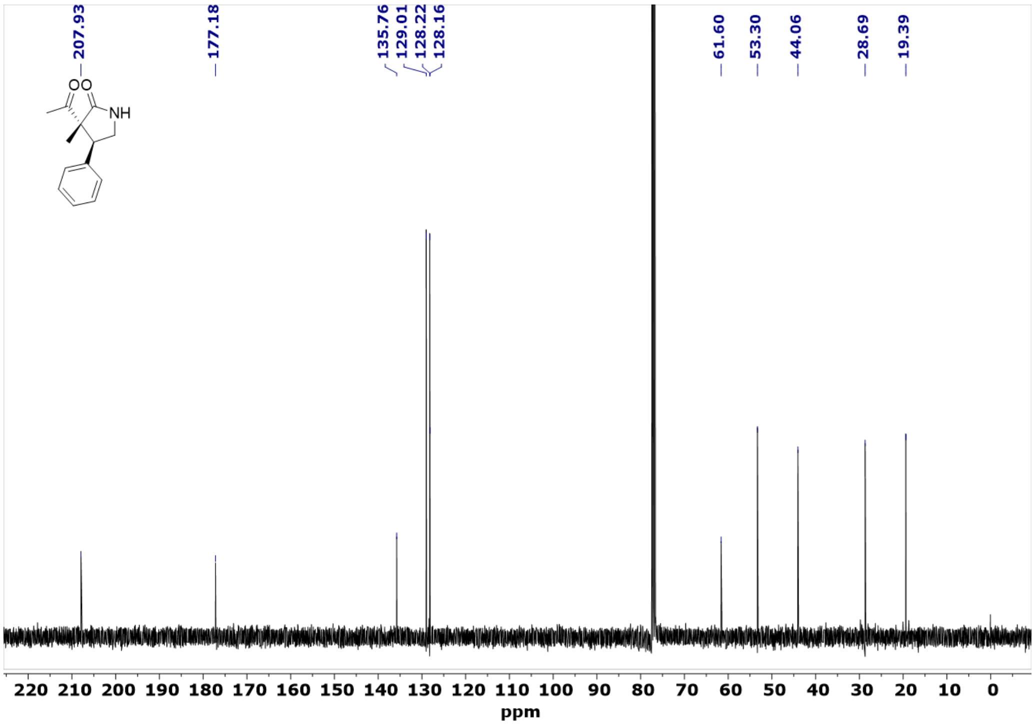

Figure S67: ^{13}C NMR, $CDCl_3$, 100 MHz (ppm) **14**

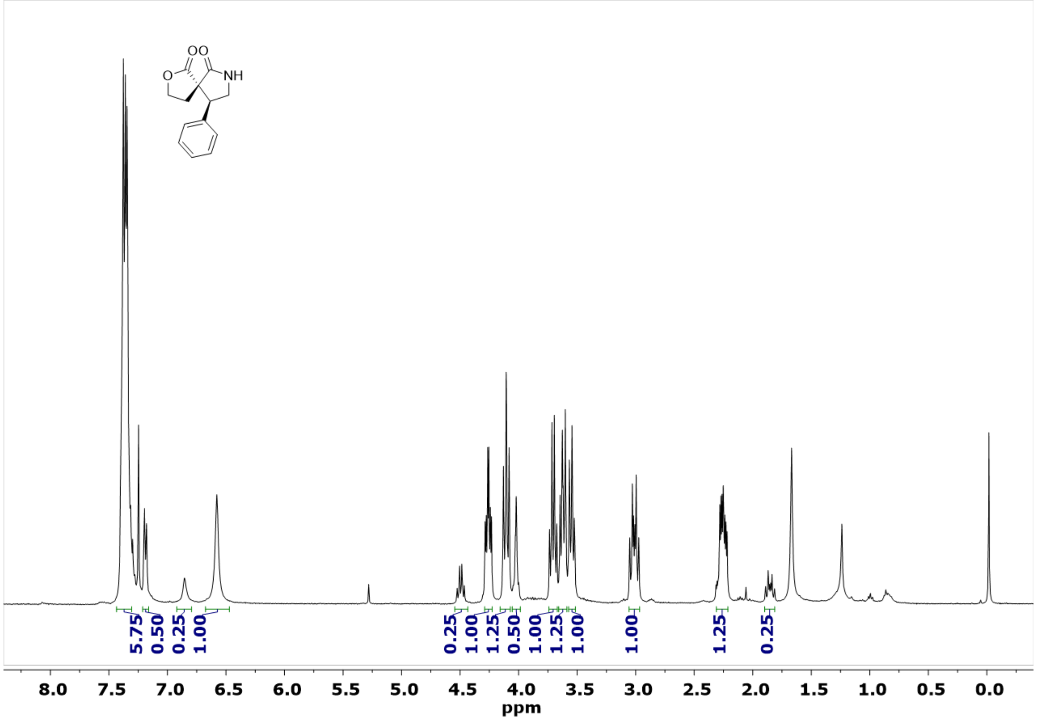

Figure S68: ^{1}H NMR, $CDCl_3$, 400 MHz (ppm) **15**

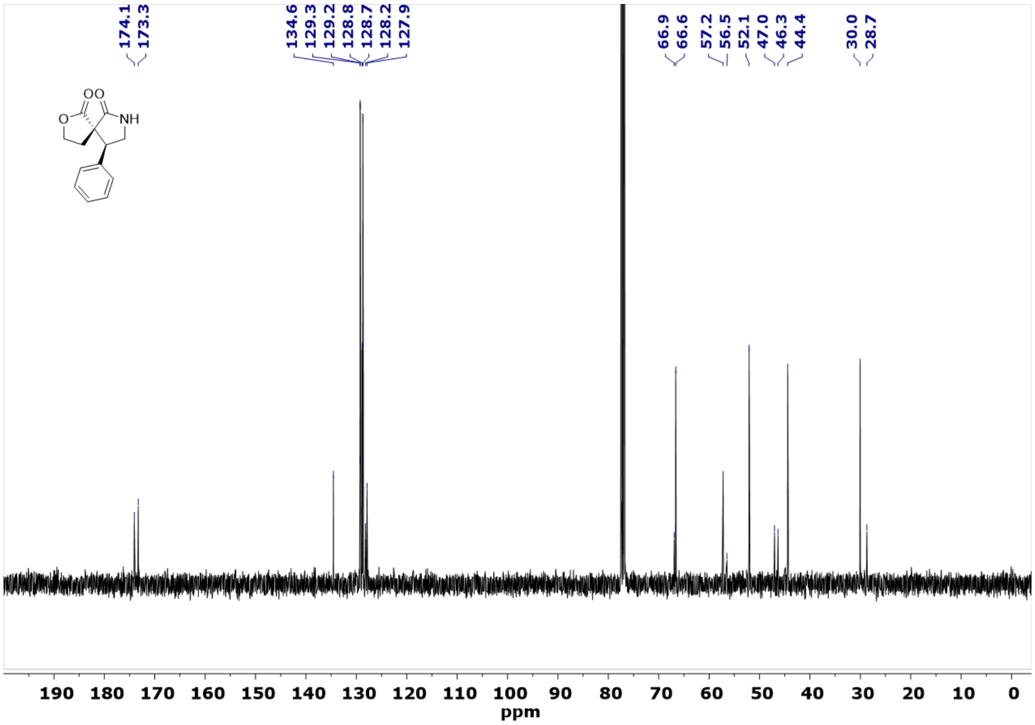

Figure S69: ^{13}C NMR, $CDCl_3$, 100 MHz (ppm) **15**

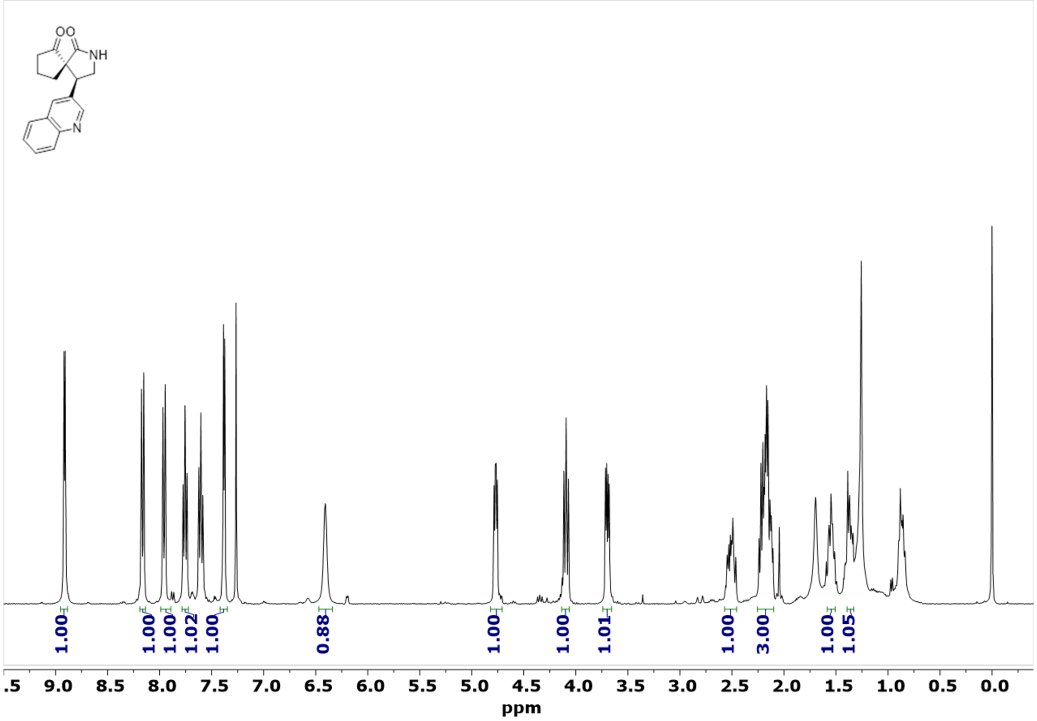

Figure S70: ^{1}H NMR, $CDCl_3$, 400 MHz (ppm) **10l**

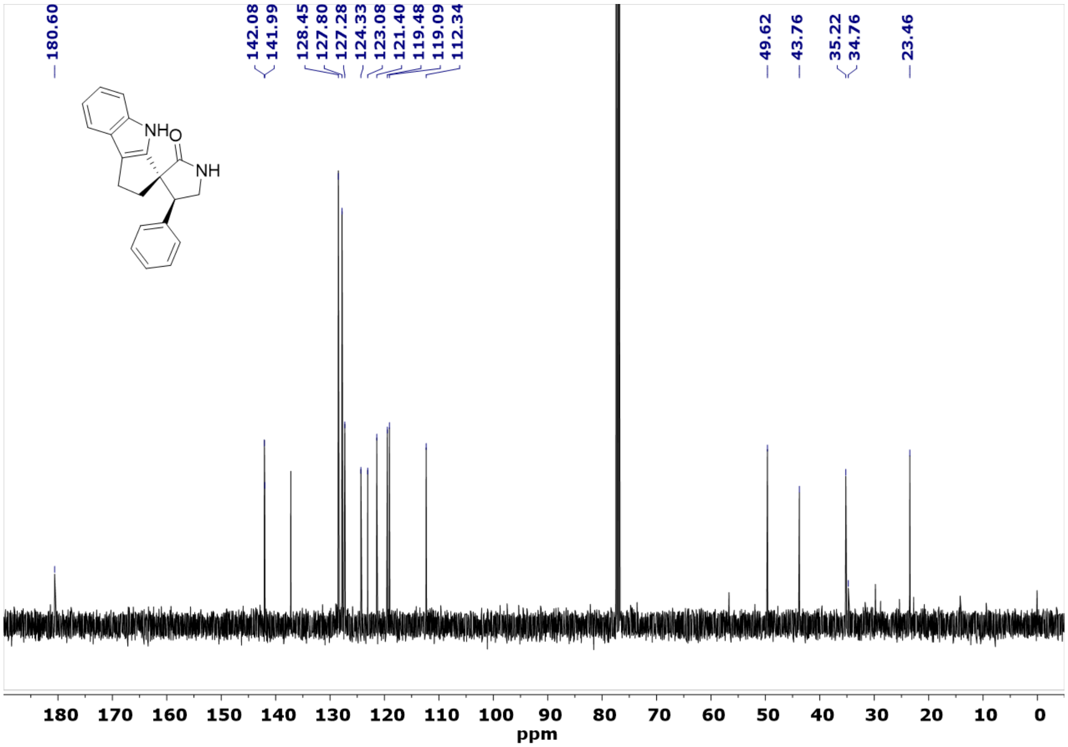

Figure S71: ^{13}C NMR, $CDCl_3$, 100 MHz (ppm) **10l**

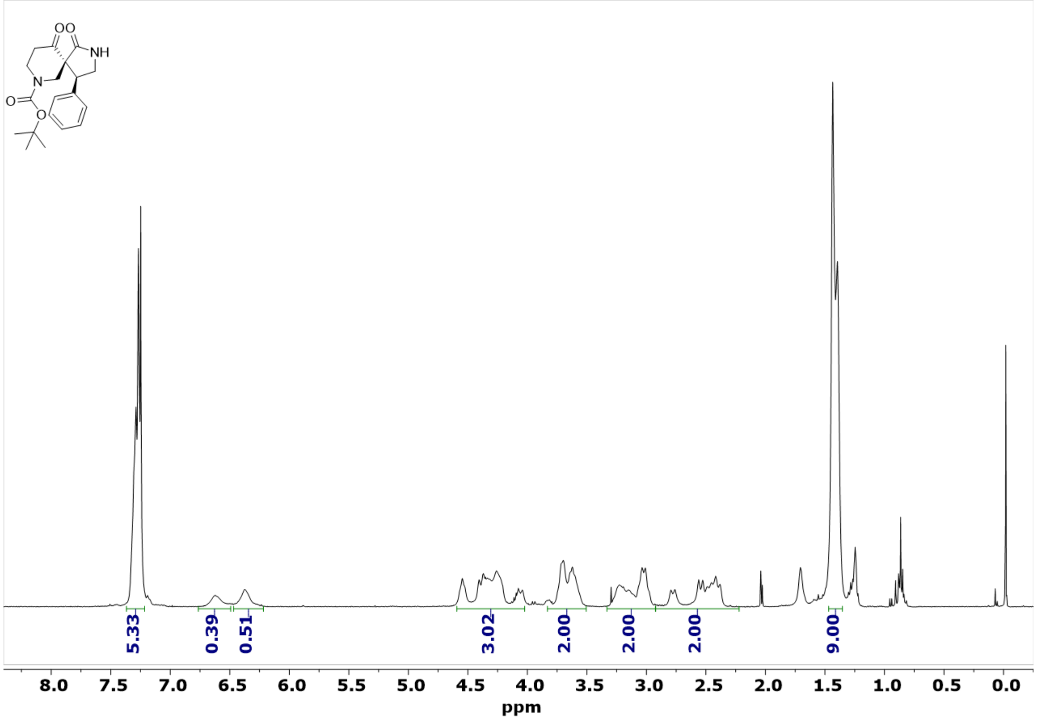

Figure S72: ^{1}H NMR, $CDCl_3$, 400 MHz (ppm) **16**

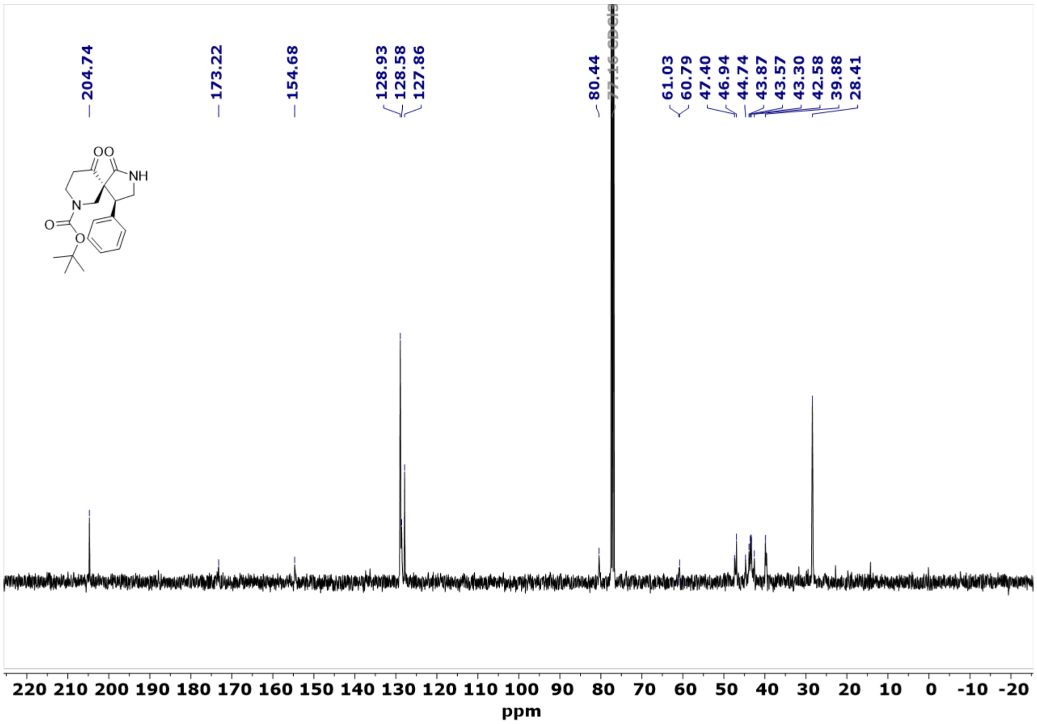

Figure S73: ^{13}C NMR, $CDCl_3$, 100 MHz (ppm) **16**

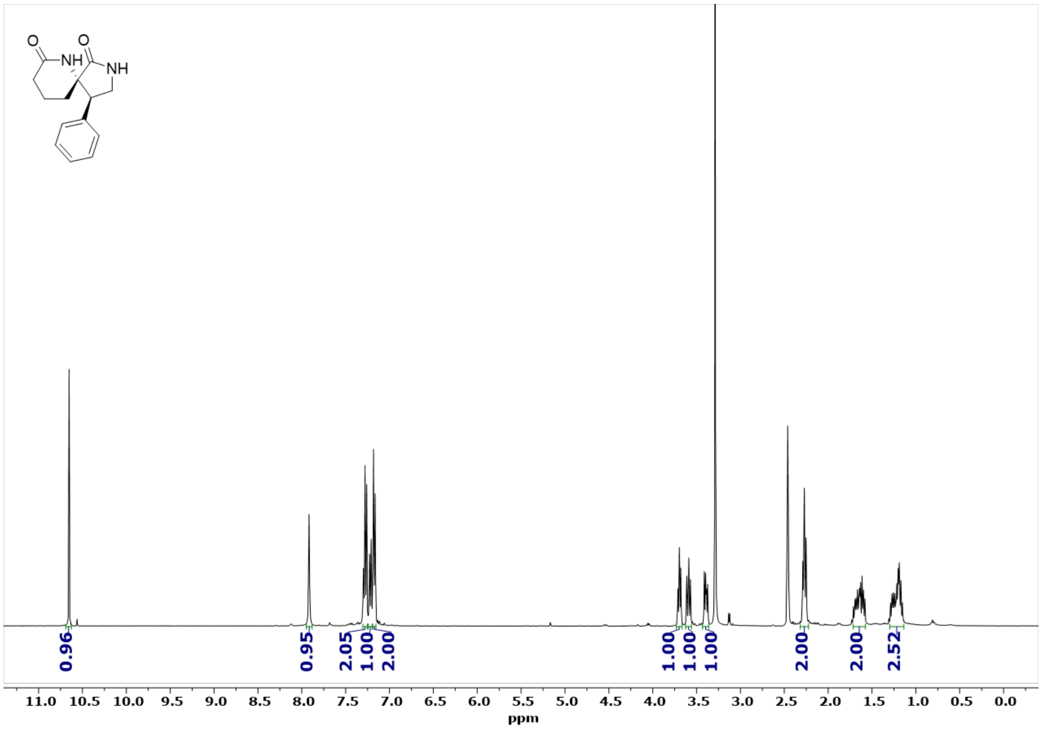

Figure S74: ^{1}H NMR, $CDCl_3$, 400 MHz (ppm) **17**

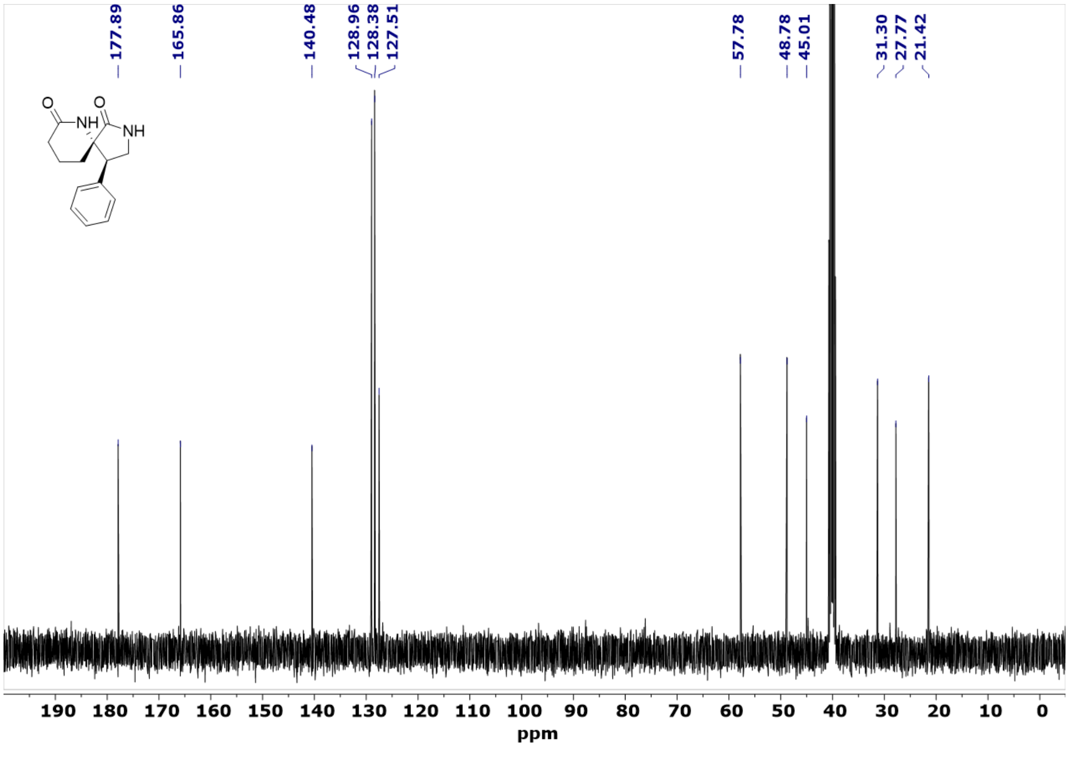

Figure S75: ^{13}C NMR, $CDCl_3$, 100 MHz (ppm) **17**

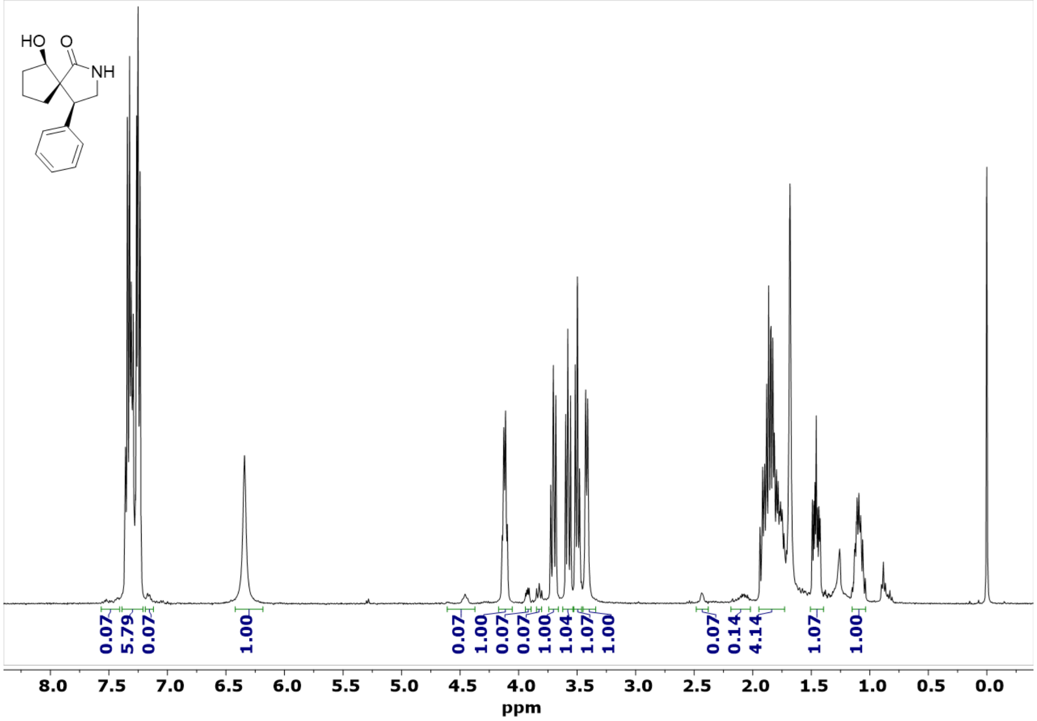

Figure S76: ^{1}H NMR, $CDCl_3$, 400 MHz (ppm) **18**

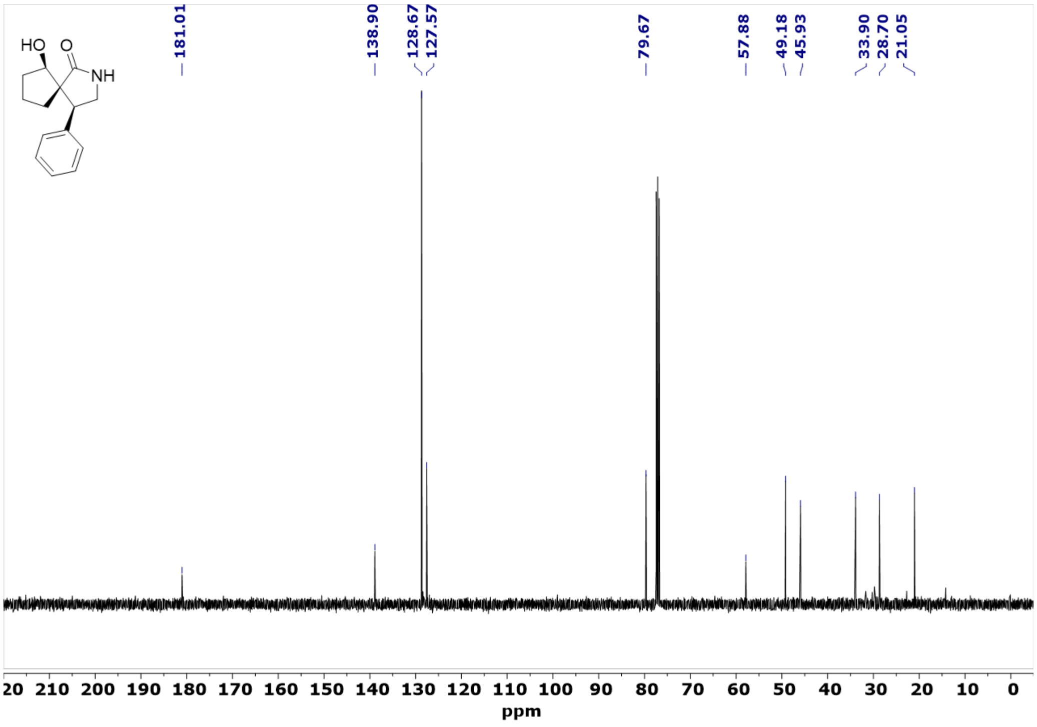

Figure S77: ^{13}C NMR, $CDCl_3$, 100 MHz (ppm) **18**

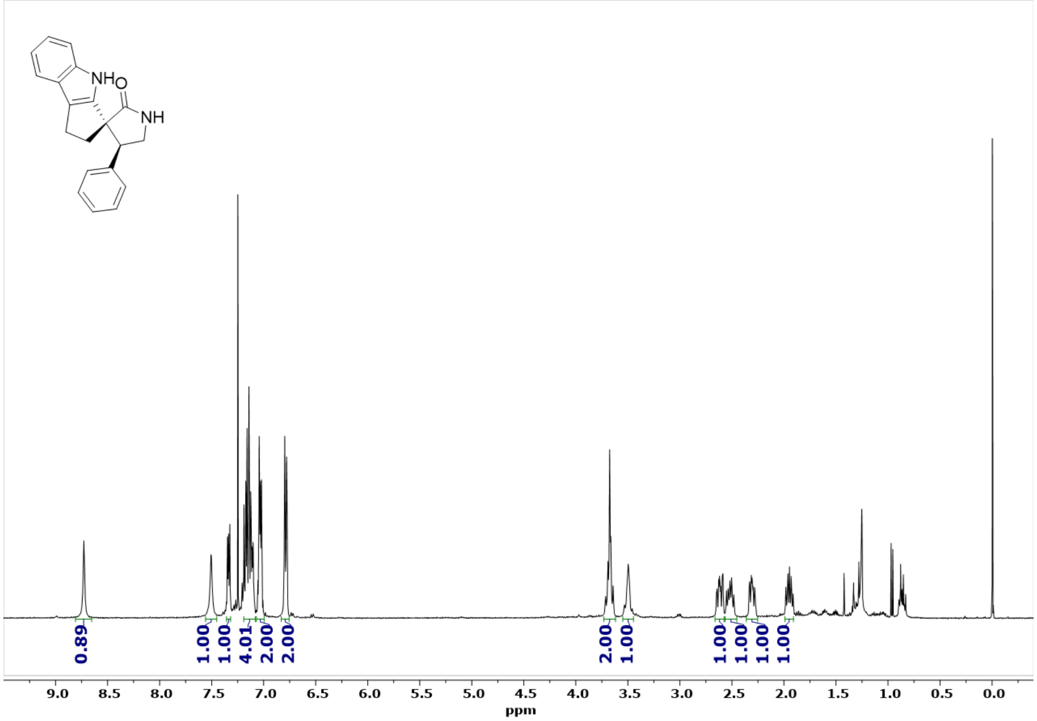

Figure S78: ^{1}H NMR, $CDCl_3$, 400 MHz (ppm) **19**

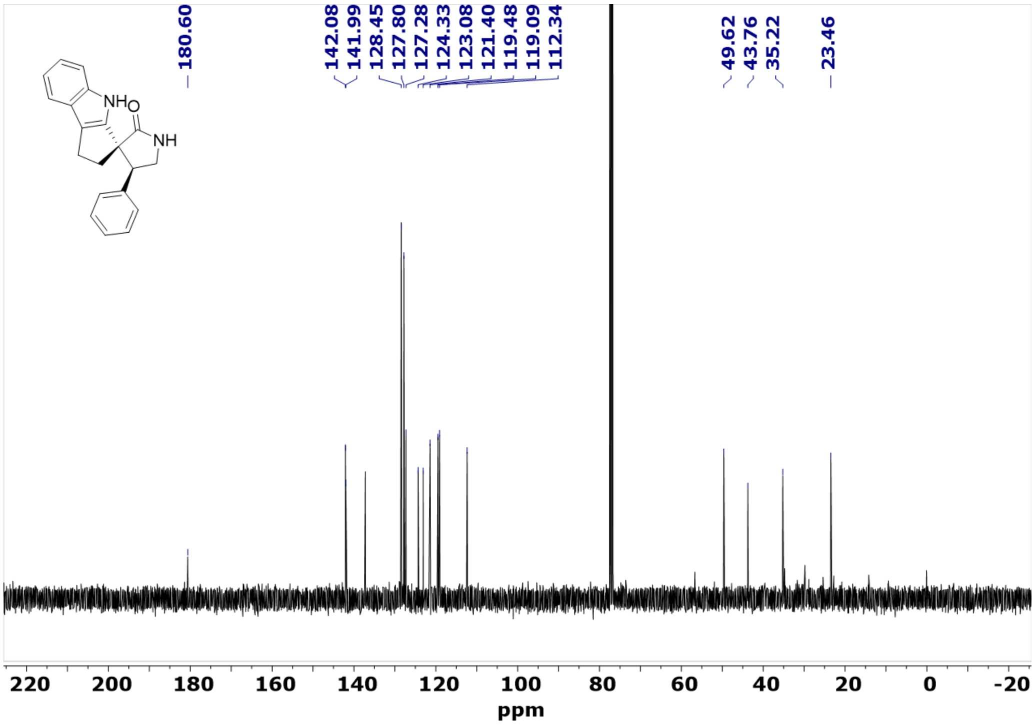

Figure S79: ^{13}C NMR, $CDCl_3$, 100 MHz (ppm) **19**

5. Chiral stationary phase HPLC chromatograms

3a: 98% ee, 99:1 d.r. HPLC Chiralpak IC-3, hexane/ethanol 85:15, 0.6 mL/min., 220 nm.

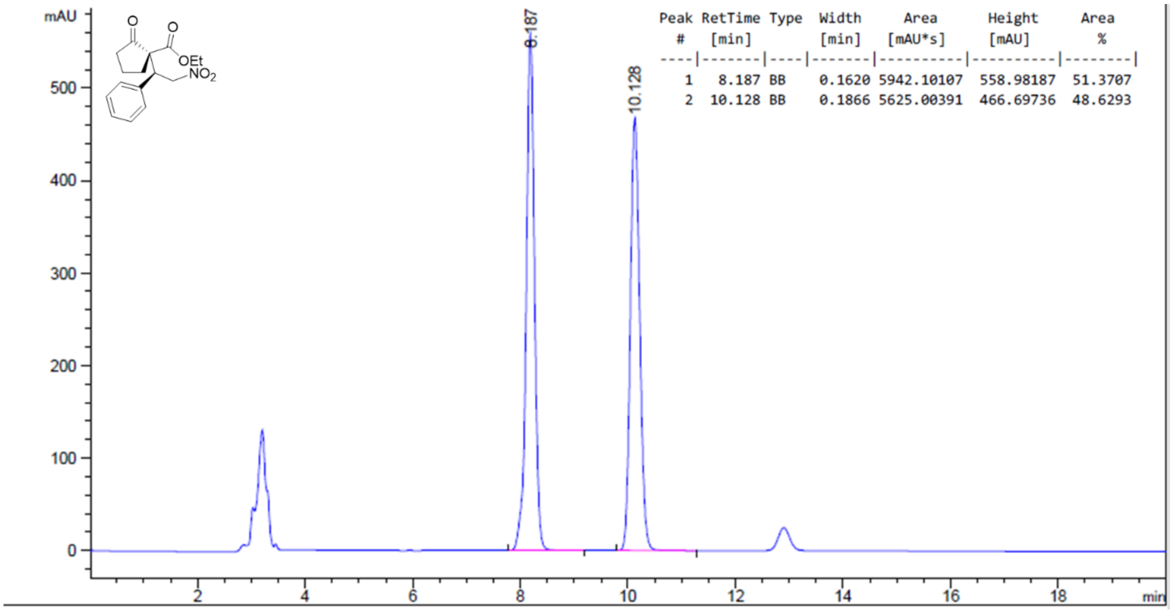

Peak #	RetTime [min]	Type	Width [min]	Area [mAU*s]	Height [mAU]	Area %
1	8.187	BB	0.1620	5942.10107	558.98187	51.3707
2	10.128	BB	0.1866	5625.00391	466.69736	48.6293

Figure **S80**: Racemic **3a**

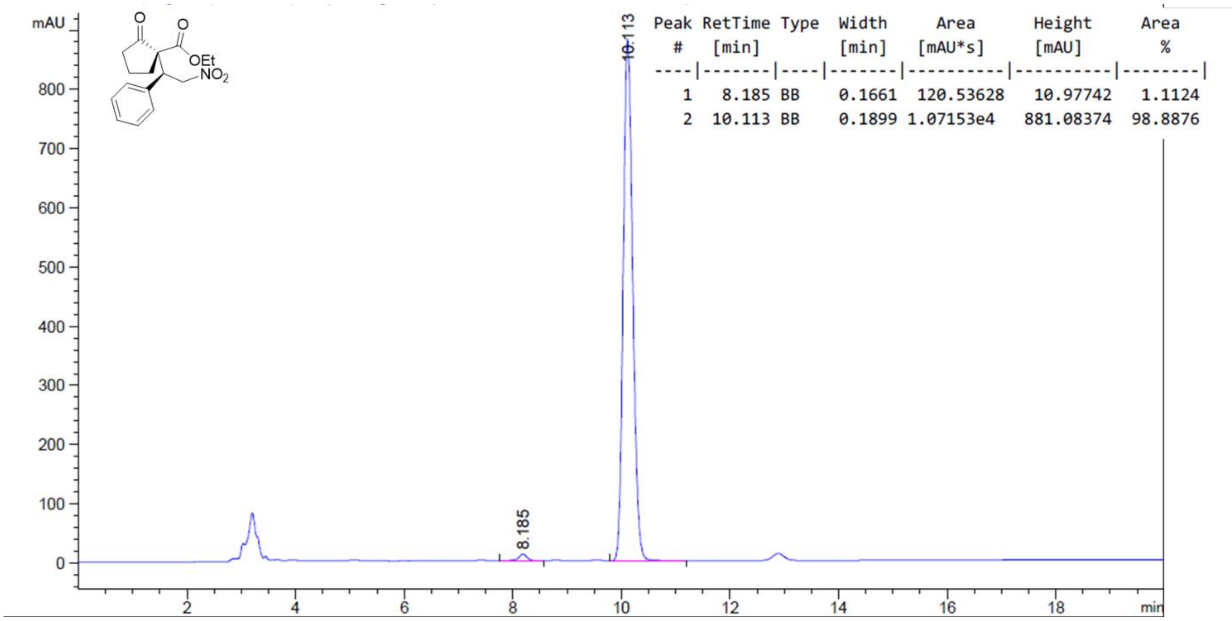

Peak #	RetTime [min]	Type	Width [min]	Area [mAU*s]	Height [mAU]	Area %
1	8.185	BB	0.1661	120.53628	10.97742	1.1124
2	10.113	BB	0.1899	1.07153e4	881.08374	98.8876

Figure S81: Enantiomerically enriched **3a**

3b: 99% ee, 98:2 d.r. HPLC Chiralpak IC-3, hexane/ethanol 85:15, 0.6 mL/min., 220 nm.

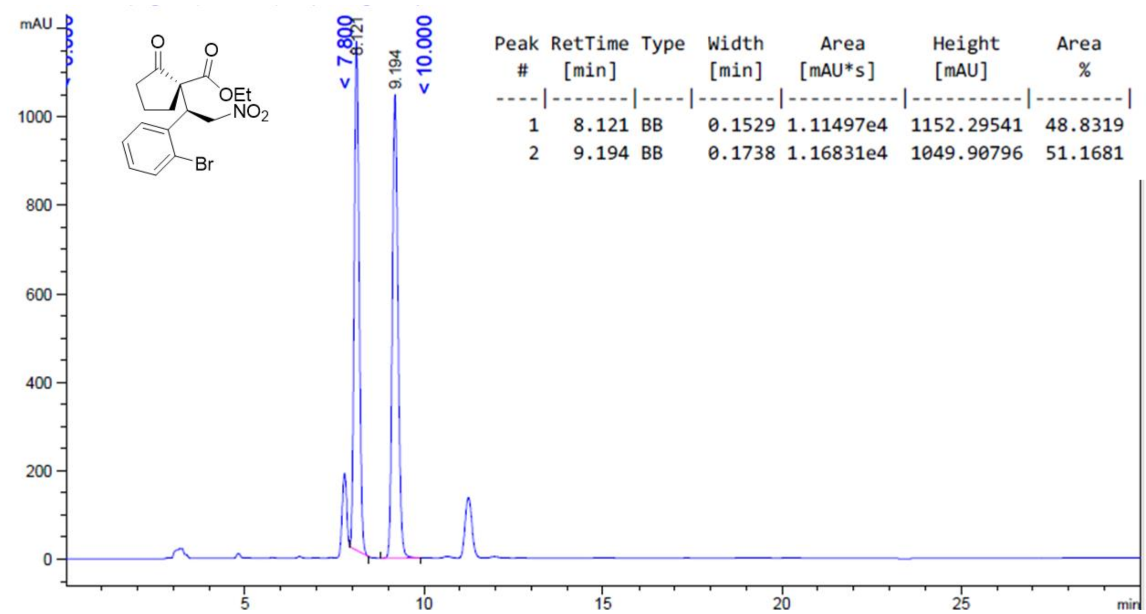

Peak #	RetTime [min]	Type	Width [min]	Area [mAU*s]	Height [mAU]	Area %
1	8.121	BB	0.1529	1.11497e4	1152.29541	48.8319
2	9.194	BB	0.1738	1.16831e4	1049.90796	51.1681

Figure S82: Racemic **3b**

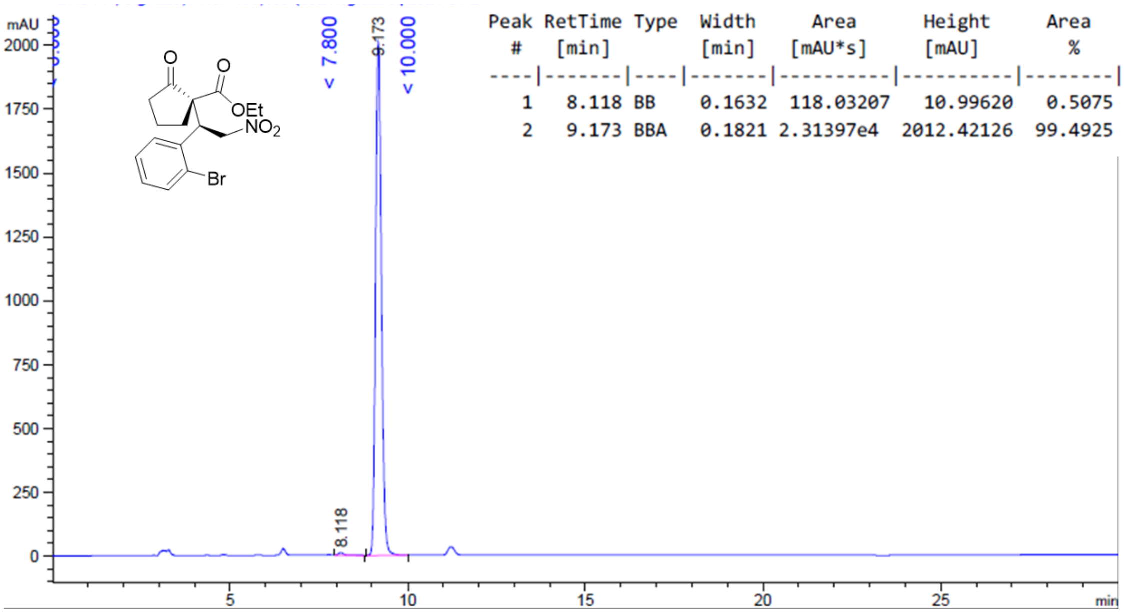

Peak #	RetTime [min]	Type	Width [min]	Area [mAU*s]	Height [mAU]	Area %
1	8.118	BB	0.1632	118.03207	10.99620	0.5075
2	9.173	BBA	0.1821	2.31397e4	2012.42126	99.4925

Figure S83: Enantiomerically enriched **3b**

3c: 97% ee, 99:1 d.r. HPLC Chiralpak IC-3, hexane/ethanol 85:15, 0.6 mL/min., 220 nm.

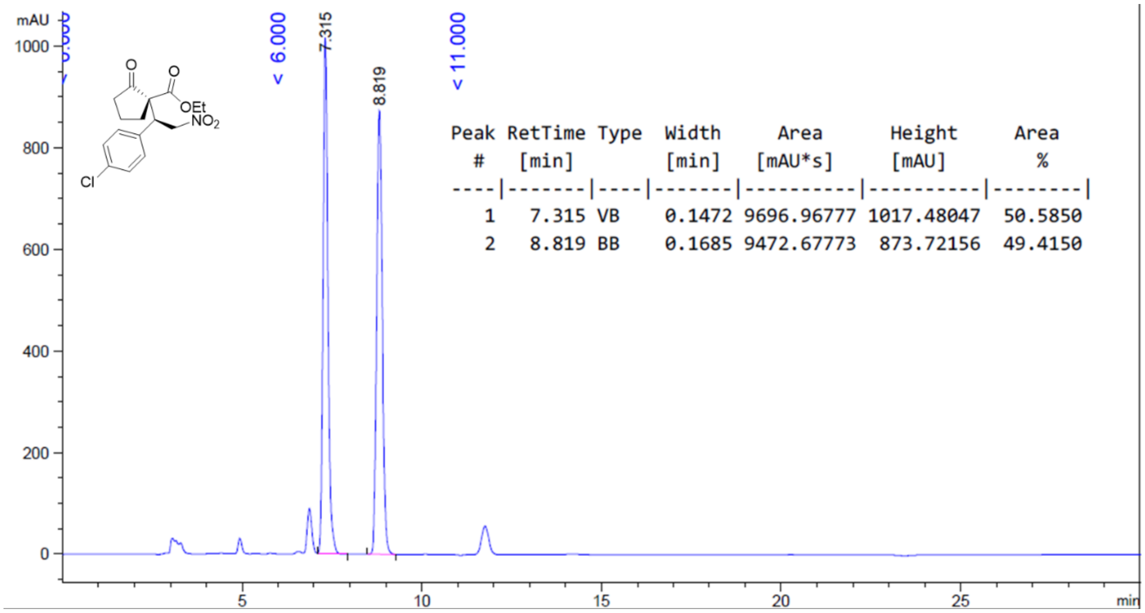

Peak #	RetTime [min]	Type	Width [min]	Area [mAU*s]	Height [mAU]	Area %
1	7.315	VB	0.1472	9696.96777	1017.48047	50.5850
2	8.819	BB	0.1685	9472.67773	873.72156	49.4150

Figure S84: Racemic **3c**

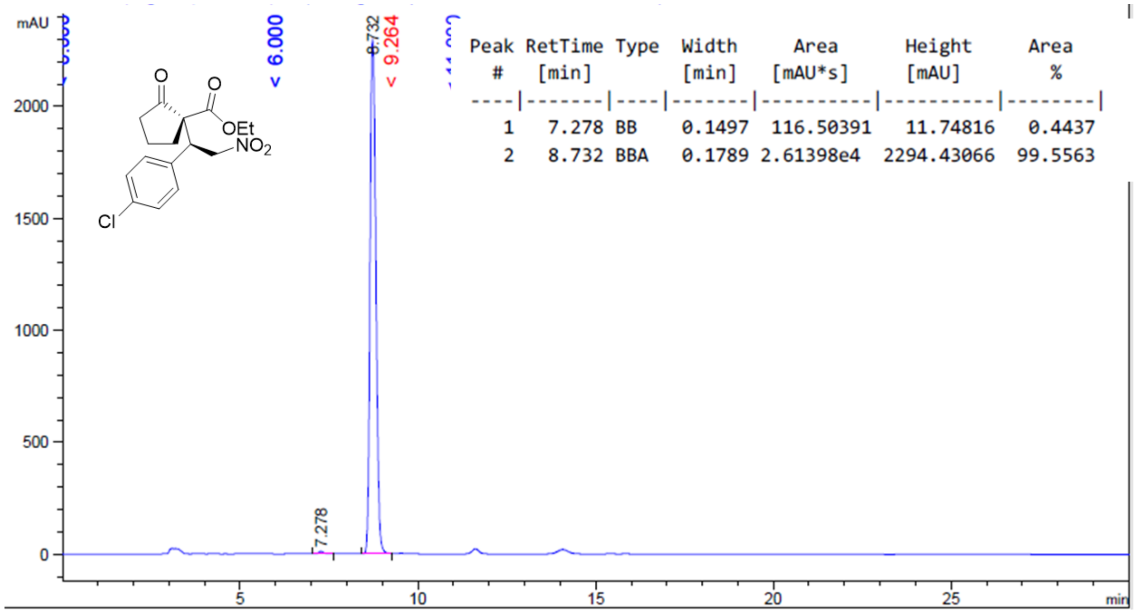

Peak #	RetTime [min]	Type	Width [min]	Area [mAU*s]	Height [mAU]	Area %
1	7.278	BB	0.1497	116.50391	11.74816	0.4437
2	8.732	BBA	0.1789	2.61398e4	2294.43066	99.5563

Figure S85: Enantiomerically enriched **3c**

3d: 98% ee, 98:2 d.r. HPLC Chiralpak IC-3, hexane/ethanol 85:15, 0.6 mL/min., 220 nm.

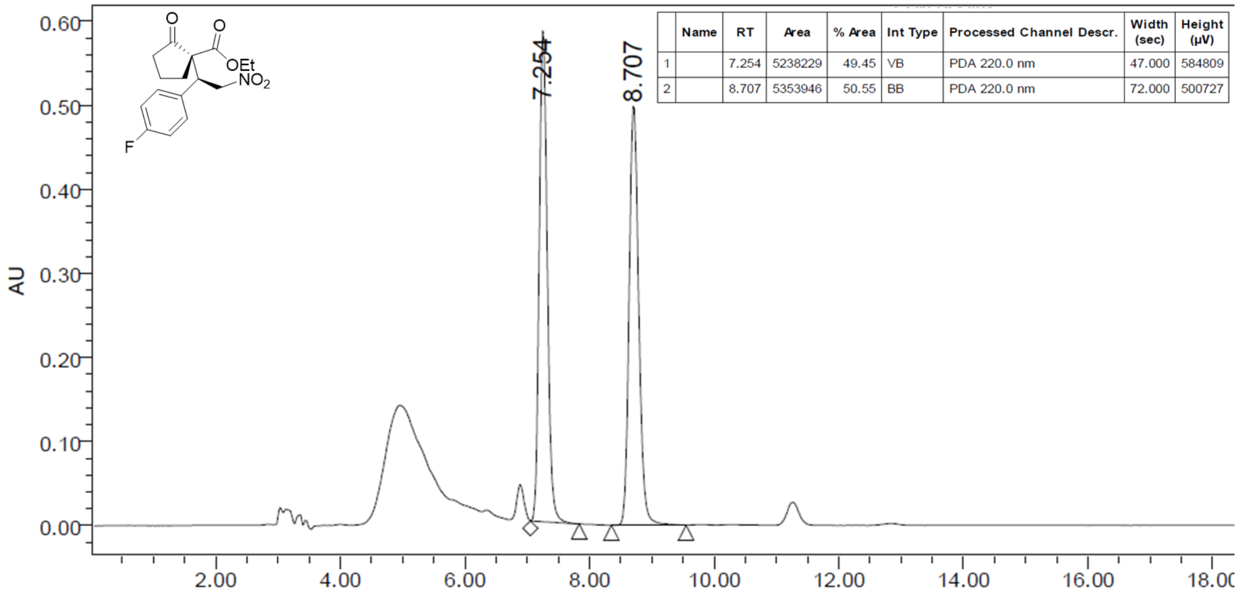

	Name	RT	Area	% Area	Int Type	Processed Channel Descr.	Width (sec)	Height (µV)
1		7.254	5238229	49.45	VB	PDA 220.0 nm	47.000	584809
2		8.707	5353946	50.55	BB	PDA 220.0 nm	72.000	500727

Figure S86: Racemic **3d**

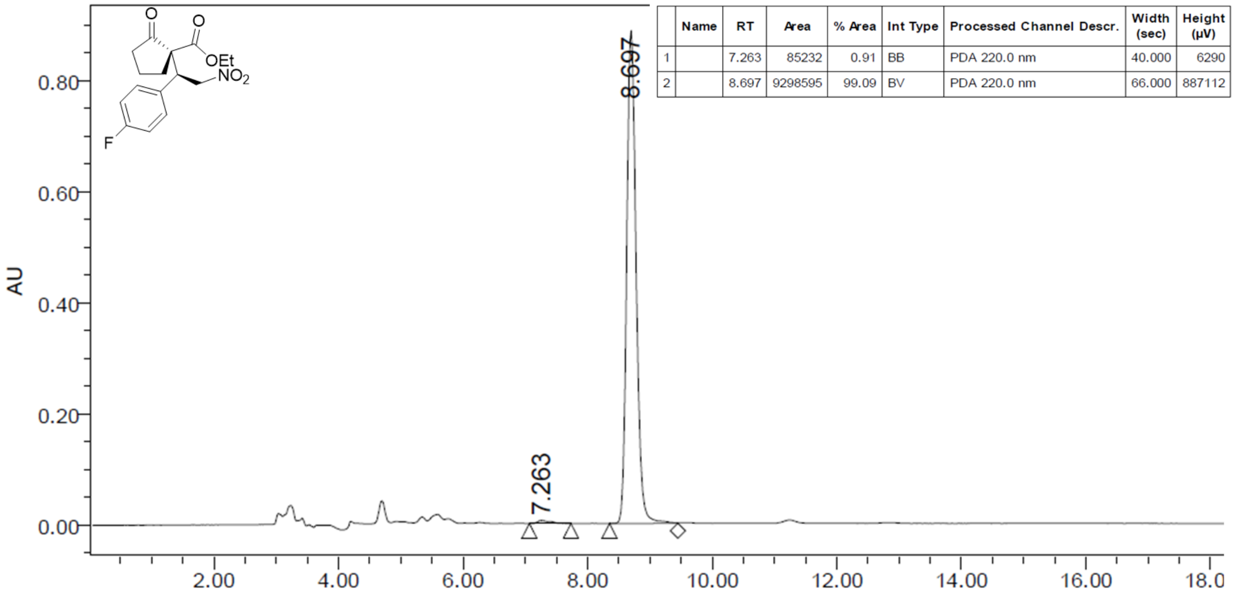

	Name	RT	Area	% Area	Int Type	Processed Channel Descr.	Width (sec)	Height (µV)
1		7.263	85232	0.91	BB	PDA 220.0 nm	40.000	6290
2		8.697	9298595	99.09	BV	PDA 220.0 nm	66.000	887112

Figure S87: Enantiomerically enriched **3d**

3e: Major diastereomer: 96% ee, minor diastereomer: >99% ee, 98:2 d.r. HPLC Chiralpak AD-H 250, hexane/isopropanol 90:10, 1.0 mL/min., 220 nm.

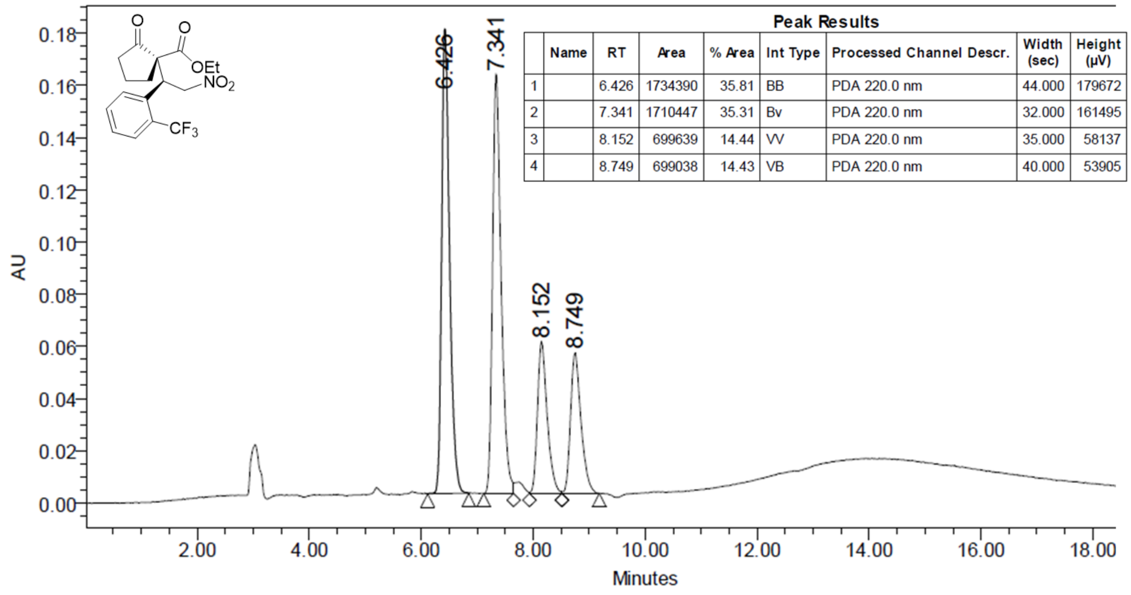

Figure S88: Racemic **3e**

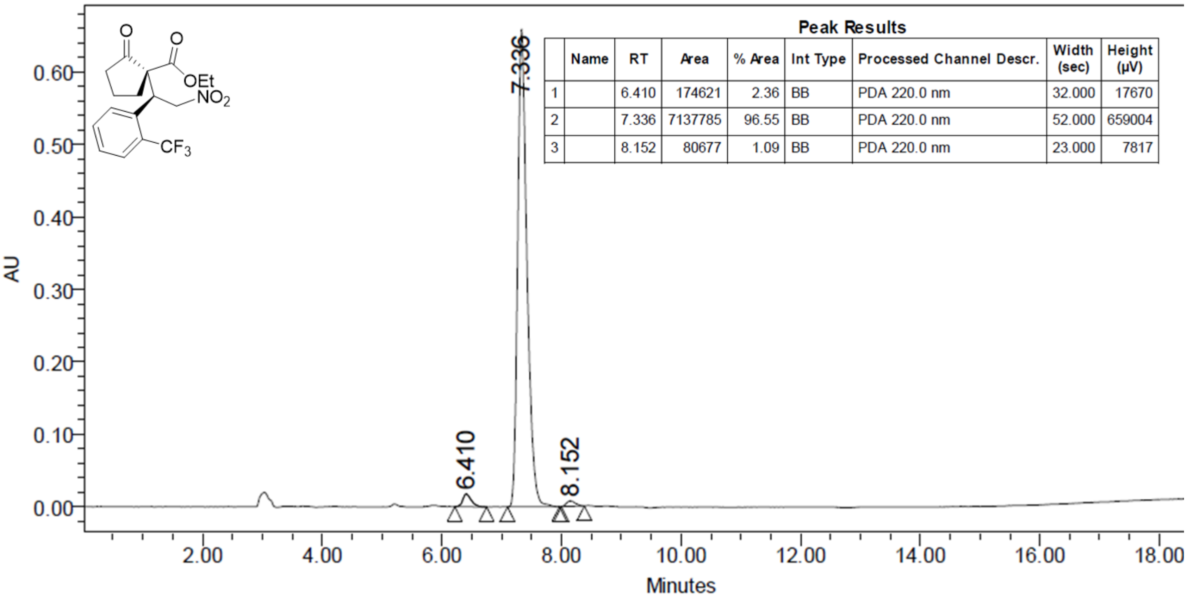

Figure S89: Enantiomerically enriched **3e**

3f: >99% ee, 99:1 d.r. HPLC Chiralpak AD-H 250, hexane/isopropanol 90:10, 0.5 mL/min., 220 nm.

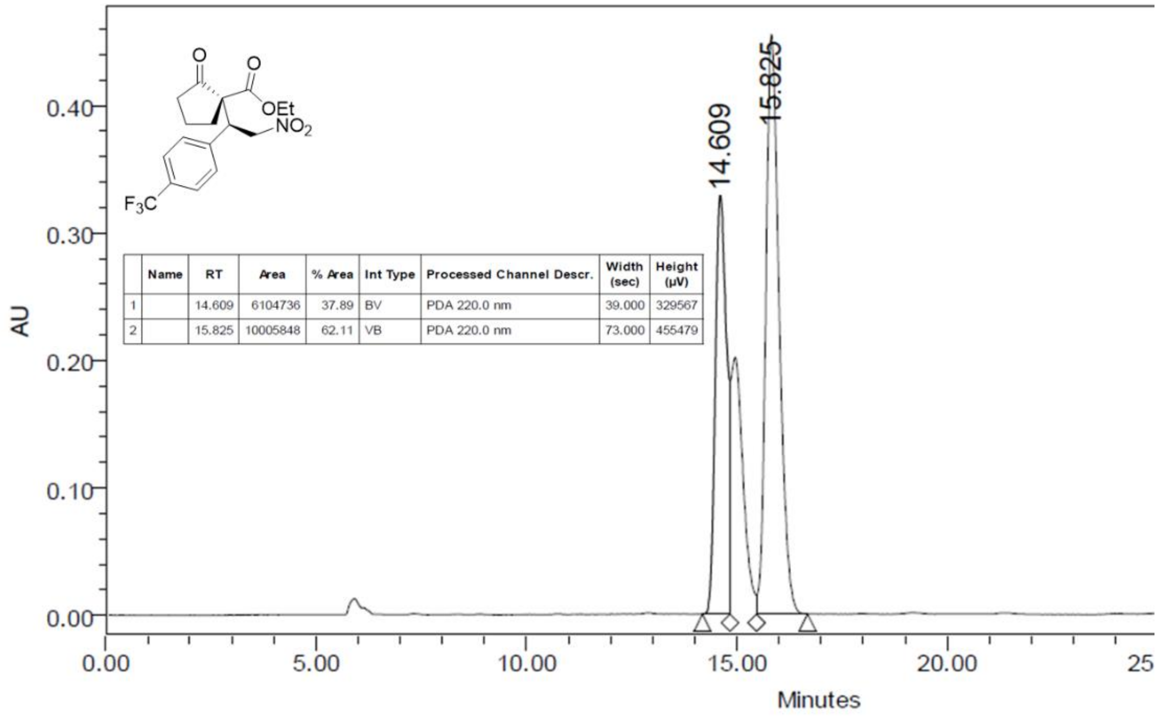

	Name	RT	Area	% Area	Int Type	Processed Channel Descr.	Width (sec)	Height (μV)
1		14.609	6104736	37.89	BV	PDA 220.0 nm	39.000	329567
2		15.825	10005848	62.11	VB	PDA 220.0 nm	73.000	455479

Figure S90: Racemic **3f**

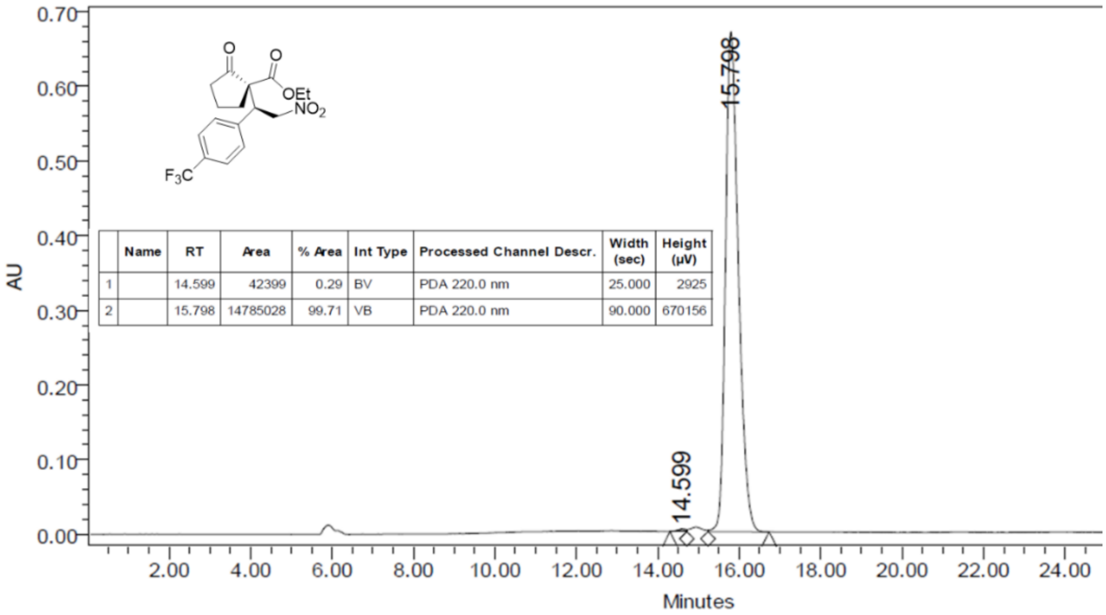

	Name	RT	Area	% Area	Int Type	Processed Channel Descr.	Width (sec)	Height (μV)
1		14.599	42399	0.29	BV	PDA 220.0 nm	25.000	2925
2		15.798	14785028	99.71	VB	PDA 220.0 nm	90.000	670156

Figure S91: Enantiomerically enriched **3f**

3g: 99% ee, >99:1 d.r. HPLC Chiralpak IC-3, hexane/ethanol 85:15, 0.6 mL/min., 220 nm.

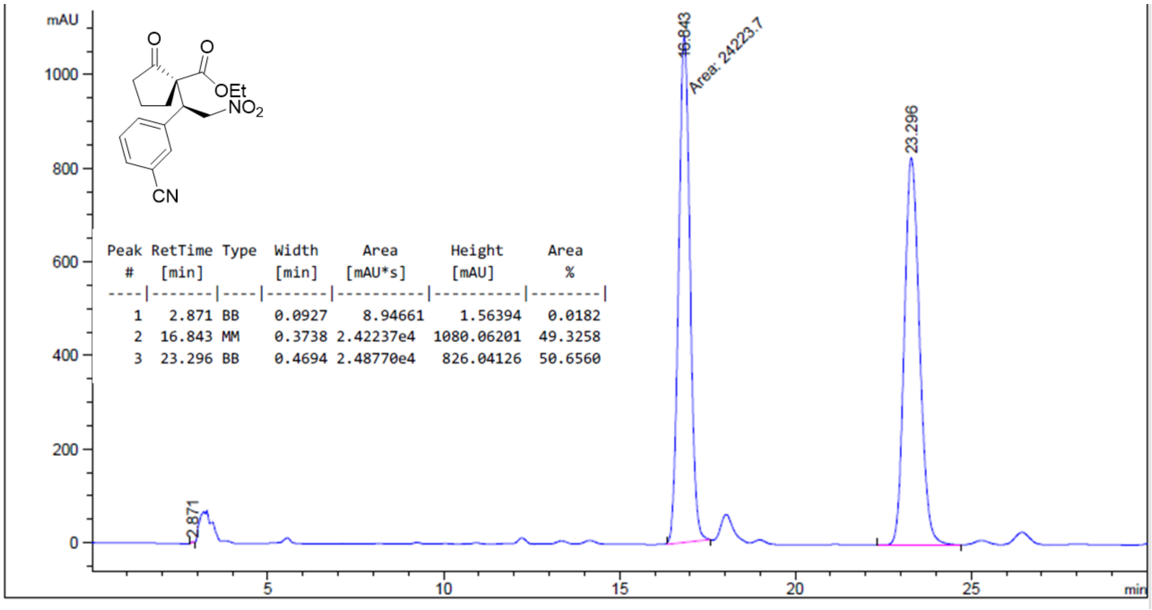

Figure S92: Racemic **3g**

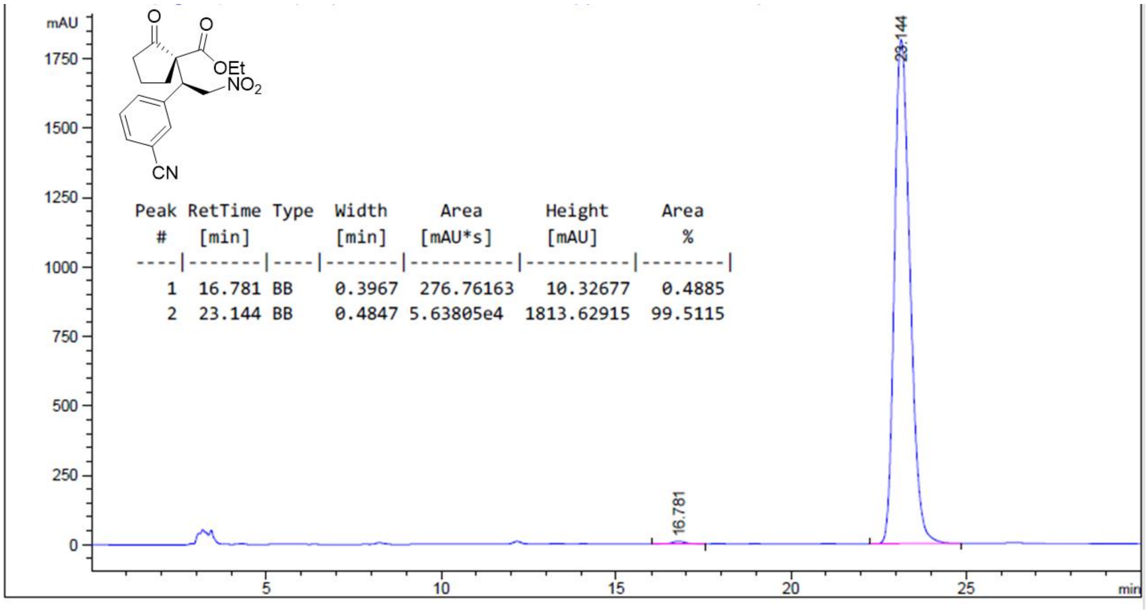

Figure S93: Enantiomerically enriched **3g**

3h: 98% ee, 98:2 d.r. HPLC Chiralpak IC-3, hexane/ethanol 85:15, 0.6 mL/min., 220 nm.

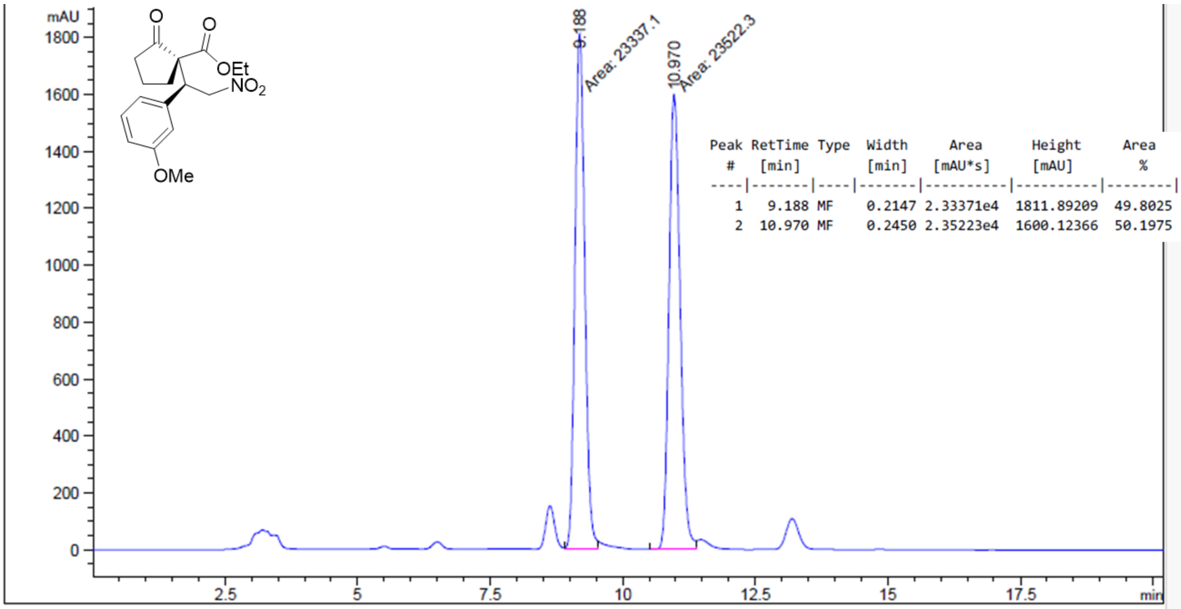

Figure S94: Racemic **3h**

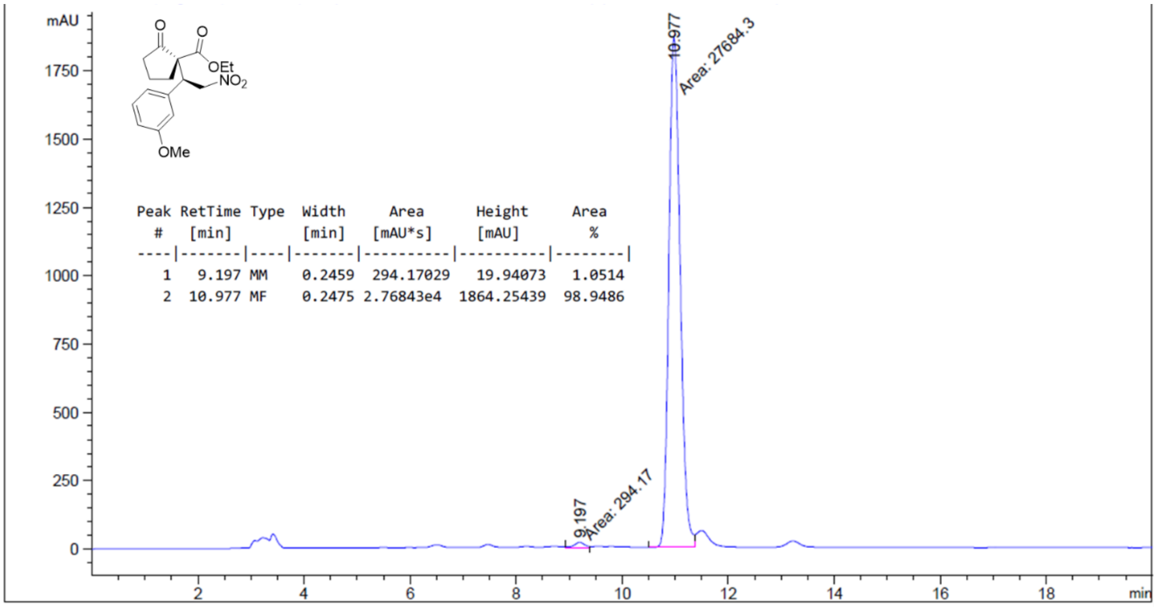

Figure S95: Enantiomerically enriched **3h**

3i: 98% ee, 95:5 d.r. HPLC Chiralpak IC-3, hexane/ethanol 85:15, 0.6 mL/min., 220 nm.

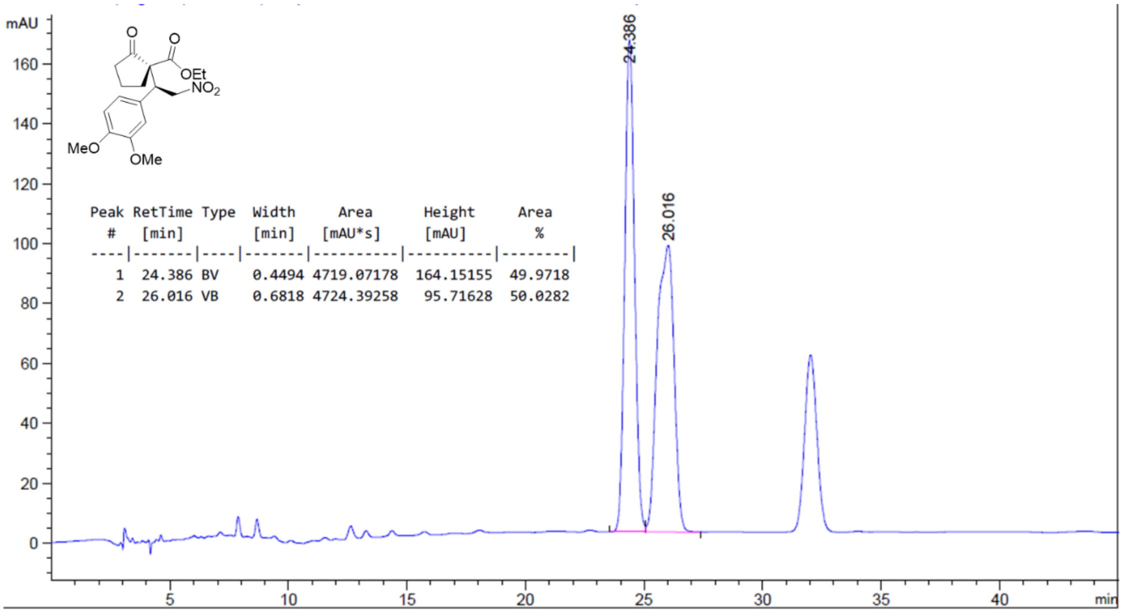

Figure S96: Racemic **3i**

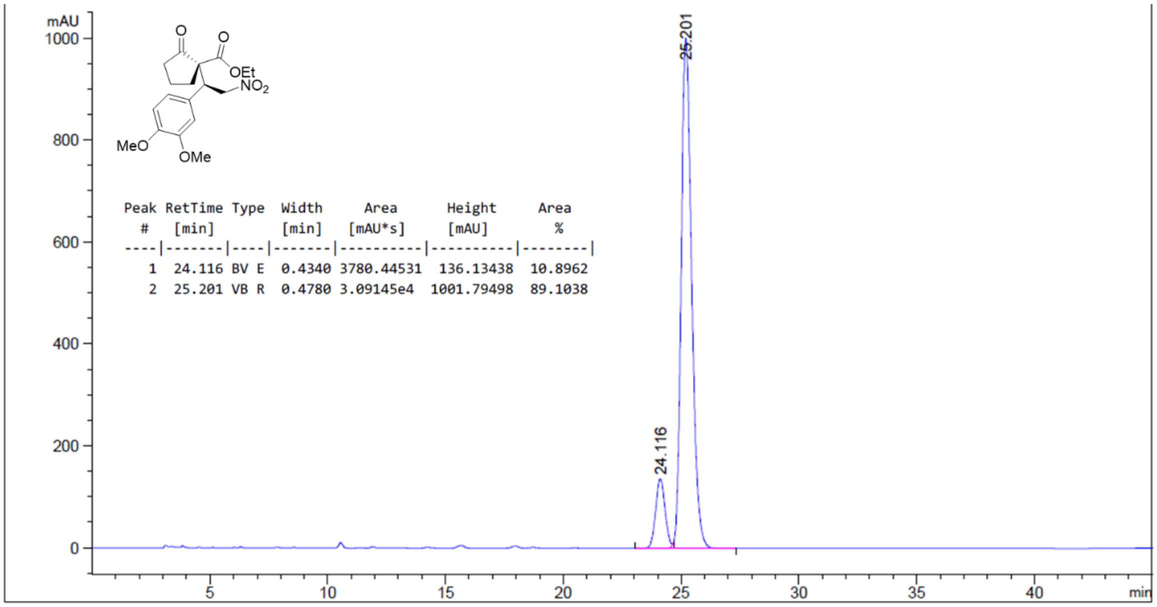

Figure S97: Enantiomerically enriched **3i**

3j: 77% ee, 98:2 d.r. HPLC Chiralpak IC-3, hexane/ethanol 85:15, 0.6 mL/min., 220 nm.

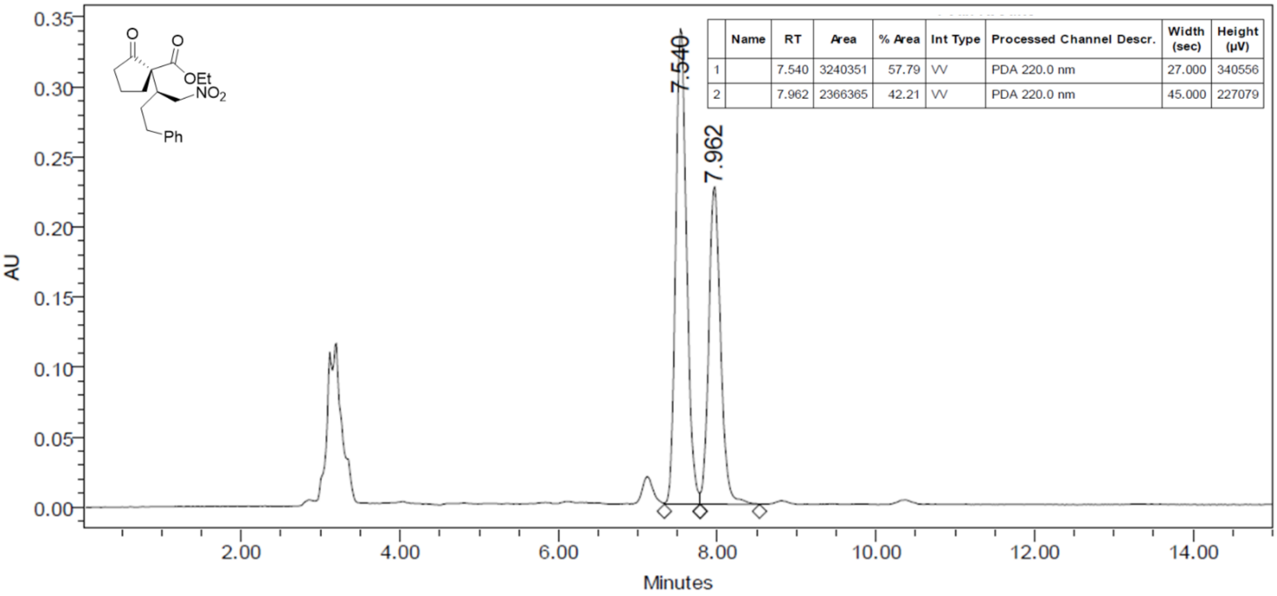

Figure S98: Racemic **3j**

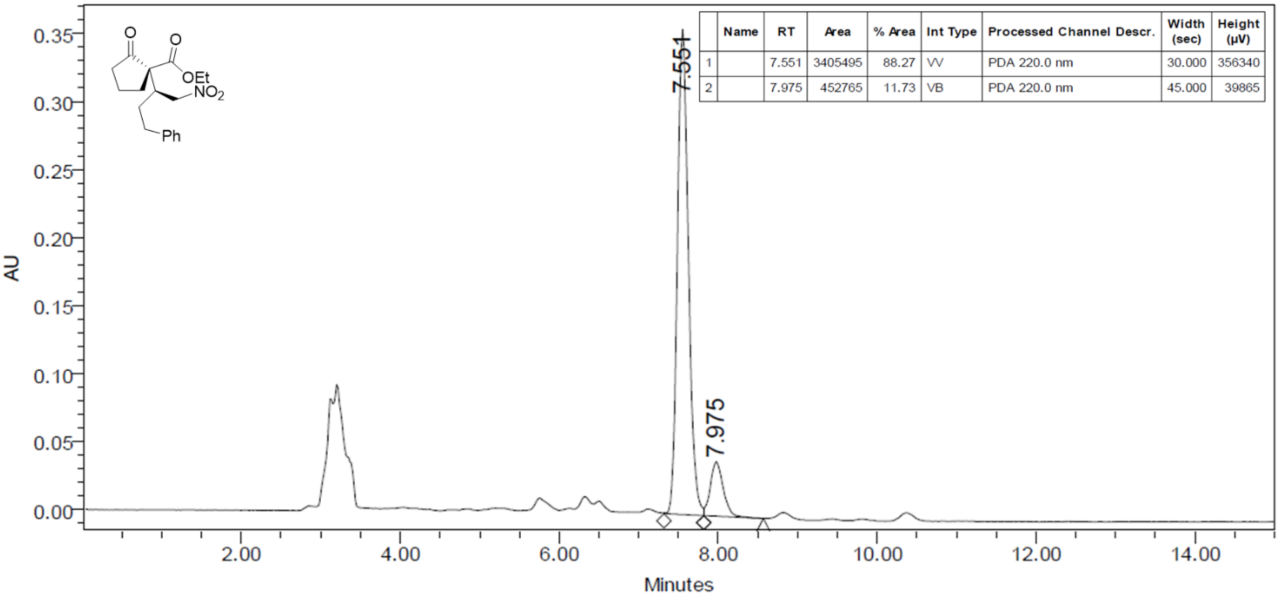

Figure S99: Enantiomerically enriched **3j**

3k: 97% ee, 98:2 d.r. HPLC Chiralpak IC-3, hexane/ethanol 85:15, 0.6 mL/min., 220 nm.

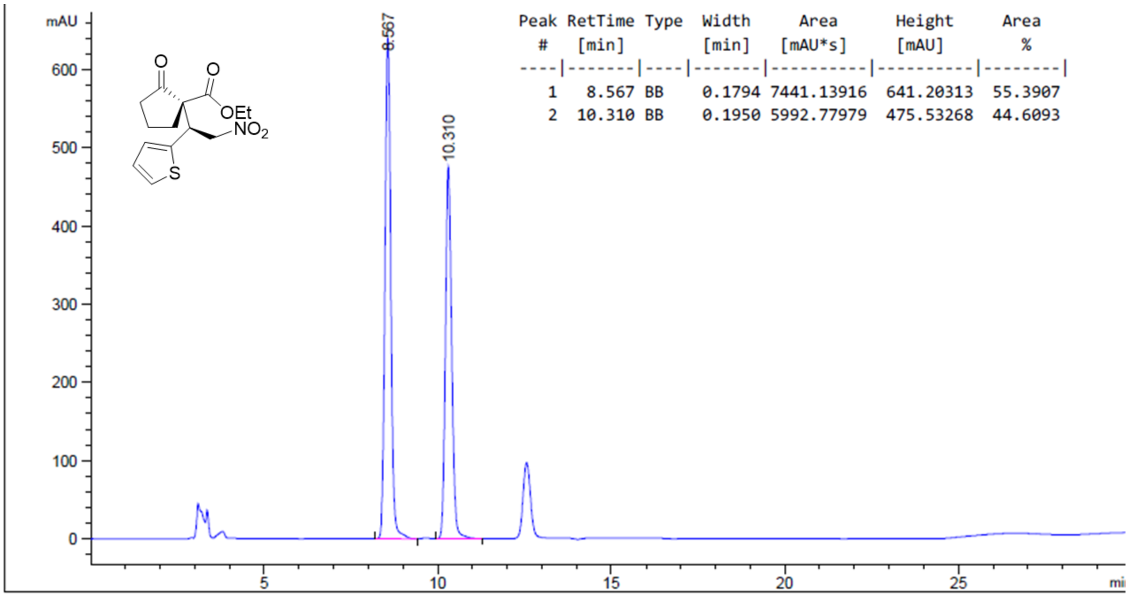

Figure S100: Racemic **3k**

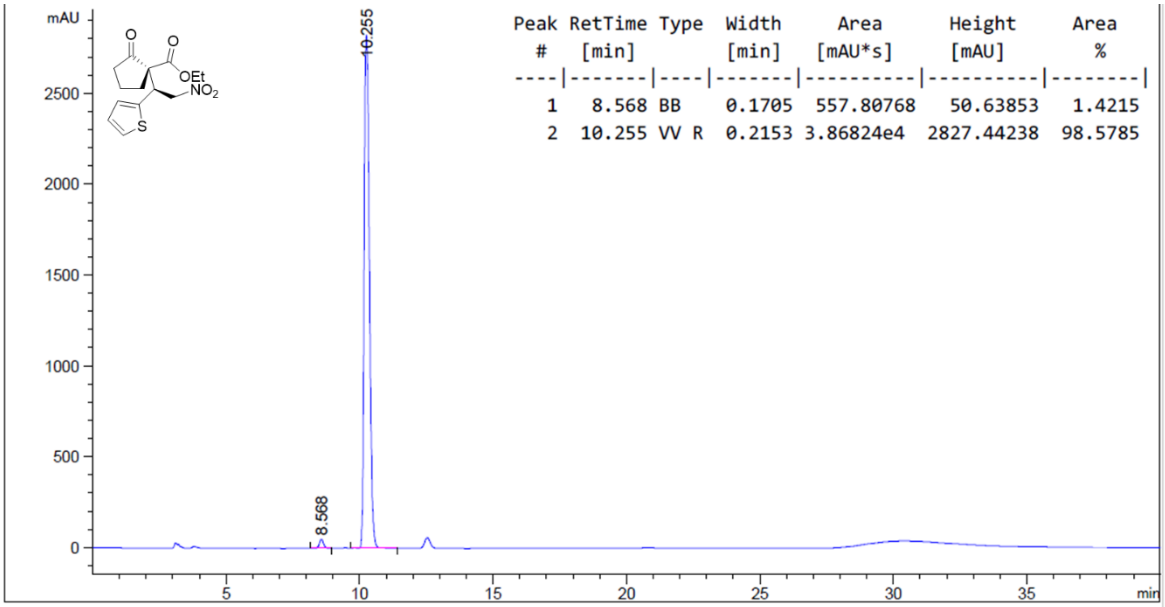

Figure S101: Enantiomerically enriched **3k**

3l: Major diastereomer: 98% ee, minor diastereomer: 90% ee, 97:3 d.r. HPLC Chiralpak IC-3, hexane/ethanol 85:15, 0.6 mL/min., 220 nm.

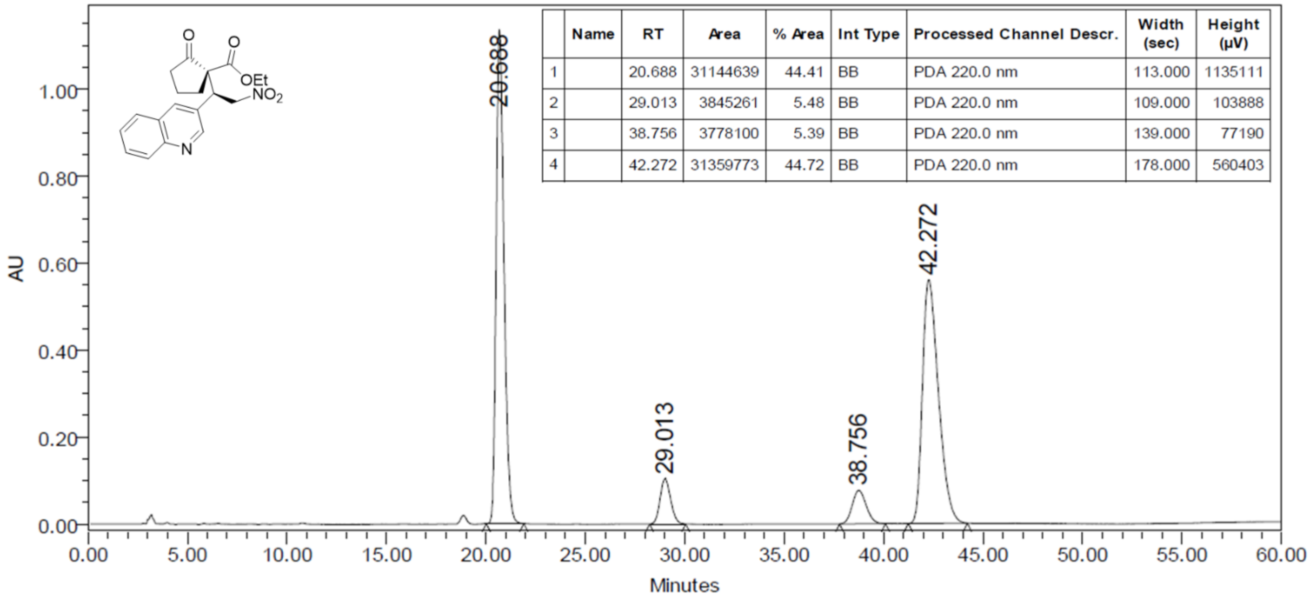

	Name	RT	Area	% Area	Int Type	Processed Channel Descr.	Width (sec)	Height (μV)
1		20.688	31144639	44.41	BB	PDA 220.0 nm	113.000	1135111
2		29.013	3845261	5.48	BB	PDA 220.0 nm	109.000	103888
3		38.756	3778100	5.39	BB	PDA 220.0 nm	139.000	77190
4		42.272	31359773	44.72	BB	PDA 220.0 nm	178.000	560403

Figure S102: Racemic **3l**

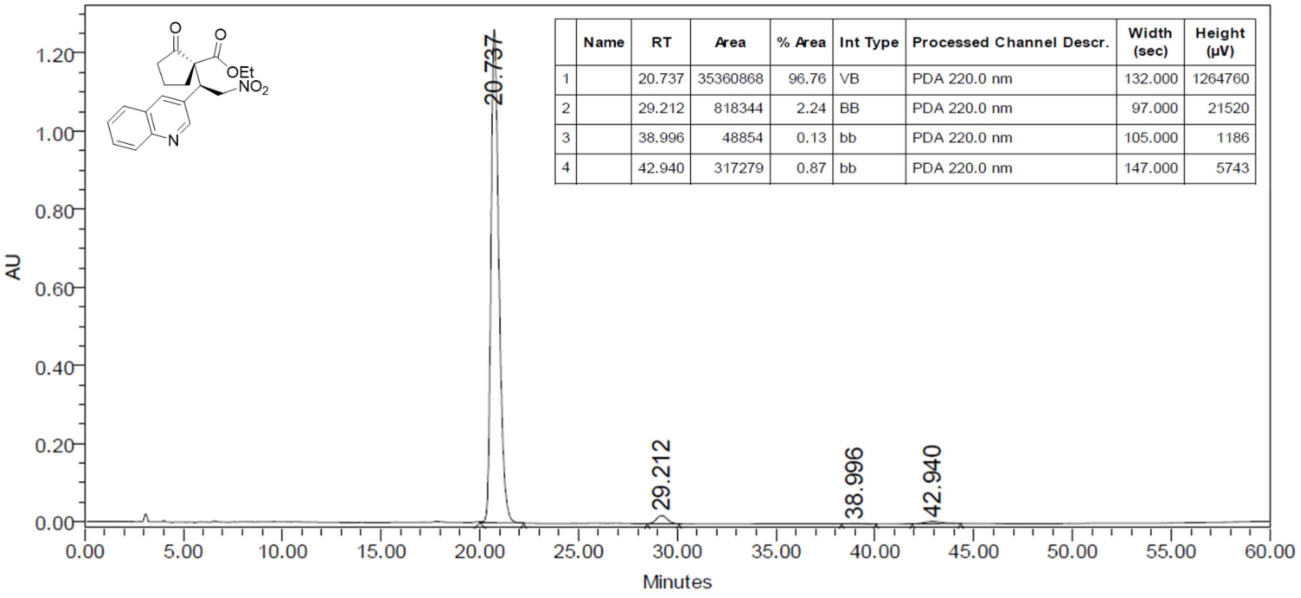

	Name	RT	Area	% Area	Int Type	Processed Channel Descr.	Width (sec)	Height (μV)
1		20.737	35360868	96.76	VB	PDA 220.0 nm	132.000	1264760
2		29.212	818344	2.24	BB	PDA 220.0 nm	97.000	21520
3		38.996	48854	0.13	bb	PDA 220.0 nm	105.000	1186
4		42.940	317279	0.87	bb	PDA 220.0 nm	147.000	5743

Figure S103: Enantiomerically enriched **3l**

4: Major diastereomer: 88% ee, minor diastereomer: 52% ee, 66:34 d.r. HPLC Chiralpak IC-3, hexane/ethanol 85:15, 0.6 mL/min., 220 nm.

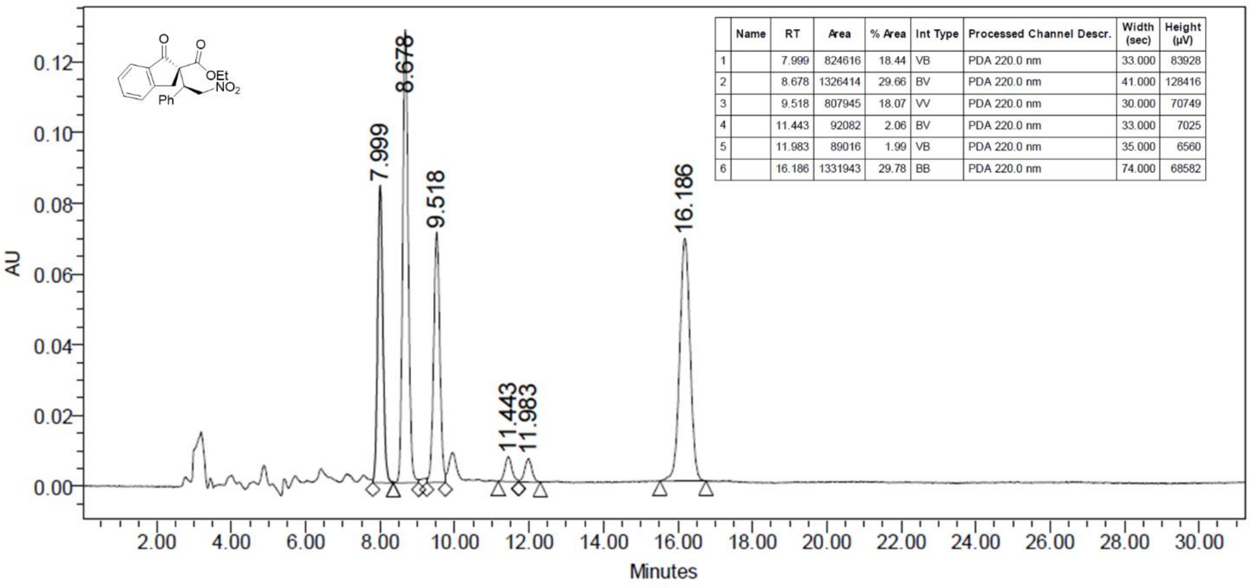

	Name	RT	Area	% Area	Int Type	Processed Channel Descr.	Width (sec)	Height (μV)
1		7.999	824616	18.44	VB	PDA 220.0 nm	33.000	83928
2		8.678	1326414	29.66	BV	PDA 220.0 nm	41.000	128416
3		9.518	807945	18.07	VV	PDA 220.0 nm	30.000	70749
4		11.443	92082	2.06	BV	PDA 220.0 nm	33.000	7025
5		11.983	89016	1.99	VB	PDA 220.0 nm	35.000	6560
6		16.186	1331943	29.78	BB	PDA 220.0 nm	74.000	68582

Figure S104: Racemic **4**

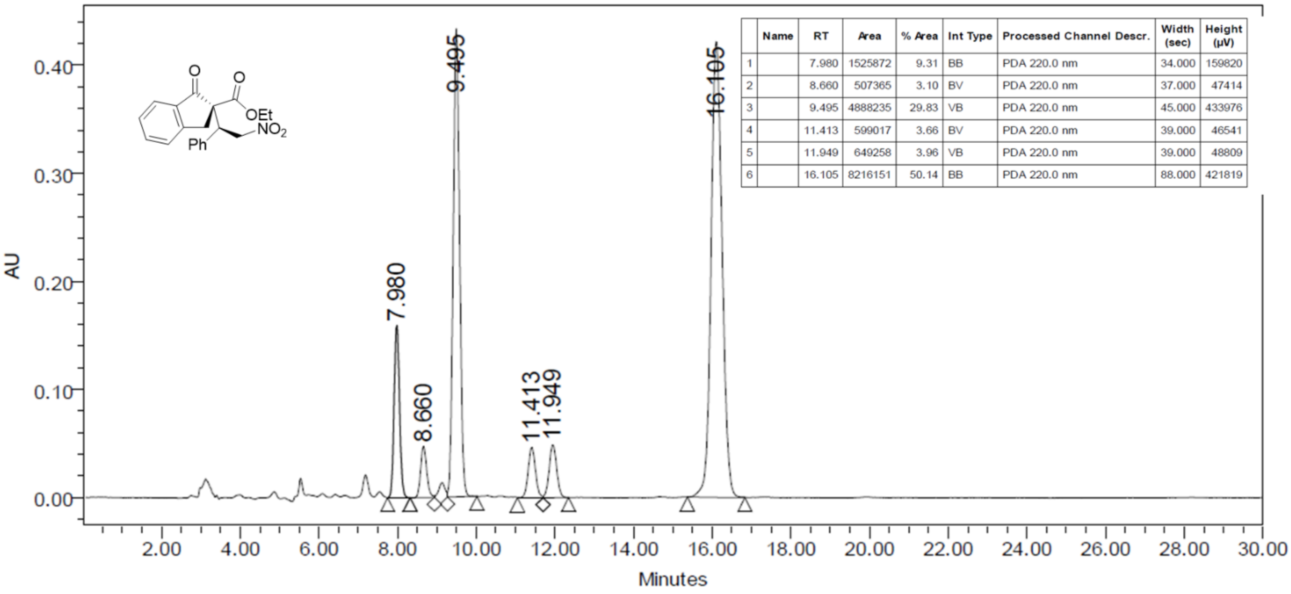

	Name	RT	Area	% Area	Int Type	Processed Channel Descr.	Width (sec)	Height (μV)
1		7.980	1525872	9.31	BB	PDA 220.0 nm	34.000	159820
2		8.660	507365	3.10	BV	PDA 220.0 nm	37.000	47414
3		9.495	4888235	29.83	VB	PDA 220.0 nm	45.000	433976
4		11.413	599017	3.66	BV	PDA 220.0 nm	39.000	46541
5		11.949	649258	3.96	VB	PDA 220.0 nm	39.000	48809
6		16.105	8216151	50.14	BB	PDA 220.0 nm	88.000	421819

Figure S105: Enantiomerically enriched **4**

5: Major diastereomer: 96% ee, minor diastereomer: >99% ee, 96:4 d.r. HPLC Chiralpak IC-3, hexane/ethanol 90:10, 1.0 mL/min., 220 nm.

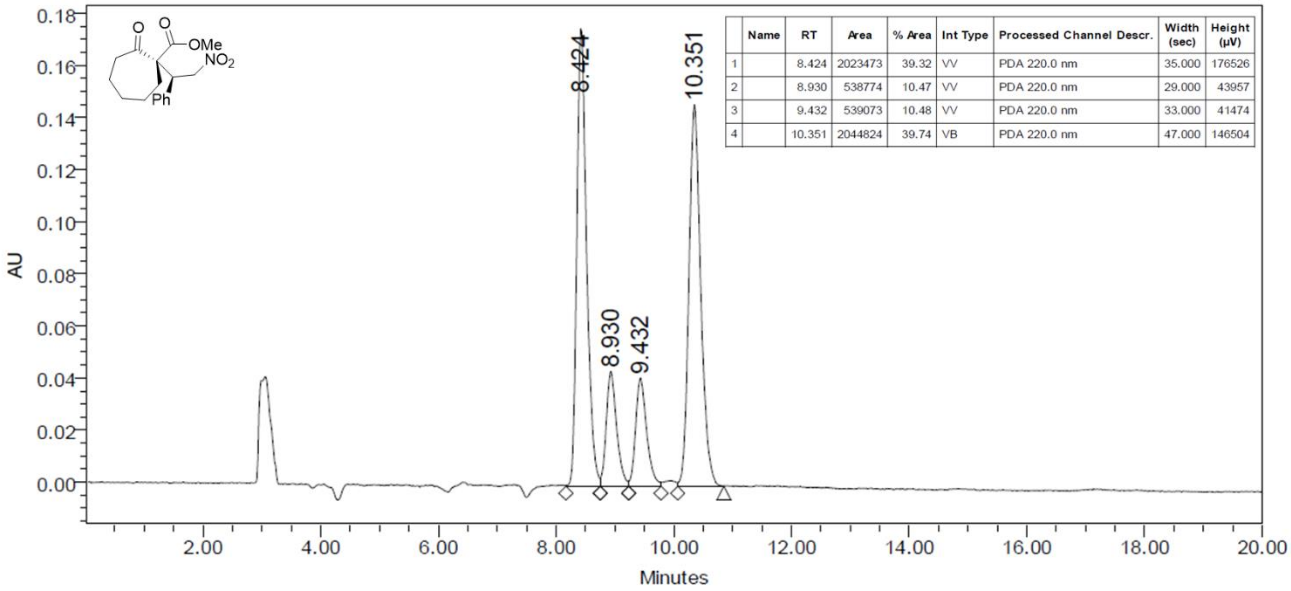

	Name	RT	Area	% Area	Int Type	Processed Channel Descr.	Width (sec)	Height (µV)
1		8.424	2023473	39.32	VV	PDA 220.0 nm	35.000	176526
2		8.930	538774	10.47	VV	PDA 220.0 nm	29.000	43957
3		9.432	539073	10.48	VV	PDA 220.0 nm	33.000	41474
4		10.351	2044824	39.74	VB	PDA 220.0 nm	47.000	146504

Figure S106: Racemic **5**

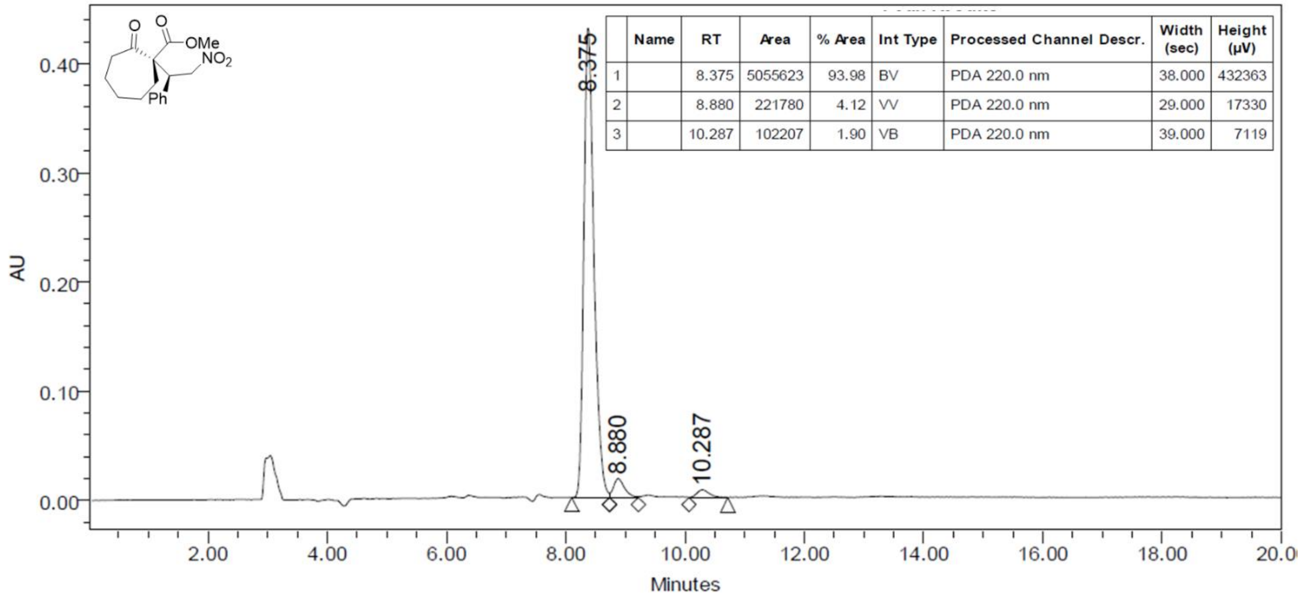

	Name	RT	Area	% Area	Int Type	Processed Channel Descr.	Width (sec)	Height (µV)
1		8.375	5055623	93.98	BV	PDA 220.0 nm	38.000	432363
2		8.880	221780	4.12	VV	PDA 220.0 nm	29.000	17330
3		10.287	102207	1.90	VB	PDA 220.0 nm	39.000	7119

Figure S107: Enantiomerically enriched **5**

6: Major diastereomer: 97% ee, minor diastereomer: >99% ee, 89:11 d.r. HPLC Chiralpak IA 250, hexane/ethanol 85:15, 1.0 mL/min., 220 nm.

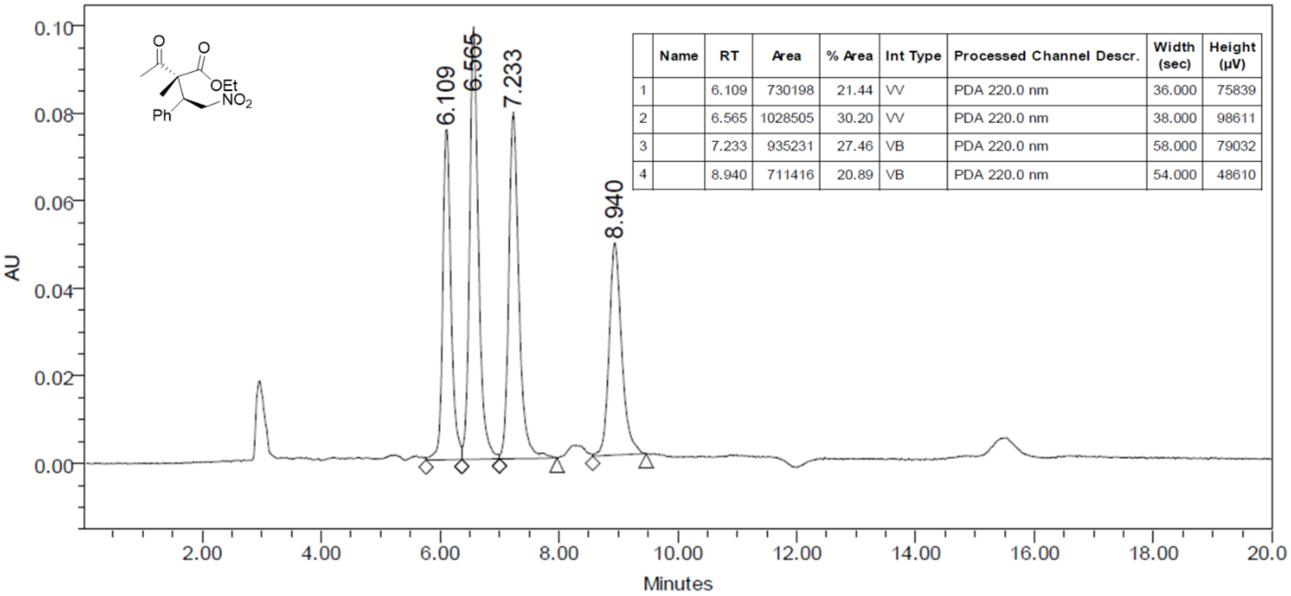

	Name	RT	Area	% Area	Int Type	Processed Channel Descr.	Width (sec)	Height (μV)
1		6.109	730198	21.44	VV	PDA 220.0 nm	36.000	75839
2		6.565	1028505	30.20	VV	PDA 220.0 nm	38.000	98611
3		7.233	935231	27.46	VB	PDA 220.0 nm	58.000	79032
4		8.940	711416	20.89	VB	PDA 220.0 nm	54.000	48610

Figure S108: Racemic **6**

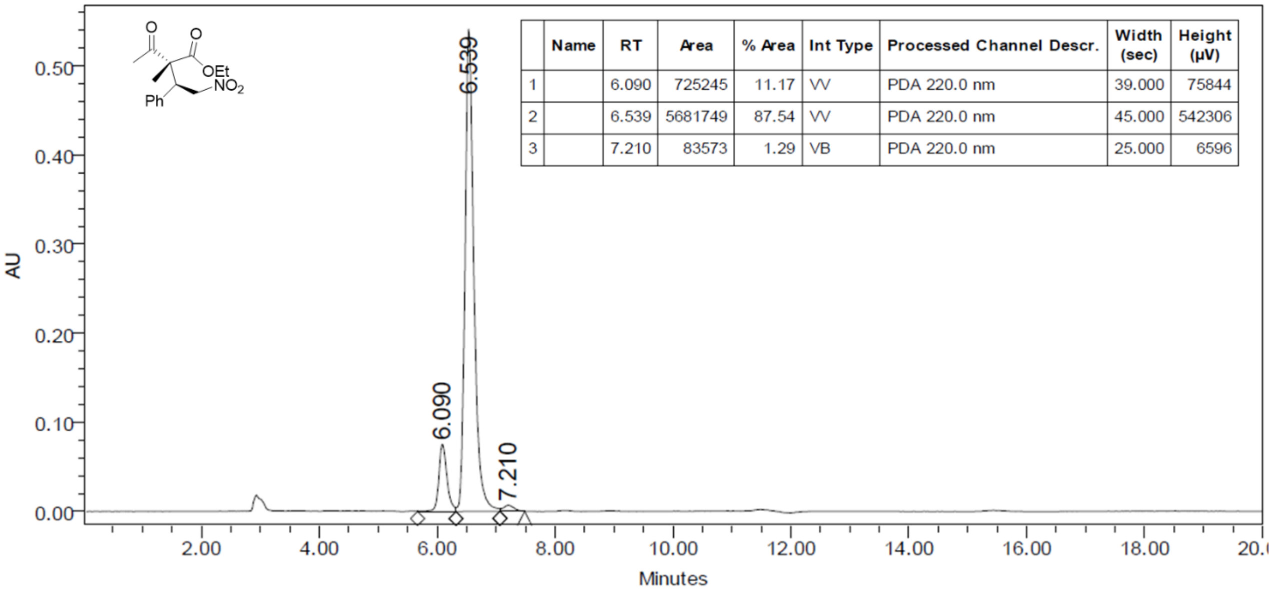

	Name	RT	Area	% Area	Int Type	Processed Channel Descr.	Width (sec)	Height (μV)
1		6.090	725245	11.17	VV	PDA 220.0 nm	39.000	75844
2		6.539	5681749	87.54	VV	PDA 220.0 nm	45.000	542306
3		7.210	83573	1.29	VB	PDA 220.0 nm	25.000	6596

Figure S109: Enantiomerically enriched **6**

7: 96% ee, 98:2 d.r. HPLC Chiralpak IC-3, hexane/ethanol 85:15, 0.6 mL/min., 220 nm.

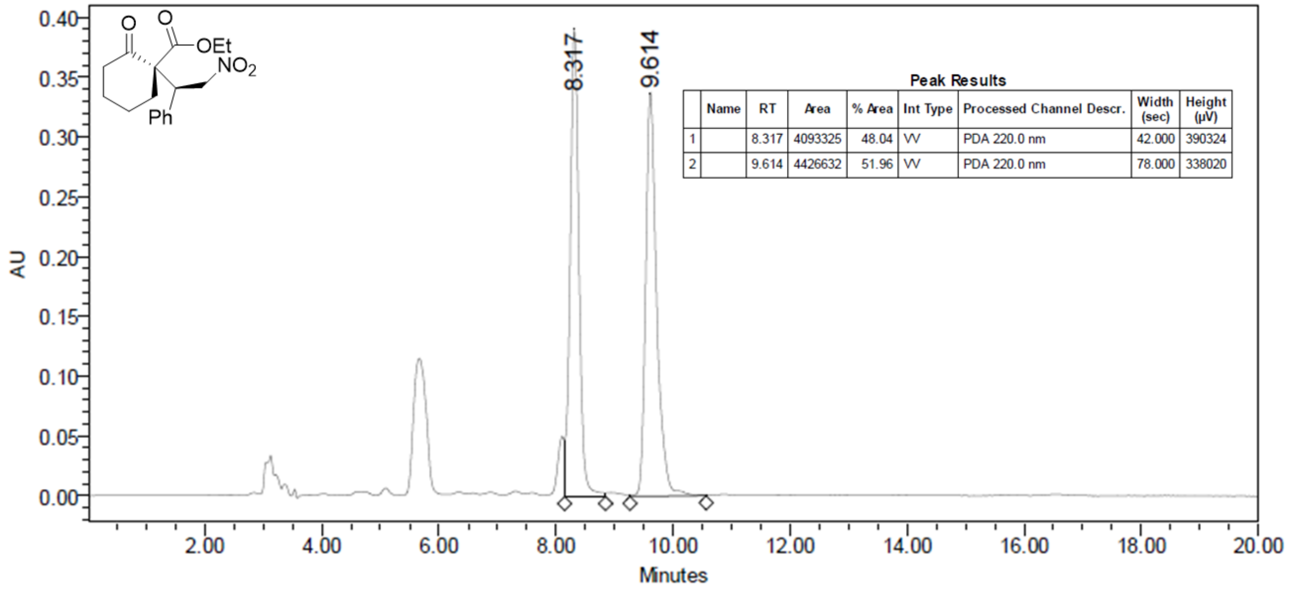

Figure S110: Racemic **7**

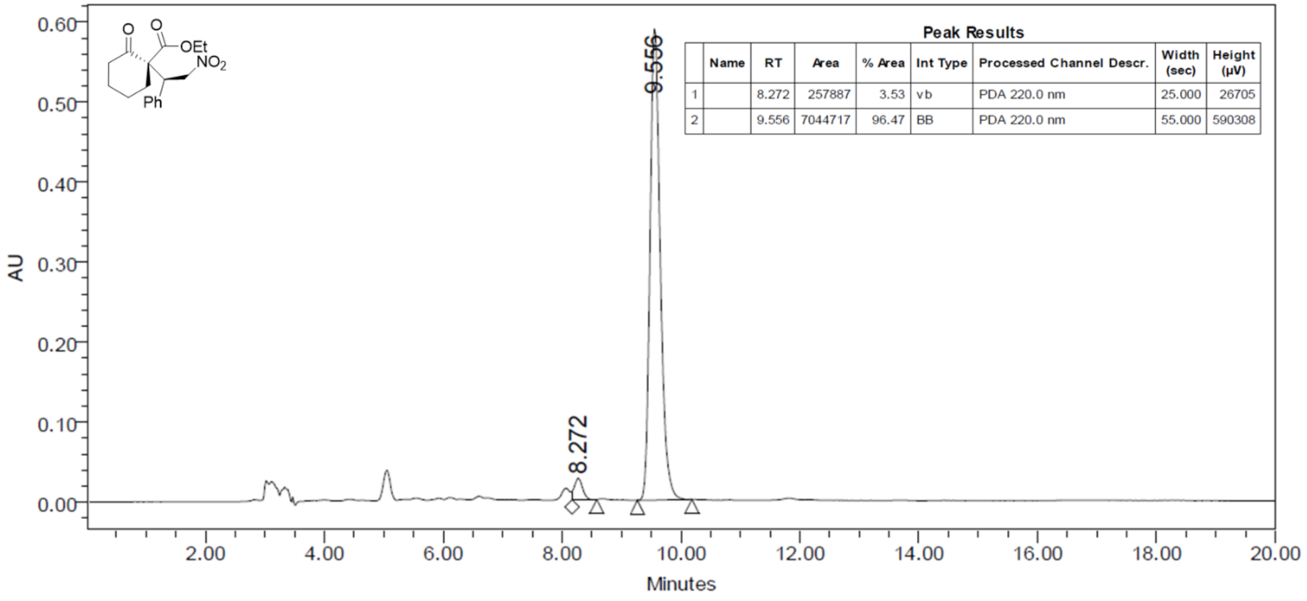

Figure S111: Enantiomerically enriched **7**

8: Major diastereomer: 97% ee, minor diastereomer: 90% ee, 97:3 d.r. HPLC Chiralpak IA, hexane/ethanol 96:04, 0.8 mL/min., 220 nm.

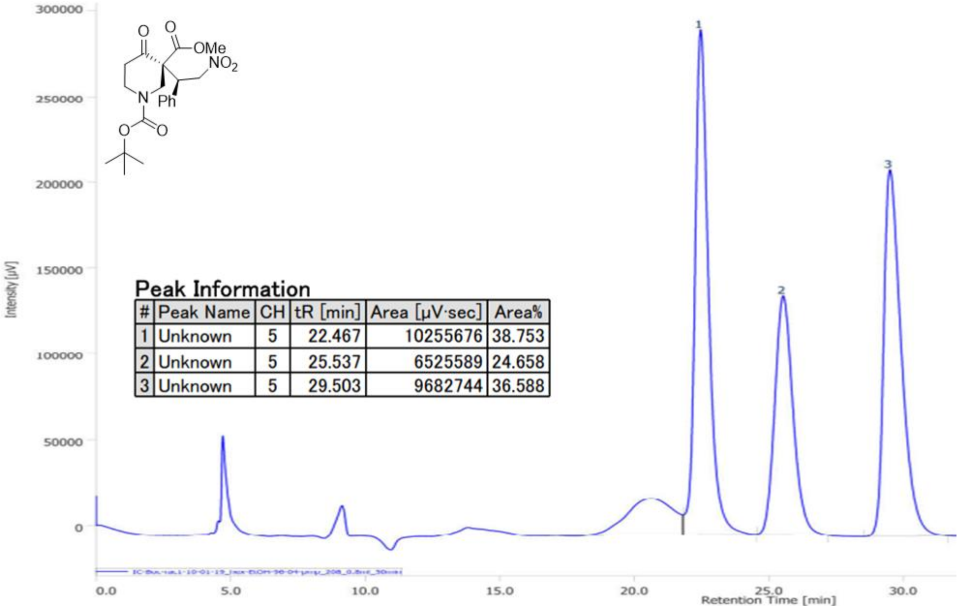

Figure S112: Racemic **8**

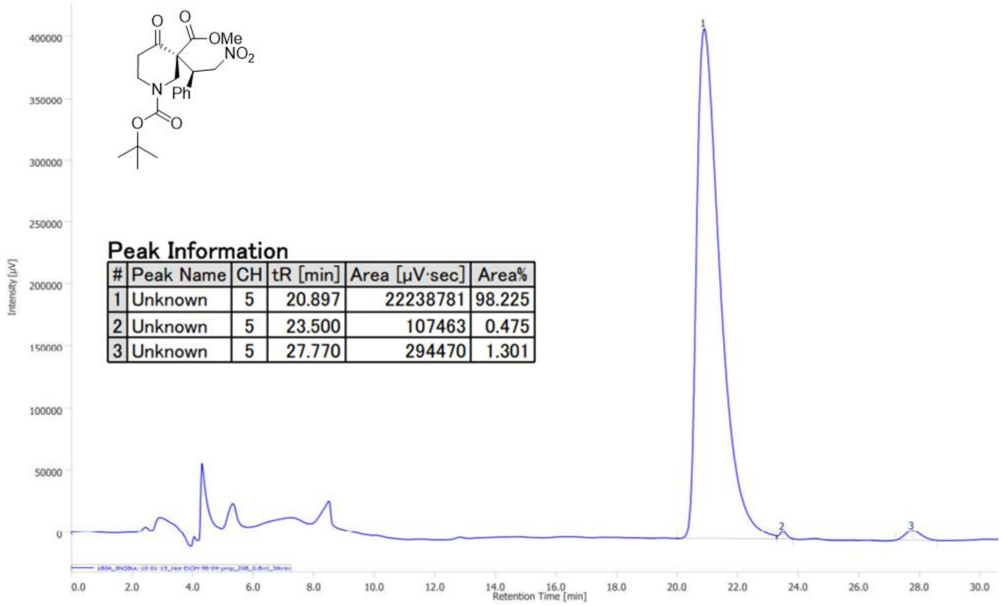

Figure S113: Enantiomerically enriched **8**

9: 96% ee, 85:15 d.r. HPLC Chiralpak AD-H, hexane/ethanol 85:15, 1.0 mL/min., 220 nm.

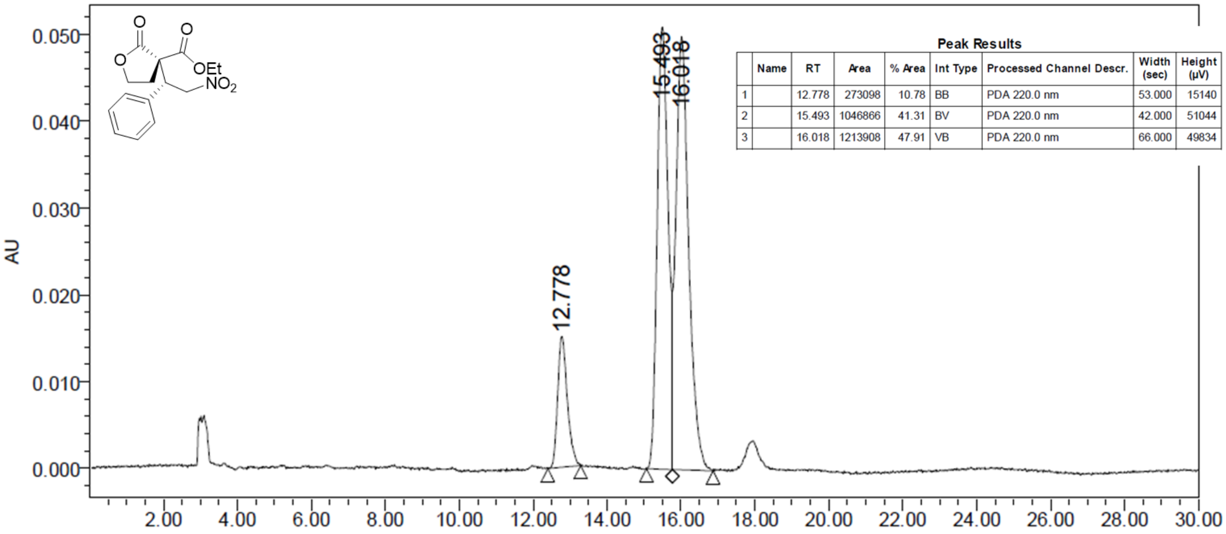

Peak Results

	Name	RT	Area	% Area	Int Type	Processed Channel Descr.	Width (sec)	Height (μV)
1		12.778	273098	10.78	BB	PDA 220.0 nm	53.000	15140
2		15.493	1046866	41.31	BV	PDA 220.0 nm	42.000	51044
3		16.018	1213908	47.91	VB	PDA 220.0 nm	66.000	49834

Figure S114: Racemic **9**

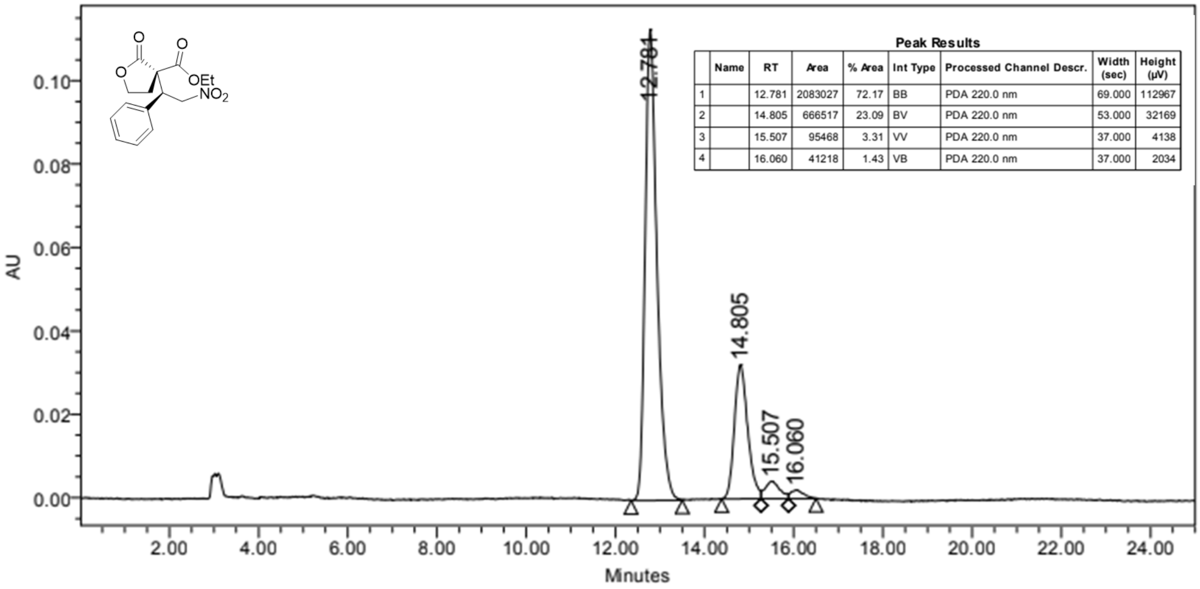

Peak Results

	Name	RT	Area	% Area	Int Type	Processed Channel Descr.	Width (sec)	Height (μV)
1		12.781	2083027	72.17	BB	PDA 220.0 nm	69.000	112967
2		14.805	666517	23.09	BV	PDA 220.0 nm	53.000	32169
3		15.507	95468	3.31	VV	PDA 220.0 nm	37.000	4138
4		16.060	41218	1.43	VB	PDA 220.0 nm	37.000	2034

Figure S115: Enantiomerically enriched **9**

6. References

1. Díaz-Salazar, H.; Jiménez, E. I.; Vallejo Narváez, W. E.; Rocha-Rinza, T.; Hernández-Rodríguez, M. Bifunctional Squaramides with Benzyl-like Fragments: Analysis of CH⋯π Interactions by a Multivariate Linear Regression Model and Quantum Chemical Topology. *Org. Chem. Front*. **2021**, 8 (13), 3217–3227. https://doi.org/10.1039/D0QO01610A.
2. Ričko, S.; Svete, J.; Štefane, B.; Perdih, A.; Golobič, A.; Meden, A.; Grošelj, U. 1,3-Diamine-Derived Bifunctional Organocatalyst Prepared from Camphor. Adv Synth Catal 2016, 358 (23), 3786–3796. https://doi.org/10.1002/adsc.201600498.
3. Cruz-Aguilar, D. A.; Hernández-Rodríguez, M. Stereoselective Synthesis of Highly Substituted 1-Isomorphans (1-Azabicyclo[3.3.1]Nonanes). *Chem. Commun.* **2023**, 59 (58), 8965–8968. https://doi.org/10.1039/D3CC00621B.
4. Li, L.-J.; Zhang, J.-C.; Li, W.-P.; Zhang, D.; Duanmu, K.; Yu, H.; Ping, Q.; Yang, Z.-P. Enantioselective Construction of Quaternary Stereocenters via Cooperative Photoredox/Fe/Chiral Primary Amine Triple Catalysis. *J. Am. Chem. Soc.* **2024**, 146 (13), 9404–9412. https://doi.org/10.1021/jacs.4c01842.
